# PPARG directs trophoblast cell fate and establishment of the uterine-placental interface

**DOI:** 10.1038/s44319-026-00896-0

**Published:** 2026-08-20

**Authors:** Esteban M Dominguez, Ayelen Moreno-Irusta, Khursheed Iqbal, Keting Chen, Alex Finlinson, Marc Parrish, Hiroaki Okae, Takahiro Arima, Geetu Tuteja, Michael J Soares

**Affiliations:** 1 Institute for Reproductive and Developmental Sciences, Department of Pathology & Laboratory Medicine, University of Kansas Medical Center, Kansas City, KS 66160 USA; 2 Department of Genetics, Development, and Cell Biology, Iowa State University, Ames, IA 50011 USA; 3 Department of Obstetrics and Gynecology, University of Kansas Medical Center, Kansas City, KS 66160 USA; 4 Department of Trophoblast Research, Institute of Molecular Embryology and Genetics, Kumamoto University, Kumamoto, 860-0811 Japan; 5 Department of Informative Genetics, Environment and Genome Research Center, Tohoku University Graduate School of Medicine, and Department of Molecular Oncology, Institute of Development, Aging and Cancer, Tohoku University, Sendai, 980-8575 Japan; 6 Present Address: Department of Animal & Food Science, Oklahoma State University, Stillwater, OK 74078 USA

**Keywords:** Development, Urogenital System

## Abstract

The expansion and differentiation of trophoblast stem (TS) cells are critical for defining fundamental properties of the placenta. Specialized trophoblast cells called extravillous trophoblast (EVT) cells in human and invasive trophoblast cells in rat, exit the placenta, enter and transform the uterus. This includes the restructuring of uterine spiral arteries, essential for the transfer of nutrients to the fetus. Mechanisms governing invasive trophoblast cell differentiation remain poorly understood. Here, we investigate peroxisome proliferator-activated receptor gamma (PPARG) as a potential regulator of EVT/invasive trophoblast cell development. In first trimester human placentas, PPARG is expressed in the EVT cell column and increases in amount as human TS cells differentiate into EVT cells. PPARG disruption impairs EVT cell differentiation and alters the expression of genes controlling EVT cell lineage development. Rat invasive trophoblast cells similarly express PPARG. Conditional inactivation of PPARG within rat invasive trophoblast cells establishes PPARG as an essential cell-autonomous regulator of invasive trophoblast cells in vivo. In conclusion, PPARG is a conserved regulator of placentation and is essential for directing trophoblast cell-guided uterine transformation.

## Synopsis

PPARG is a conserved and essential regulator of human EVT cell and rat invasive trophoblast cell differentiation. Complementary human in vitro and rat in vivo models link PPARG to a critical facet of hemochorial placental development and its dysregulation to pregnancy complications associated with defective placentation.

- PPARG expression is enriched in EVT cell columns of first-trimester human placentas and increases during in vitro trophoblast stem cell differentiation into EVT cells.
- Loss of PPARG impairs trophoblast cell differentiation, disrupting EVT cell lineage development, their migratory capacity, and the expression of genes required for EVT cell identity.
- In vivo conditional deletion of PPARG in rat invasive trophoblast cells establishes an essential cell-autonomous role of PPARG in promoting intrauterine trophoblast cell invasion and trophoblast cell-guided uterine transformation.

## Abstract (web summary)

PPARG is a conserved and essential regulator of human EVT cell and rat invasive trophoblast cell differentiation. Complementary human in vitro and rat in vivo models link PPARG to a critical facet of hemochorial placental development and its dysregulation to pregnancy complications associated with defective placentation.

## Graphical abstract

**Figure:**
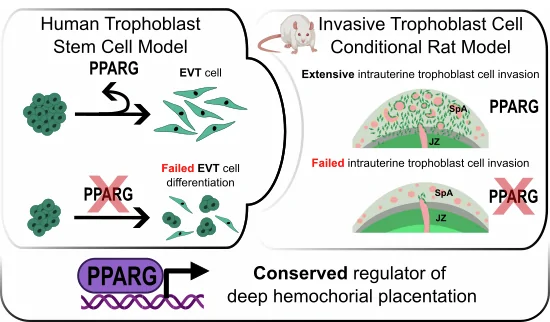

## Introduction

The uterine–placental interface is a dynamic site where uterine and placental structures cooperate to establish a protective environment that redirects maternal resources to support embryonic and fetal development (Georgiades et al, 2002; Maltepe and Fisher, 2015). Both humans and rats possess deep hemochorial placentation, characterized by extensive remodeling of the uterine vasculature to ensure optimal maternal blood supply to the placenta (Pijnenborg et al, 1981; Soares et al, 2012). This vascular transformation is critical for successful pregnancy (Pijnenborg et al, 2006; Aye et al, 2025). Impairments in this process can lead to complications such as pregnancy loss, preeclampsia, intrauterine growth restriction, and preterm birth (Aye et al, 2025; Brosens et al, 2011; Fisher, 2015; Brosens et al, 2019; Harris et al, 2019). These complications significantly contribute to maternal and fetal morbidity and mortality (Fisher, 2015; Brosens et al, 2019; Kaufmann et al, 2003). Furthermore, suboptimal intrauterine conditions can trigger fetal adaptations that increase susceptibility to disease later in life (Calkins and Devaskar, 2011; Gluckman et al, 2008).

Invasive trophoblast cells engineer the remodeling of maternal uterine spiral arteries into distended, low-resistance vessels capable of meeting the increasing demands of the growing fetus (Kaufmann et al, 2003; Harris, 2010). In humans, invasive trophoblast cells are referred to as extravillous trophoblast (EVT) cells (Velicky et al, 2015). These cells take two routes into the uterus. They migrate inside the uterine vasculature and within the uterine stroma situated between uterine blood vessels and are referred to as endovascular invasive trophoblast/EVT cells and interstitial invasive trophoblast/EVT cells, respectively. Endovascular invasive trophoblast/EVT cells replace endothelial cells and adopt a pseudo-endothelial phenotype (Hemberger et al, 2003; Rai and Cross, 2014). Given the critical role of invasive trophoblast/EVT cells in the pregnancy-dependent transformation of the uterus, it is crucial to identify mechanisms that regulate invasive trophoblast/EVT cell development.

Peroxisome proliferator-activated receptor gamma (PPARG) is a member of the nuclear hormone receptor superfamily of ligand-activated transcription factors (Sauer, 2015). PPARG is essential for placental development in the mouse (Barak et al, 1999; Kubota et al, 1999). Deficiency of PPARG results in embryonic lethality at midgestation (Barak et al, 1999; Kubota et al, 1999). In addition, PPARG is expressed in the human placenta and is involved in human trophoblast cell development and regulating key placental functions (Schaiff et al, 2006; Tarrade, 2001; Tarrade et al, 2001; Pavan et al, 2003; Fournier et al, 2007; Schaiff et al, 2000; Shalom-Barak et al, 2004). Among its diverse actions on placentation is its potential involvement in regulating the invasive trophoblast cell lineage. Within rat and human placentation sites, PPARG is prominently expressed in invasive trophoblast/EVT cell lineages (Liu et al, 2018; Vento-Tormo et al, 2018; Scott et al, 2022). PPARG dysregulation is also evident in diseases associated with failures in trophoblast cell-guided uterine spiral artery remodeling, such as preeclampsia and intrauterine growth restriction (Rodie et al, 2005; Waite et al, 2005; Holdsworth-Carson et al, 2010).

In this study, we investigated the involvement of PPARG in EVT cell lineage development in vitro using human trophoblast stem (TS) cells. Human TS cells are a robust in vitro model system that has provided new insights into the regulation of trophoblast cell differentiation (Okae et al, 2018; Varberg et al, 2023; Moreno-Irusta et al, 2026a). The contributions of PPARG to invasive trophoblast cell development were also examined in vivo using a conditionally disrupted *Pparg* rat model. Our findings demonstrate that PPARG is a critical and conserved regulator of invasive trophoblast cell development and invasive trophoblast cell-guided transformation of the uterine vasculature.

## Results

### PPARG is expressed in EVT cells

**Figure 1:**
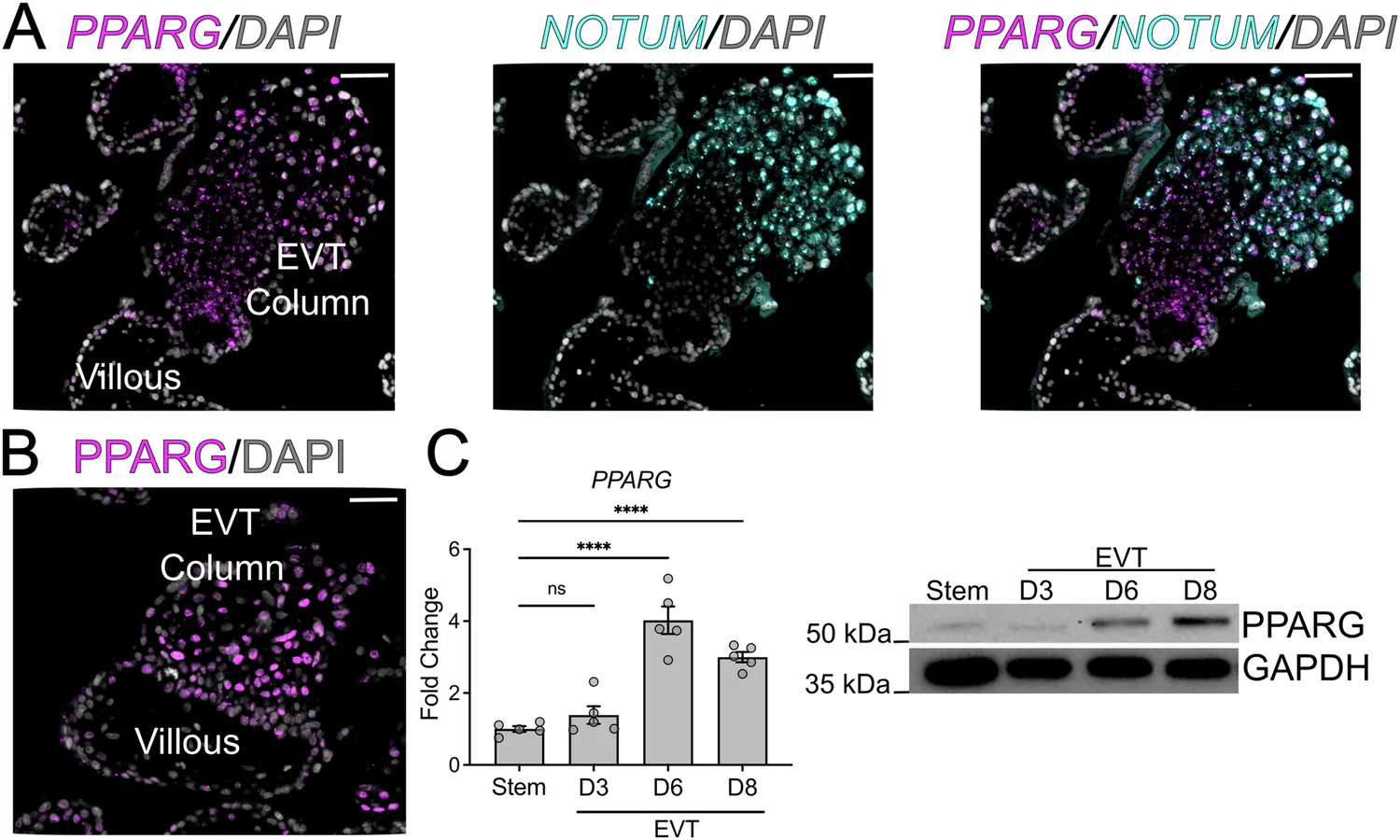
PPARG is expressed in EVT cells. (**A**) *PPARG* transcript (magenta) was localized throughout the EVT column in the first-trimester human placenta (12 weeks) using in situ hybridization. *NOTUM* (cyan) was used as a distal EVT cell-specific transcript. (**B**) PPARG immunohistochemistry (magenta) showed nuclear localization within cells of the first-trimester EVT cell column (12 weeks). Scale bars for panels (**A** and **B**) are 500 μm. (**C**) In vitro *PPARG* transcript and protein expression in CT27 (X,X) TS cells in the stem state, day 3 (D3), day 6 (D6), and day 8 (D8) of EVT cell differentiation (stem state versus D6 or D8, ****p < 0.0001, *n* = 5). Data were presented as mean ± standard error of the mean. Each data point represents a biological replicate. Statistical analysis was performed using ANOVA followed by Dunnett’s post hoc test. Source data are available online for this figure.

**Figure EV1:**
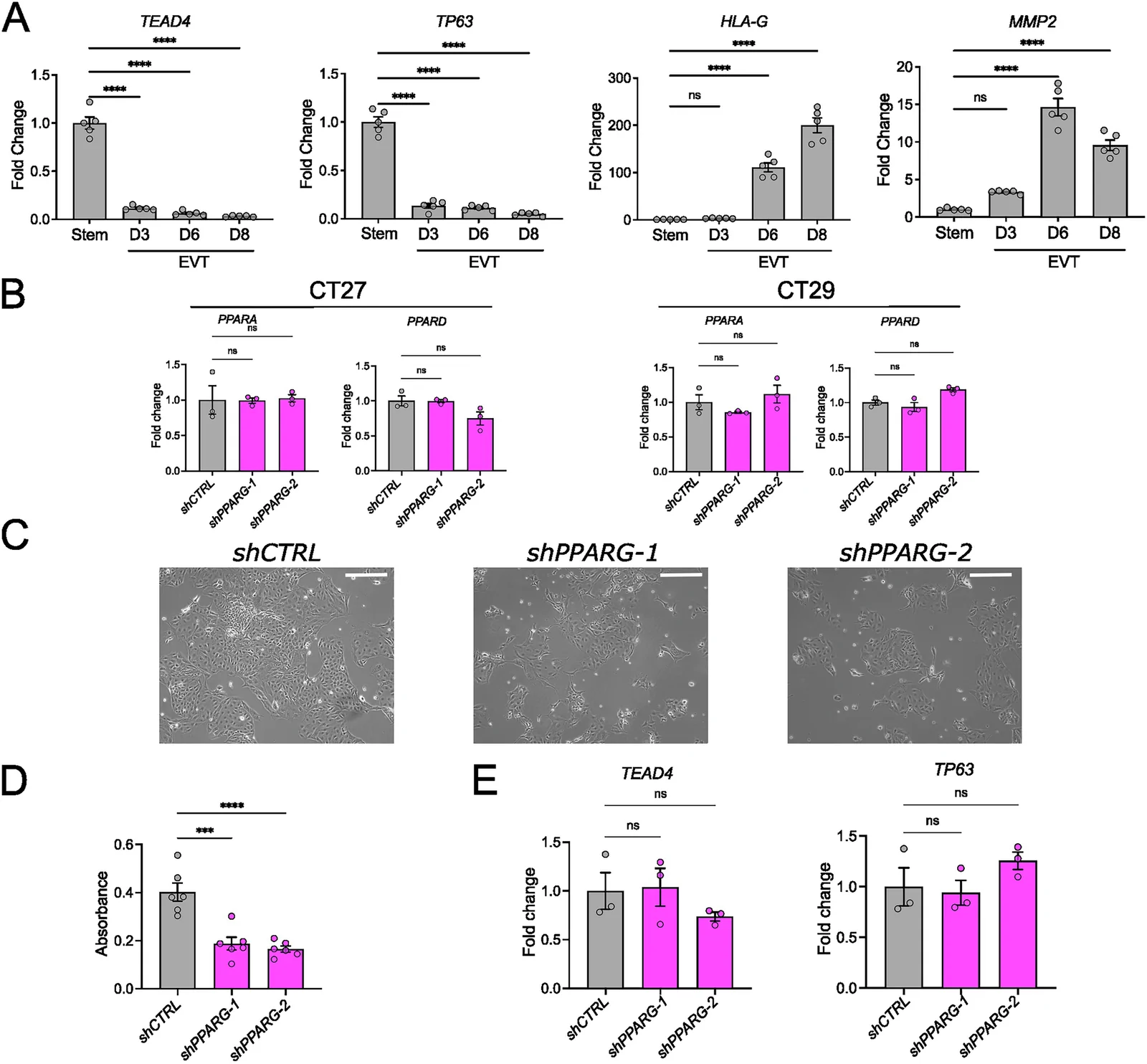
Involvement of PPARG in the behavior of human trophoblast stem (TS) cells. (**A**) Expression of TEAD4 and TP63 (stem-state-associated transcripts), and HLA-G and MMP2 (EVT cell-associated transcripts) in CT27 TS cells in the stem cell state (day 0, D0) and on day 3 (D3), day 6 (D6), and day 8 (D8) of EVT cell differentiation (D0 versus D3, D6, or D8: ****p < 0.0001, *n* = 5). (**B**) PPARA and PPARD transcript levels in CT27 and CT29 TS cells after 8 days of EVT cell differentiation in control shRNA (shCTRL), PPARG shRNA-1 (shPPARG-1), or PPARG shRNA-2 (shPPARG-2) treated cells (ns, nonsignificant; *n* = 3). (**C**) Phase-contrast images of TS cells in the stem state expressing shCTRL, shPPARG-1, or shPPARG-2 (scale bar = 100 µm). (**D**) Quantification of cell proliferation by crystal violet staining in shCTRL, shPPARG-1, or shPPARG-2 treated stem-state TS cells (shCTRL versus shPPARG-1: ***p = 0.0001; shCTRL versus shPPARG-2: ****p < 0.0001, *n* = 6). (**E**)*TEAD4* and *TP63* transcript levels in shCTRL, shPPARG-1, or shPPARG-2 treated TS cells in the stem state (ns nonsignificant; *n* = 3). RT-qPCR was used to measure transcript levels. Data were presented as mean ± standard error of the mean. Each data point represents a biological replicate. Statistical analyses were performed using ANOVA followed by Dunnett’s post hoc test.

Distributions of PPARG transcript and protein in first-trimester human placenta tissue were determined by in situ hybridization and immunohistochemistry, respectively. PPARG transcript (Fig. 1A) and protein (Fig. 1B) were ubiquitously expressed throughout trophoblast cells within villous and extravillous compartments of the human placenta, including EVT cells. The transcript for notum, palmitoleoyl protein carboxylesterase (NOTUM), an EVT cell-associated transcript (Shukla et al, 2024), co-localized with PPARG transcripts in the distal region of the EVT cell column (Fig. 1A). PPARG protein was located within nuclei of EVT cells (Fig. 1B). These observations are consistent with previous reports (Tarrade et al, 2001; Waite et al, 2000). PPARG expression was also examined in CT27 (X,X) human TS cells, in the stem state and after EVT cell differentiation (Okae et al, 2018). Expression of *TEAD4* and *TP63* defined the stem state of these cells, whereas *HLA-G* and *MMP2* expression characterized EVT cell differentiation (Fig. EV1A). PPARG transcript and protein expression increased from the stem state to the EVT cell differentiation state (Fig. 1C). Thus, PPARG is expressed in EVT cells of the developing human placenta and in human EVT cells following their differentiation from TS cells.

### PPARG effects on EVT cell differentiation

**Figure 2:**
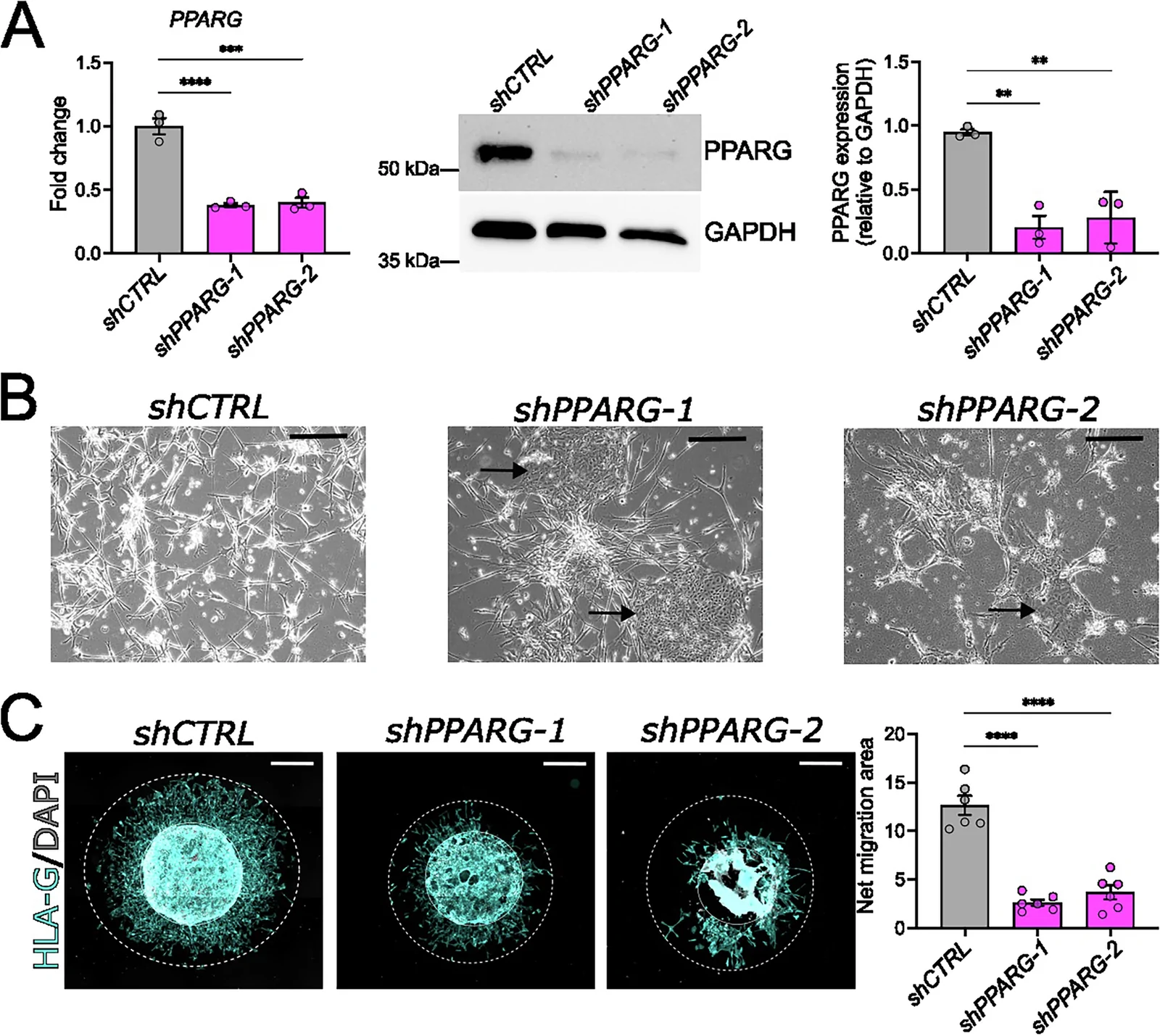
PPARG regulates EVT cell differentiation. (**A**) In vitro *PPARG* transcript and protein levels after 8 days of EVT cell differentiation in control shRNA (shCTRL) versus PPARG shRNA-1 (shPPARG-1) and PPARG shRNA-2 (shPPARG-2) treated cells (*PPARG* transcript for shCTRL versus shPPARG-1: ****p < 0.0001 and shCTRL versus shPPARG-2: ***p = 0.0001; PPARG protein for shCTRL versus shPPARG-1: **p = 0.0015 and shCTRL versus shPPARG-2: **p = 0.0027, *n* = 3). (**B**) Phase-contrast images showing the morphology of EVT cells expressing shCTRL, shPPARG-1, and shPPARG-2. Black arrows indicate clusters of cells exhibiting a stem cell-like morphology, scale bar = 100 μm. (**C**) HLA-G immunofluorescence detection of cell migration in shCTRL versus shPPARG-1 and shPPARG-2 treated cells (shCTRL versus shPPARG-1 and shPPARG-2, ****p < 0.0001, *n* = 6), scale bar = 500 μm. White dashed lines demarcate the area of trophoblast cell migration. Data were presented as the mean ± standard error of the mean. Each data point represents a biological replicate. Statistical analysis was performed using one-way ANOVA followed by Dunnett’s post hoc tests. Source data are available online for this figure.

**Figure EV2:**
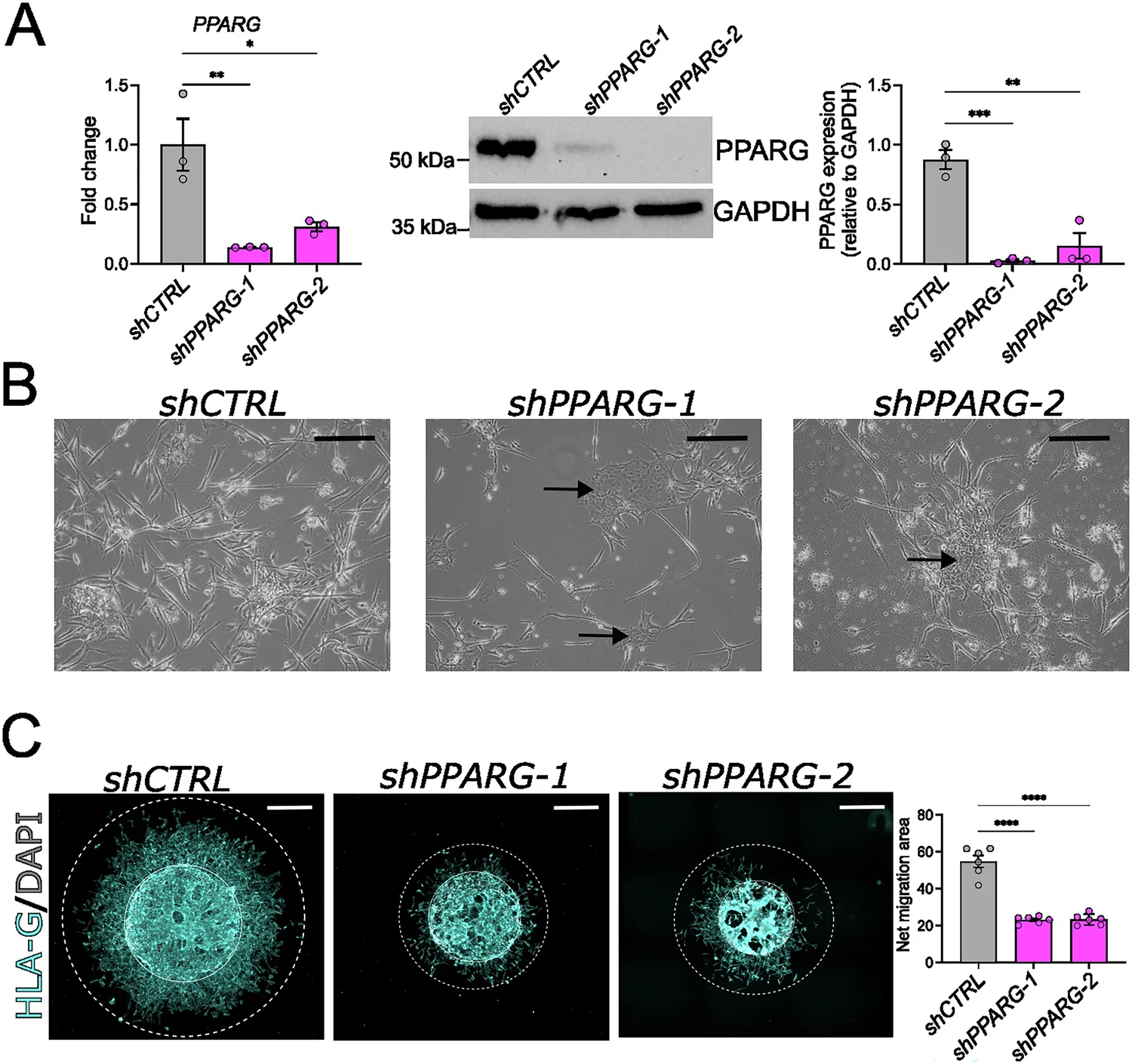
Involvement of PPARG in the differentiation of CT29 (X,Y) trophoblast stem (TS) cells to extravillous trophoblast (EVT) cells. (**A**) PPARG transcript and protein levels in CT29 TS cells after 8 days of EVT cells differentiation in control shRNA (shCTRL), PPARG shRNA-1 (shPPARG-1), or PPARG shRNA-2 (shPPARG-2) treatments (*PPARG* transcript for shCTRL versus shPPARG-1: **p = 0.0056 and shCTRL versus shPPARG-2: *p = 0.0157; PPARG protein for shCTRL versus shPPARG-1: ***p = 0.0004 and shCTRL versus shPPARG-2: **p = 0.0010; *n* = 3). (**B**) Phase-contrast images of cells expressing shCTRL, shPPARG-1, or shPPARG-2 shRNAs (scale bar = 100 µm). Black arrows indicate cells exhibiting stem cell-like morphology. (**C**) HLA-G immunofluorescence detection of migrating cells (scale bar = 500 µm) and corresponding quantification of net migration area during exposure to EVT cell differentiation conditions in shCTRL, shPPARG-1, or shPPARG-2 treated cells (shCTRL versus shPPARG-1 or shPPARG-2: ****p = <0.0001; *n* = 6). RT-qPCR was used to measure transcript levels. Data were presented as mean ± standard error of the mean. Each data point represents a biological replicate. Statistical analysis was performed using ANOVA followed by Dunnett’s post hoc test. White dashed lines demarcate areas of trophoblast cell migration.

**Figure EV3:**
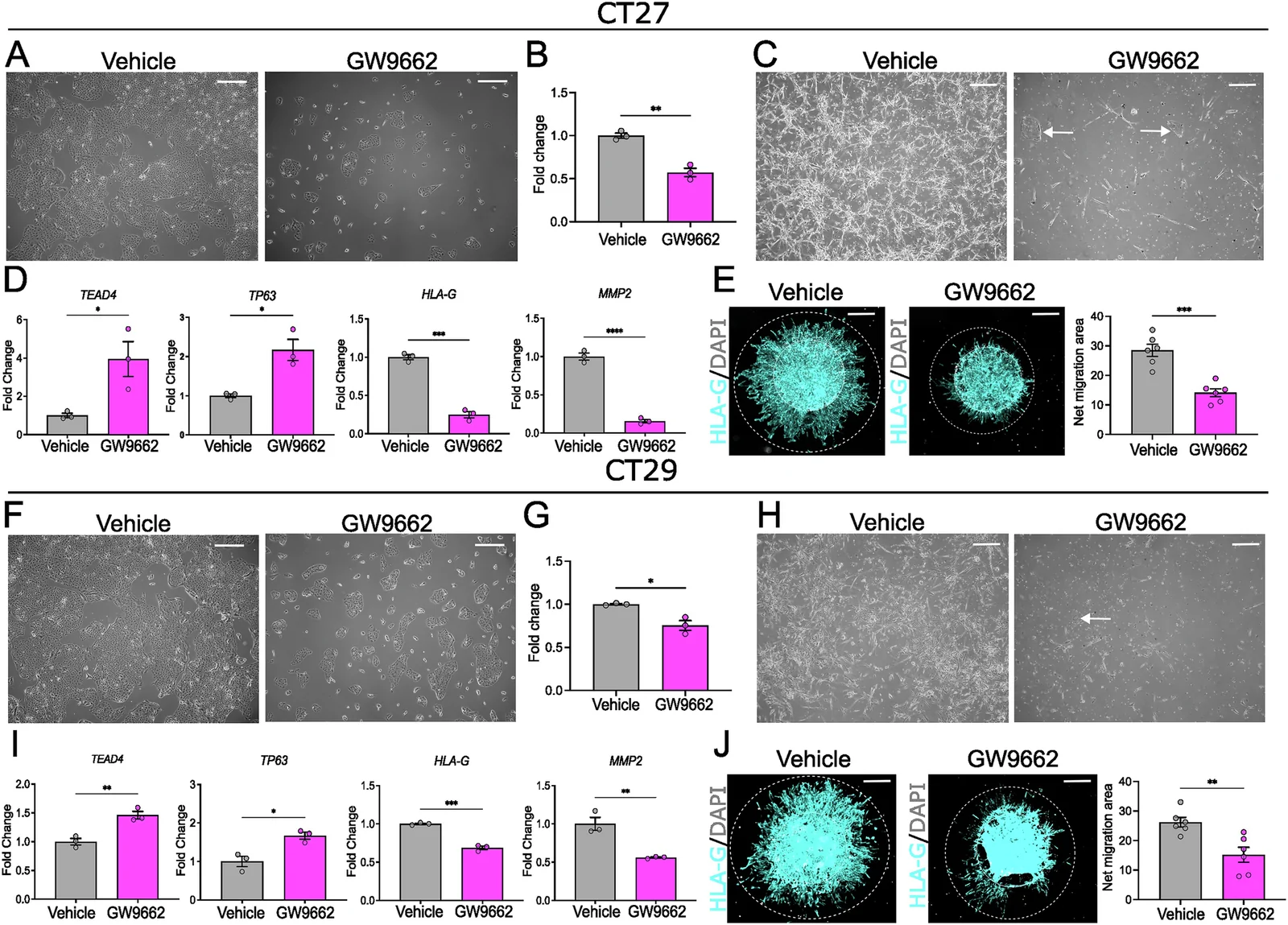
Effects of a PPARG antagonist (GW9662) on EVT cell differentiation. (**A**) Phase-contrast images of CT27 TS cells maintained in the stem cell state or treated with Vehicle (dimethyl sulfoxide) or GW9662 (10 µM), scale bar = 100 µm. (**B**) Quantification of cell numbers in Vehicle or GW9662-treated CT27 TS cells (Vehicle versus GW9662: **p = 0.0018, *n* = 3). (**C**) Phase-contrast images of CT27 TS cells exposed to EVT cell differentiation conditions and treated with Vehicle or GW9662, scale bar = 100 µm. White arrows indicate cells exhibiting stem state-like morphology. (**D**) RT-qPCR measurements of *TEAD4* and *TP63* (stem-state-associated transcripts) and *HLA-G* and *MMP2* (EVT cell-associated transcripts) in Vehicle or GW9662-treated CT27 TS cells following 8 days of exposure to EVT cell differentiation culture conditions (Vehicle versus GW9662: *TEAD4*, *p = 0.0325; *TP63*, *p = 0.0131; *HLA-G*, ***p = 0.0001; *MMP2*, ****p = <0.0001; *n* = 3). (**E**) HLA-G immunofluorescence detection of migrating CT27 TS cells (scale bar = 500 µm) and corresponding quantification of net migration area during EVT cell differentiation in Vehicle or GW9662-treated cells (Vehicle versus GW9662: ***p = 0.0002, *n* = 6). White dashed lines demarcate the area of trophoblast cell migration. (**F**) Phase-contrast images of CT29 TS cells maintained in the stem cell state and treated with Vehicle or GW9662, scale bar = 100 µm. (**G**) Quantification of CT29 TS cell numbers treated with Vehicle or GW9662 (Vehicle versus GW9662: *p = 0.0121, *n* = 3). (**H**) Phase-contrast images of CT29 TS cells exposed to EVT cell differentiation conditions and treated with Vehicle or GW9662, scale bar = 100 µm. White arrows indicate cells exhibiting stem state-like morphology. (**I**) RT-qPCR measurements of *TEAD4* and *TP63* (stem-state-associated transcripts) and HLA-G and MMP2 (EVT cell-associated transcripts) in Vehicle or GW9662-treated CT29 TS cells following 8 days of exposure to EVT cell differentiation culture conditions (Vehicle versus GW9662: *TEAD4*, **p = 0.0060; *TP63*, *p = 0.0148; *HLA-G*, ***p = 0.0002; *MMP2*, **p = 0.0067, *n* = 3). (**J**) HLA-G immunofluorescence detection of migrating CT29 TS cells (scale bar = 500 µm) and corresponding quantification of net migration area during EVT cell differentiation in Vehicle or GW9662-treated cells (Vehicle versus GW9662: **p = 0.0043, *n* = 6). White dashed lines demarcate the area of trophoblast cell migration. Data were presented as mean ± standard error of the mean. Each data point represents a biological replicate. Statistical analysis was performed using unpaired *t*-tests.

The expression of PPARG within EVT cells prompted an investigation into a possible role for PPARG in EVT cell development. A lentiviral-mediated short hairpin RNA (shRNA) loss-of-function strategy was used to inhibit PPARG in CT27 (X,X) TS cells. PPARG transcript and protein were significantly decreased in EVT cells stably expressing either of two different PPARG shRNAs when compared to expression of a control shRNA (Fig. 2A). The specificity of PPARG-targeting shRNAs was assessed by monitoring the expression of other members of the PPAR family. Expression of *PPARA* and *PPARD* transcripts were not affected following treatment with PPARG shRNAs (Fig. EV1B). Treatment with PPARG shRNAs slowed TS cell proliferation without changes in stem state-associated markers TEAD4 and TP63 (Fig. EV1C–E). Disruption of PPARG affected the morphology of the EVT cell differentiation state, including a decrease in cell elongation and the presence of stem state-like cell clusters (Fig. 2B). Cells expressing PPARG shRNAs showed significantly less migration than EVT cells expressing a control shRNA (Fig. 2C). Similar findings were observed in CT29 (X,Y) TS cells following their manipulation with PPARG shRNAs (Fig. EV2A–C). Additionally, exposure to a small molecule PPARG antagonist (GW9662) similarly negatively affected stem state proliferation, EVT cell differentiation, and cell migration (Fig. EV3A–J). Collectively, the findings indicate that PPARG possesses a fundamental role in regulating EVT cell development and function.

### PPARG effects on trophoblast organoids

**Figure EV4:**
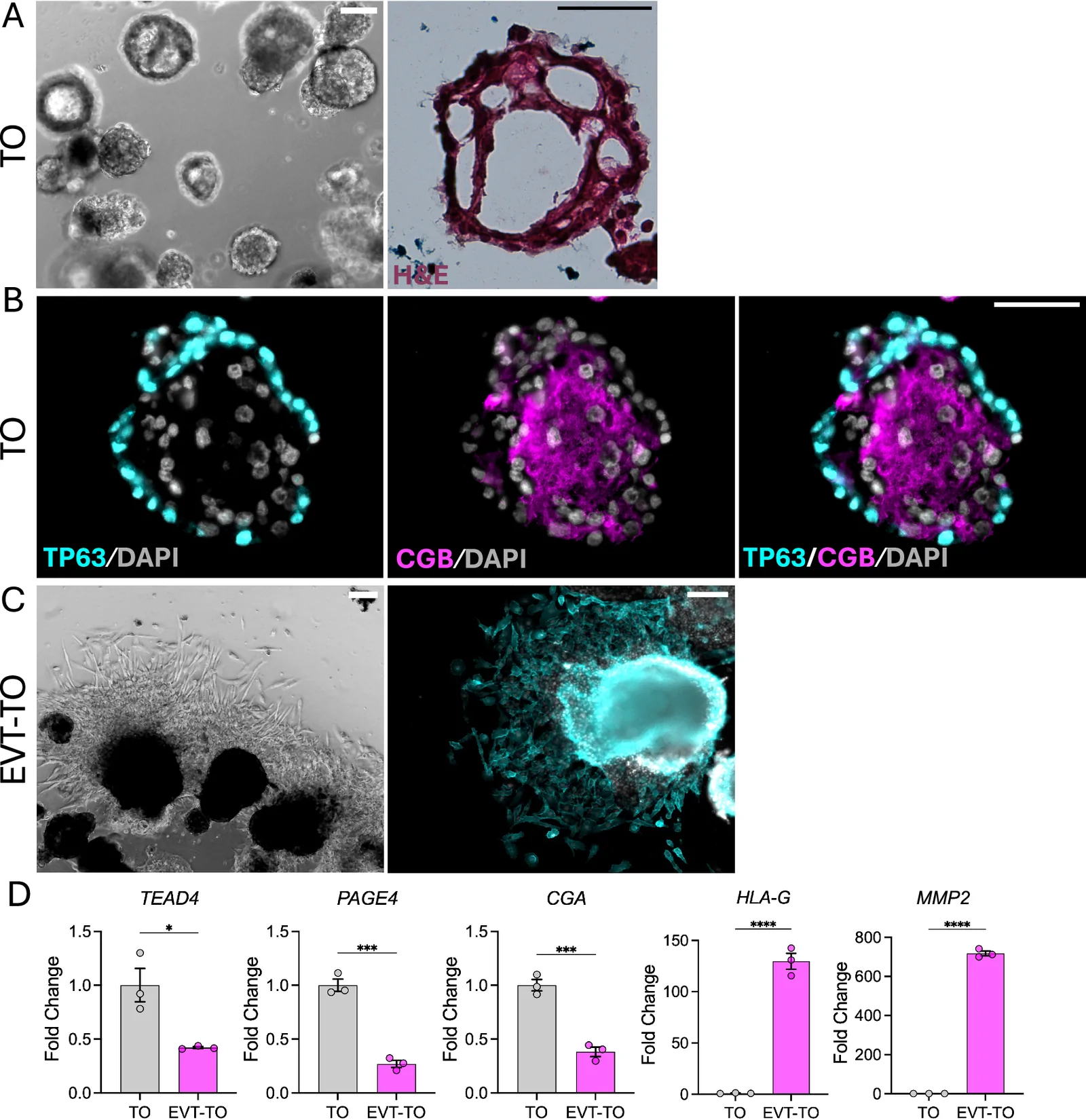
Characterization of TS cell-derived trophoblast organoids (TO). (**A**) Phase-contrast image and hematoxylin and eosin staining of TO, left panel: scale bar = 200 and right panel: scale bar = 50 μm. (**B**) TP63 (cyan) and CGB (magenta) immunodetection in TOs, scale bar = 50 μm. (**C**) HLA-G immunocytochemistry in TOs transferred to EVT cell differentiation conditions (EVT-TO), left panel: scale bar = 200 and right panel: scale bar = 50 μm. (**D**) RT-qPCR of stem state, syncytiotrophoblast and EVT cell-associated transcripts in TOs in the stem state and following exposure to EVT-TO (stem state versus EVT-TO: *TEAD4*, *p = 0.0126; PAGE4, ***p = 0.0001; *CGA*, ***p = 0.0001; *HLA-G*, ****p < 0.0001; *MMP2*, ****p < 0.0001, *n* = 3). Data were presented as mean ± standard error of the mean. Each data point represents a biological replicate. Statistical analysis was performed using unpaired *t*-tests.

**Figure 3:**
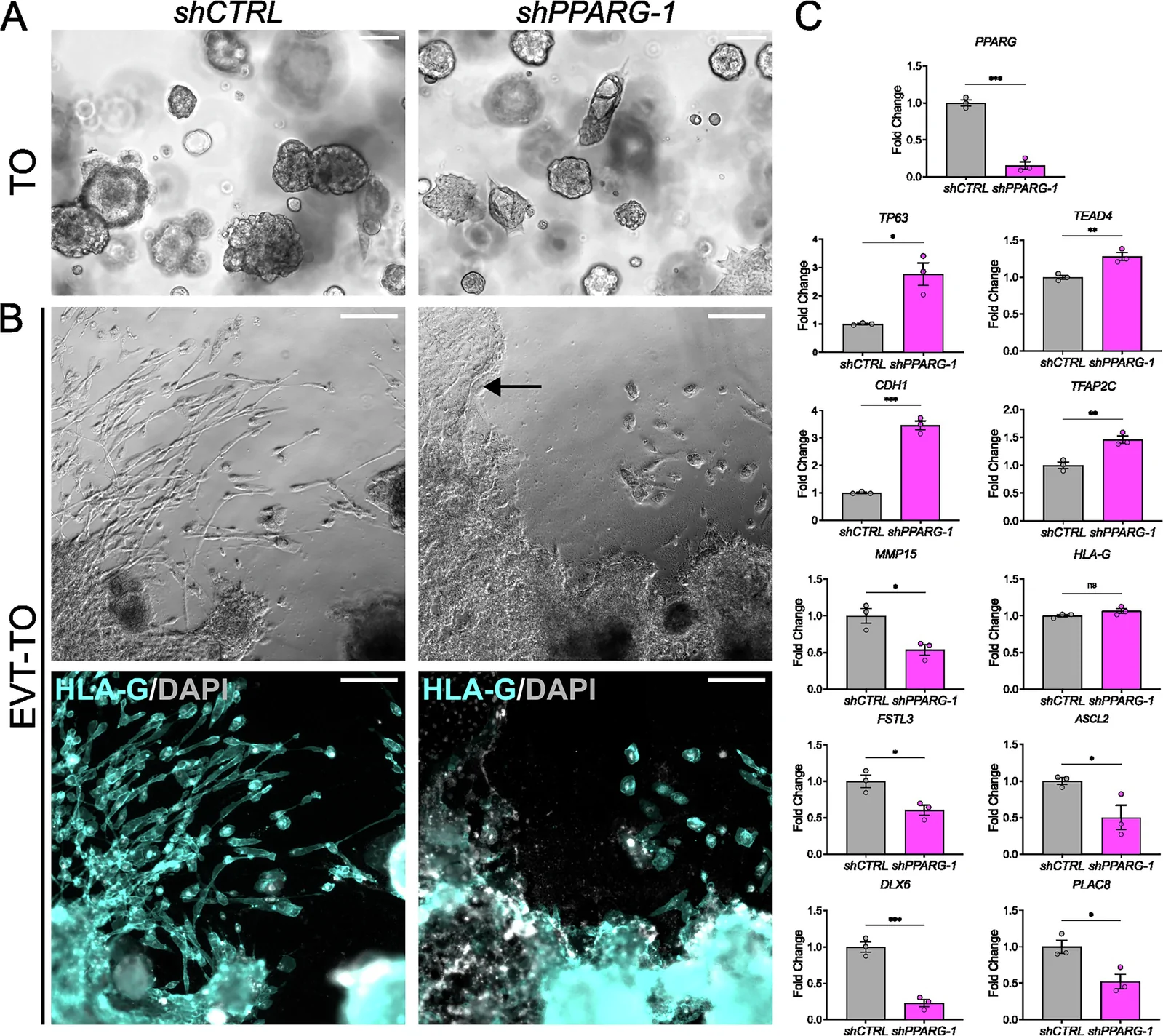
Examination of the role of PPARG in EVT cell differentiation using trophoblast organoids. (**A**) Phase-contrast images of trophoblast organoids (TO) expressing control shRNA (shCTRL) or PPARG shRNA-1 (shPPARG-1), scale bar = 200 μm. (**B**) Phase-contrast (top panel) and HLA-G immunostaining (bottom panel) images of shCTRL and shPPARG-1 TOs transferred to EVT cell differentiation conditions (EVT-TO). The black arrow indicates the location of cells exhibiting stem cell-like morphology, scale bar = 200 μm. (**C**) RT-qPCR analysis of shCTRL versus shPPARG-1 TOs exposed to EVT cell differentiation conditions (shCTRL versus shPPARG-1: *PPARG*, ***p = 0.0002; *TP63*, *p = 0.0113; *TEAD4*, **p = 0.0093; *CDH1*, ***p = 0.0001; *TFAP2C*, **p = 0.0060; *MMP15*, *p = 0.0199; *FSTL3*, *p = 0.0230; *ASCL2*, *p = 0.0440; *DLX6*, ***p = 0.0009; *PLAC8*, *p = 0.0242; *n* = 3). Data were presented as the mean ± standard error of the mean. Each data point represents a biological replicate. Statistical analyses were performed using unpaired *t*-tests. Source data are available online for this figure.

We next examined the involvement of PPARG in the establishment and behavior of TS cell-derived trophoblast organoids. We initially validated the TS cell-derived trophoblast organoid culture system (Fig. EV4A–D) and then examined control or PPARG-deficient trophoblast organoids. The ability to form trophoblast organoids was not affected by PPARG disruption (Fig. 3A); however, PPARG inhibition did affect EVT cell differentiation from trophoblast organoids (Fig. 3B). This finding was supported by inspection of the morphology of the trophoblast organoids and their expression of TS cell stem state transcripts (*TEAD4, TFAP2C, TP63, CDH1*) and EVT cell-associated transcripts (*MMP15, HLA-G, FSTL3, ASCL2, DLX6, PLAC8*) (Fig. 3C).

### Identification of PPARG gene targets in EVT cells

**Figure 4:**
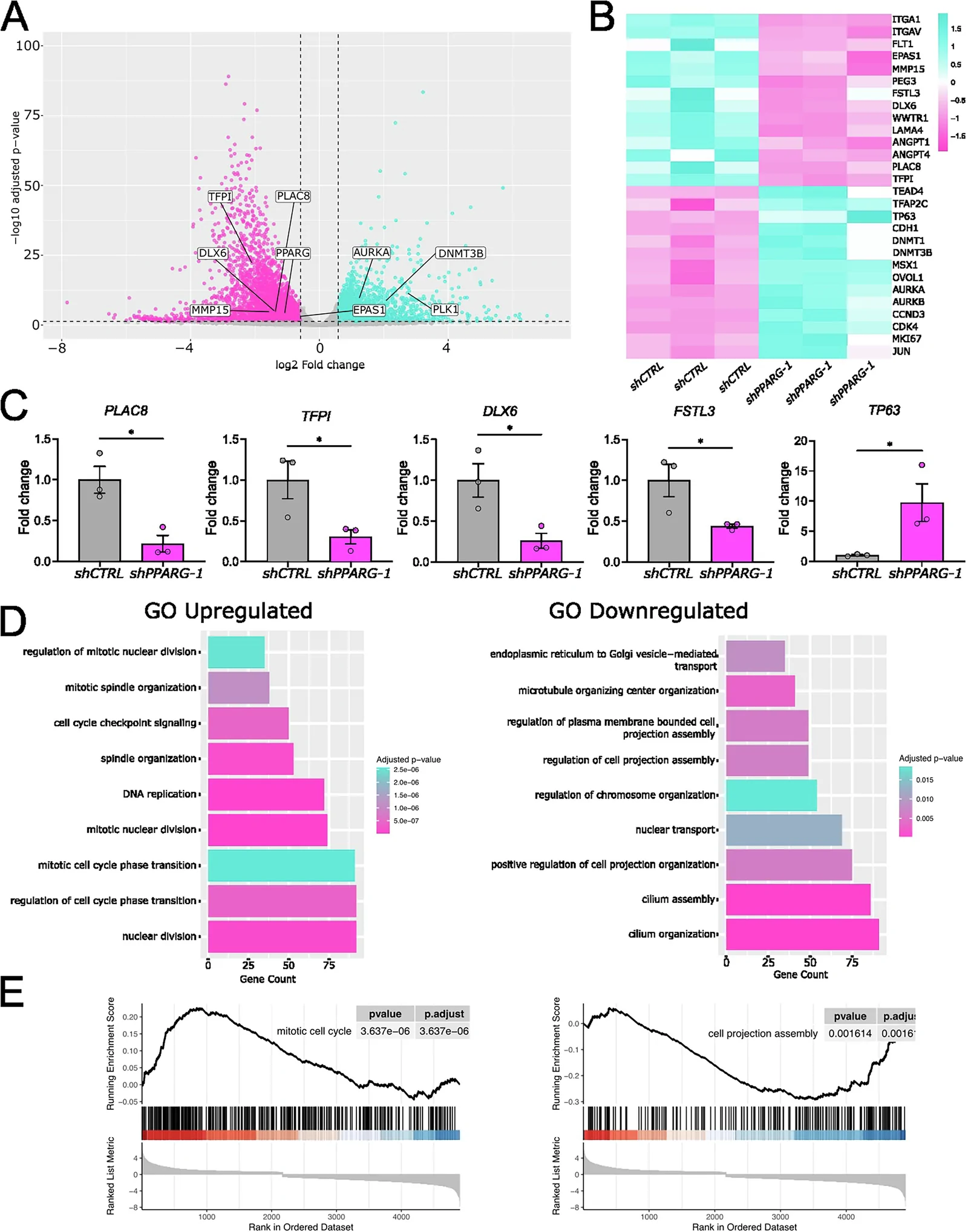
Effects of PPARG on the EVT cell transcriptome. (**A**) Volcano plot and (**B**) heat map depicting RNA-seq analysis of control shRNA (shCTRL) and PPARG shRNA-1 (shPPARG-1) for cells exposed to culture conditions used to promote EVT cell differentiation (*n* = 3 per group). Downregulated transcripts are represented by magenta dots (*p* < 0.05, fold change <1.5), and cyan dots represent upregulated transcripts (*p* < 0.05, fold change >1.5). Z-scores of total read values are represented in the heat map. (**C**) RT-qPCR validation of putative PPARG targets in shCTRL versus shPPARG-1 treated cells (shCTRL versus shPPARG-1: *PLAC8*, *p = 0.0156; *TFPI*, *p = 0.0468; *DLX6*, *p = 0.0304; *FSTL3*, *p = 0.0489; *TP63*, *p = 0.0489; *n* = 3) (**D**) Gene ontology (GO) analyses of upregulated and downregulated genes in shCTRL and shPPARG-1 treated TS cells exposed to culture conditions used to promote EVT cell differentiation. Statistical analyses were performed using Fisher’s exact test. (**E**) Gene set enrichment analyses of upregulated and downregulated genes in control and PPARG shRNA-treated TS cells exposed to culture conditions used to promote EVT cell differentiation. Statistical analyses were performed using the Kolmogorov–Smirnov test. The CT27 (X,X) TS cell line was used for the analyses. Data were presented as the mean ± standard error of the mean. Each data point represents a biological replicate. Statistical analyses were performed using unpaired *t*-tests. Source data are available online for this figure.

PPARG is a transcription factor, which prompted an examination of transcripts affected by PPARG disruption. RNA sequencing (RNA-seq) was performed, and transcriptomic profiles were analyzed following 8 days of EVT cell differentiation in control shRNA and PPARG shRNA-1-treated cells (Dataset EV1). A total of 5845 differentially expressed genes were identified in control versus PPARG shRNA-1-treated cells (*p* < 0.05, and fold change of 1.5), including 2510 upregulated and 3335 downregulated genes. The differential gene expression profile is presented as a volcano plot (Fig. 4A). Selected upregulated and downregulated genes connected to trophoblast cell biology are shown in a heat map (Fig. 4B). We identified upregulation of several genes associated with the TS cell stem state (e.g., *TEAD4, TFAP2C, TP63*, and *CDH1*) and downregulation of several EVT cell-associated genes (e.g., *ITGA1, ITGAV, FLT1, EPAS1, DLX6, PLAC8, TFPI*, and *FSTL3*). These findings were independently validated by reverse transcriptase-quantitative polymerase chain reaction (RT-qPCR, Fig. 4C; Appendix Fig. S1). Gene ontology (GO) (Fig. 4D; Datasets EV2 and EV3) and gene set enrichment analysis (GSEA) (Fig. 4E; Dataset EV4) implicated PPARG in the regulation of cell cycle-associated genes and cell projection assembly-associated genes. The latter cellular process directly connects PPARG with cell elongation and motility that are acquired as TS cells transit from the stem state to EVT cells.

**Figure 5:**
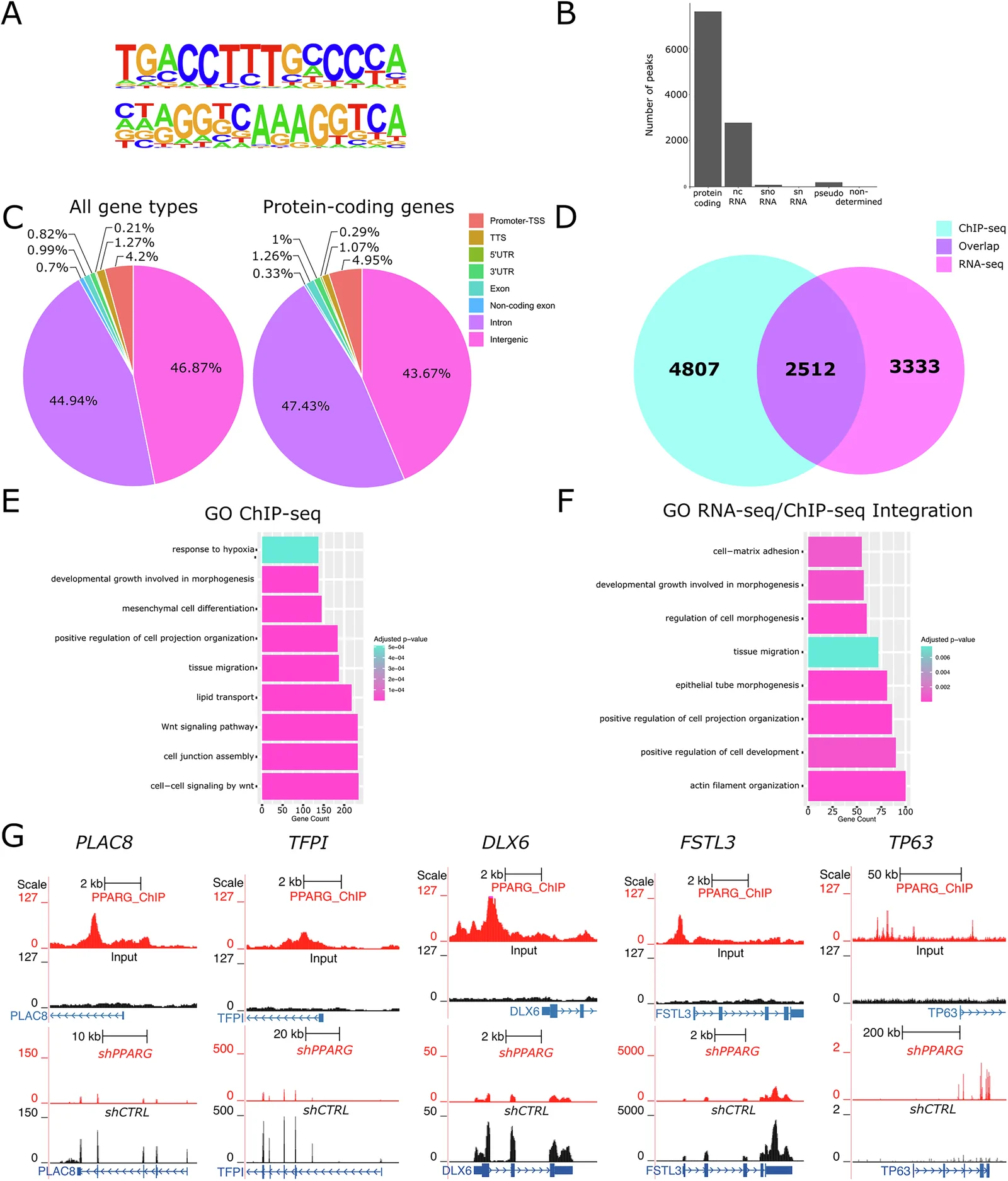
PPARG targets within the EVT cell genome identified using ChIP-seq. (**A**) Two significantly enriched PPARG binding motifs were observed within PPARG ChIP-seq peaks. (**B**) Number of peaks observed within specific locations of the genome. (**C**) Pie chart showing the distribution of PPARG peaks in genomic regions for all genes and protein-coding genes. (**D**) Venn diagram showing the integration of PPARG-responsive genes identified by RNA-seq (see Fig. 4) and PPARG gene targets identified by PPARG ChIP-seq of EVT cells. (**E**) Gene ontology (GO) analysis of the PPARG ChIP-seq dataset performed on EVT cells. Statistical analyses were performed using Fisher’s exact test. (**F**) GO analysis of an integrated dataset generated from PPARG-responsive genes identified by RNA-seq (see Fig. 4) and PPARG gene targets identified by PPARG ChIP-seq of EVT cells. Statistical analyses were performed using Fisher’s exact test. (**G**) Integration of PPARG ChIP-Seq peaks and RNA-seq track at selected gene loci that exhibit sensitivity to PPARG inhibition. Visualization was performed using the University of California at Santa Cruz Genome Browser. Red peaks represent PPARG-bound regions for the ChIP-seq and associated RNA-seq analysis from shPPARG-1 treated TS cells, whereas black peaks represent input DNA ChIP-seq and RNA-seq analysis from the shCTRL treated TS cells.

Since, PPARG is a transcription factor, we next analyzed genome-wide PPARG-DNA interactions using chromatin immunoprecipitation sequencing (ChIP-seq). We identified 7319 genes with at least one PPARG-bound region (peak) in differentiated EVT cells (Dataset EV5). The two most prevalent motifs identified within the peaks were known PPARG DNA binding motifs (peroxisome proliferator response element, PPRE) and primarily consisted of a direct repeat 1 (DR1) sequence (Fig. 5A). Most PPARG-DNA peaks (~7000) were located within introns or intergenic regions of protein-coding genes (Fig. 5B,C), suggesting the involvement of PPARG in the regulation of enhancer activity. Integration of RNA-seq (5,845 differentially expressed genes) and ChIP-seq (7319 genes with PPARG peak) datasets resulted in the identification of 2512 potential direct PPARG gene targets (Fig. 5D; Dataset EV6). GO analyses of PPARG gene targets determined by PPARG ChIP-seq (Fig. 5E; Dataset EV7) and an integrated dataset of PPARG-responsive genes determined by RNA-seq of EVT cells (see Fig. 4), and PPARG gene targets determined by PPARG ChIP-seq (Fig. 5F; Dataset EV8) led to the identification of genes associated with positive regulation of cell projection organization and tissue migration. These represent cellular processes associated with EVT cell differentiation. In addition, GO analysis identified PPARG target genes connected to response to hypoxia, lipid transport, and WNT signaling, which are also likely important in the transition from the stem state to differentiated EVT cells (Fig. 5E). Evidence supporting *PLAC8*, *TFPI*, *DLX6*, *FSTL3* and *TP63*, which are known regulators of trophoblast cell differentiation (Chang et al, 2018; Xie et al, 2018; Muto et al, 2021; Wang et al, 2022; Kim et al, 2024), as potential direct targets of PPARG is shown (Fig. 5G). Collectively, the results support PPARG regulating genes that control the development of the EVT cell lineage.

### PPARG expression within the rat uterine–placental interface

**Figure 6:**
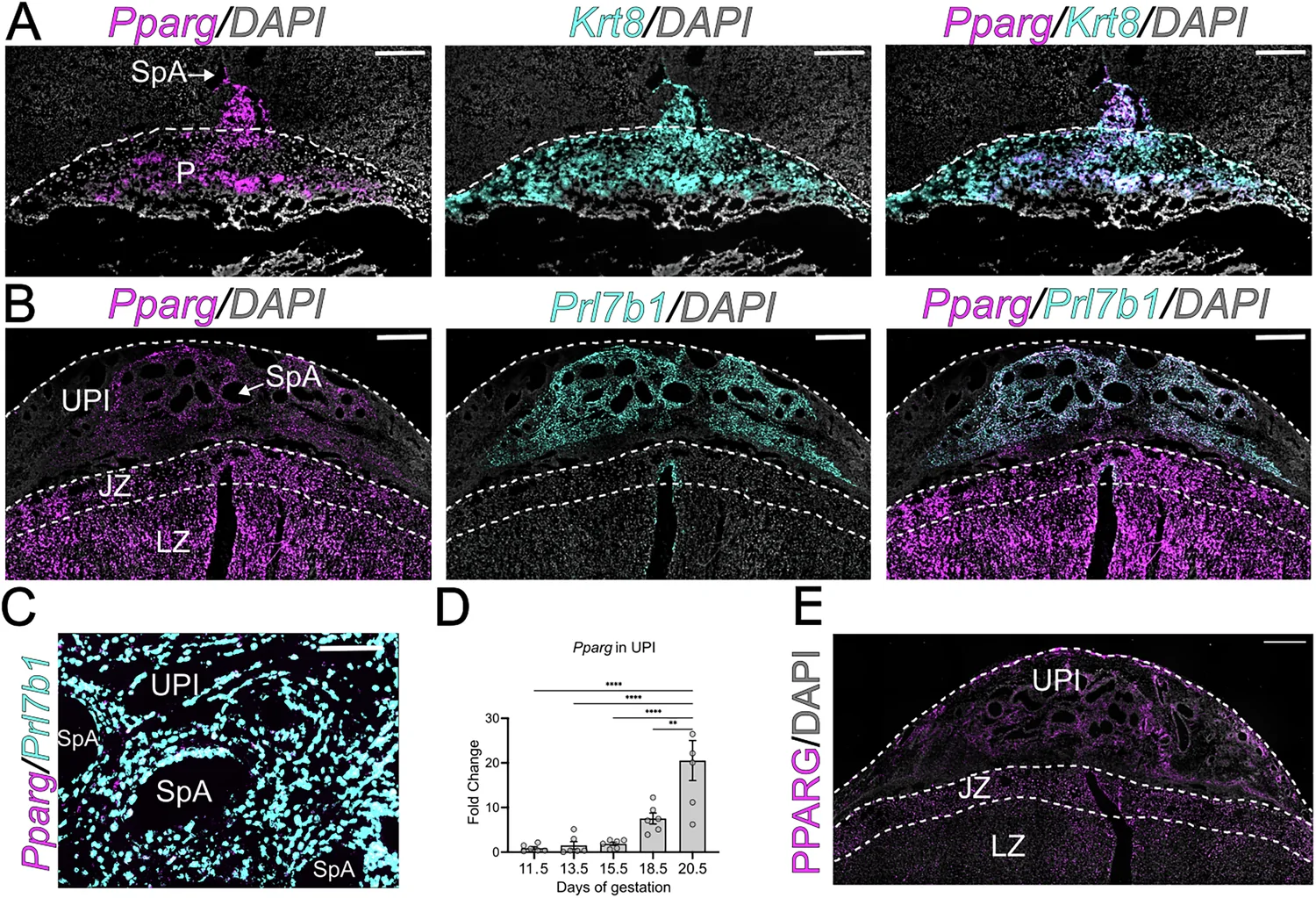
Distribution of *Pparg* transcripts and protein in rat placental tissues. *Pparg* transcripts were localized within rat placentation sites using in situ hybridization. (**A**) *Pparg* transcripts (magenta) were co-localized with *Krt8* transcripts (cyan) at gestation day (gd) 11.5 (*Pparg* and *Krt8* transcript co-localization: white). White dashed lines demarcate the placental-uterine boundary. (**B**) *Pparg* (magenta) and *Prl7b1* (cyan) transcripts within the gd 18.5 placentation site (*Pparg* and *Prl7b1* transcript co-localization: white). White dashed lines demarcate placental and uterine compartments. (**C**) Higher magnification of the placentation site surrounding the spiral arteries showing co-localization of *Pparg* and *Prl7b1* transcripts (white). (**D**) RT-qPCR measurement of *Pparg* transcript levels in uterine–placental interface tissue at gd 11.5, 13.5, 15.5, 18.5, and 20.5. Data were presented as mean ± standard error of the mean. Each data point represents a biological replicate from six independent pregnancies (gd 11.5, 13.5, or 15.5 versus gd 20.5, ****p < 0.0001; gd 18.5 versus gd 20.5 **p = 0.0019; *n* = 6 for each gd). Statistical analyses were performed with unpaired *t*-tests. (**E**) Immunohistochemical localization of PPARG protein within the gd 18.5 rat uterine–placental interface. White dashed lines demarcate placental and uterine compartments. Scale bars for panels **A**, **B**, **E** = 500 μm, and panel **C** = 50 μm. SpA spiral artery, UPI uterine–placental interface, P placenta, JZ junctional zone, LZ labyrinth zone. Source data are available online for this figure.

The rat exhibits deep intrauterine trophoblast cell invasion (Ain et al, 2003), a feature of placentation also observed in the human, and has proven to be a useful in vivo model for investigating the physiological relevance of genes implicated in the regulation of the invasive trophoblast cell lineage (Chakraborty et al, 2016; Varberg et al, 2021; Muto et al, 2021; Kuna et al, 2023; Dominguez et al, 2025; Moreno-Irusta et al, 2026b). We next sought to determine whether PPARG was expressed in trophoblast cells of the rat placenta, especially within invasive trophoblast cells of the rat uterine–placental interface. The rat placentation site is organized into three main compartments: (i) labyrinth zone, (ii) junctional zone, and (iii) uterine–placental interface (Soares et al, 2012; Shukla and Soares, 2022). The labyrinth zone is comprised of trophoblast cells and fetal vasculature and represents the site of maternal-fetal exchange, whereas the junctional zone contains several trophoblast cell types, including invasive trophoblast cell progenitors, which collectively direct pregnancy-dependent adaptations of maternal tissues. The uterine–placental interface is the uterine compartment adjacent to the placenta where invasive trophoblast cells, uterine vasculature, and maternal immune cells are situated. In situ hybridization was used to determine the distribution of *Pparg* transcripts. At gestation day (gd) 11.5, *Pparg* transcripts were localized to trophoblast cells of the developing placenta as well as to invasive trophoblast cells situated within uterine spiral arteries (Fig. 6A). The presence of *Pparg* transcripts in trophoblast cells was confirmed by co-localization with keratin 8 (*Krt8*), an epithelial cell-associated transcript expressed by trophoblast cells (Fig. 6A). By gd 18.5, *Pparg* was expressed within trophoblast cells of the labyrinth zone, junctional zone, and within invasive trophoblast cells of the uterine–placental interface (Fig. 6B). Expression of *Pparg* in invasive trophoblast cells was supported by its co-localization with prolactin family 7, subfamily b, member 1 (*Prl7b1*, Fig. 6B,C), an invasive trophoblast cell-associated transcript (Scott et al, 2022; Wiemers et al, 2003). These findings are consistent with a previously published report describing single-cell RNA-seq analysis of the rat uterine–placental interface, which demonstrated that *Pparg* expression was enriched in the invasive trophoblast cell lineage (Appendix Fig. S2, Scott et al, 2022). *Pparg* transcript levels increased within the uterine–placental interface as pregnancy progressed (Fig. 6D). Furthermore, PPARG protein was localized to invasive trophoblast cells and throughout the junctional and labyrinth zones, as shown by immunohistochemistry (Fig. 6E). Thus, PPARG expression is a conserved feature of EVT cells of the human placenta (Fig. 1) and invasive trophoblast cells of the rat placentation site (Fig. 6).

### Establishment and validation of the invasive trophoblast cell-specific Pparg mutant rat

**Figure 7:**
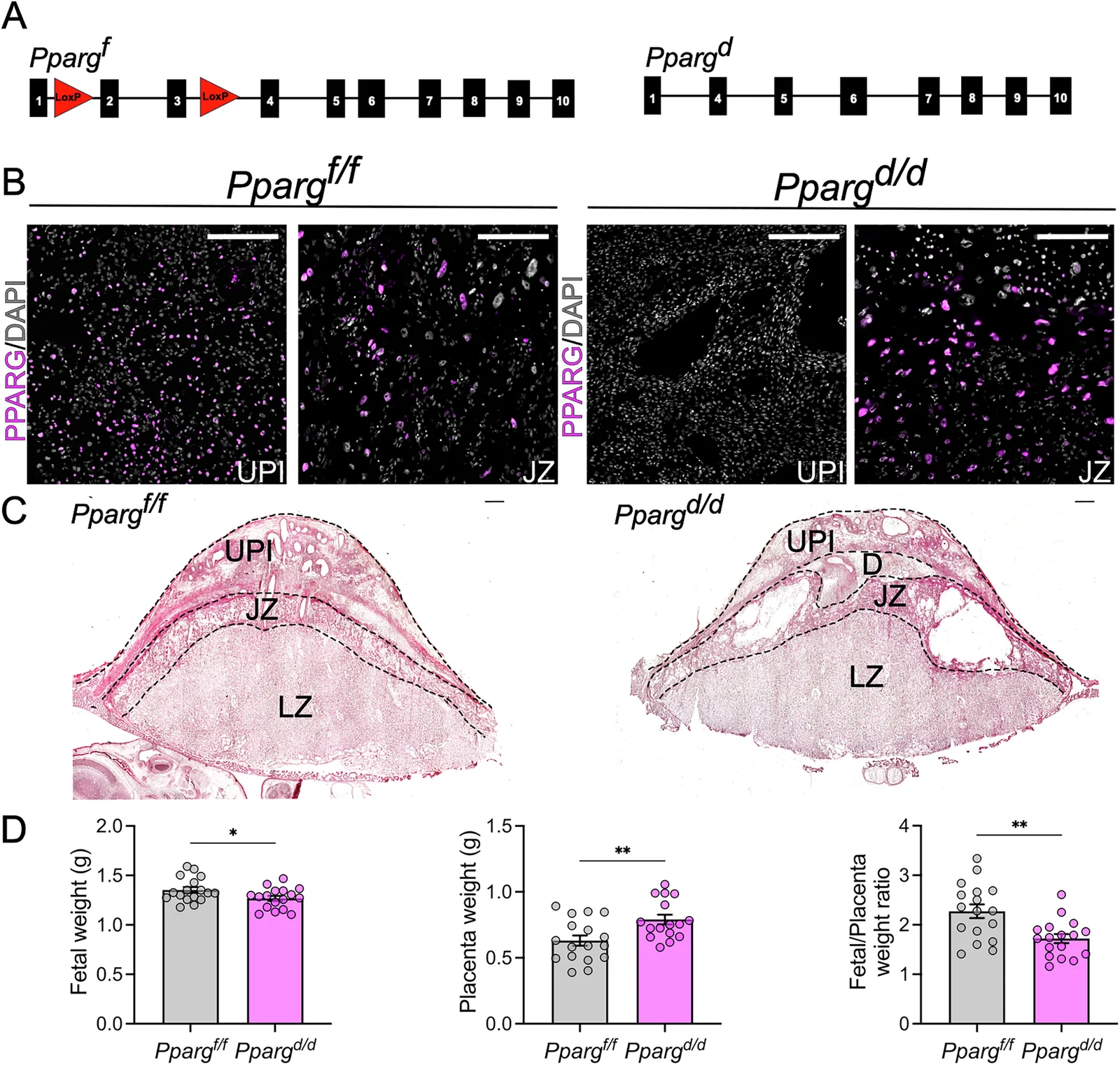
Rat invasive trophoblast-specific deletion of *Pparg.* (**A**) Schematic representation of loxP sites flanking Exons 2 and 3 of the *Pparg* gene and their conditional deletion following Cre recombinase activity driven by *Prl7b1* regulatory DNA. (**B**) PPARG immunostaining of *Pparg*^*f/f*^ and *Pparg*^*d/d*^ gestation day (gd) 18.5 placentation sites. Images show uterine–placental interface tissues (left) and junctional zone tissues (right) for each group, scale bar = 50 μm. (**C**) Hematoxylin and eosin staining of *Pparg*^*f/f*^ and *Pparg*^*d/d*^ gd 18.5 placentation sites, scale bar = 500 μm. (**D**) Fetal weight, placental weight, and fetal-to-placental weight ratio from gd 18.5 *Pparg*^*f/f*^ and *Pparg*^*d/d*^ conceptuses. Data were presented as mean ± standard error of the mean. Each data point represents a biological replicate from six pregnancies (*Pparg*^*f/f*^ versus *Pparg*^*d/d*^: Fetal weight, *p = 0.037; Placenta weight, **p = 0.0058, fetal/placenta weight ratio, **p = 0.0021; *n* = 17 for each genotype). Statistical analyses were performed using unpaired *t*-tests. SpA spiral artery, UPI uterine–placental interface, JZ junctional zone, LZ labyrinth zone, D decidua. Source data are available online for this figure.

Next, we examined an in vivo role for PPARG in the development of rat invasive trophoblast cells. Since PPARG is expressed in trophoblast cell lineages throughout the rat placentation site (Fig. 6B) and global inactivation of the mouse *Pparg* gene was associated with midgestation lethality (Barak et al, 1999; Kubota et al, 1999), we utilized a conditional Cre/Lox approach to selectively disrupt *Pparg* in the invasive trophoblast cell lineage. For these experiments, we used a rat model possessing Cre recombinase inserted into the *Prl7b1* locus. As noted above, *Prl7b1* is robustly and specifically expressed within invasive trophoblast cells of the rat placentation site (Wiemers et al, 2003; Scott et al, 2022; Iqbal et al, 2024). The *Prl7b1-Cre* specifically acts within the invasive trophoblast cell lineage without anomalous activities in non-invasive trophoblast cells or other extraembryonic and embryonic tissues (Dominguez et al, 2025; Iqbal et al, 2024). Male *Prl7b1-Cre* rats and female rats possessing *loxp* sites flanking Exons 2 and 3 of the *Pparg* gene (Fig. 7A) were used in the analysis. Specifically, *Prl7b1-Cre*/heterozygous floxed *Pparg* (*Prl7b1*^*Cre*^*/Pparg*^*f****/+***^) males were mated with homozygous floxed *Pparg* female rats (*Pparg*^*f****/****f*^) to generate *Pparg*^*f/f*^ and *Pparg* deleted (*Pparg*^*d/d*^) placentation sites. Immunohistochemical analysis for PPARG demonstrated the presence of PPARG protein at the gd 18.5 uterine–placental interface of *Pparg*^*f/f*^ but not within this compartment of *Pparg*^*d/d*^ placentation sites (Fig. 7B).

### PPARG involvement in rat invasive trophoblast cell lineage development and cellular dynamics within the uterine–placental interface

**Figure EV5:**
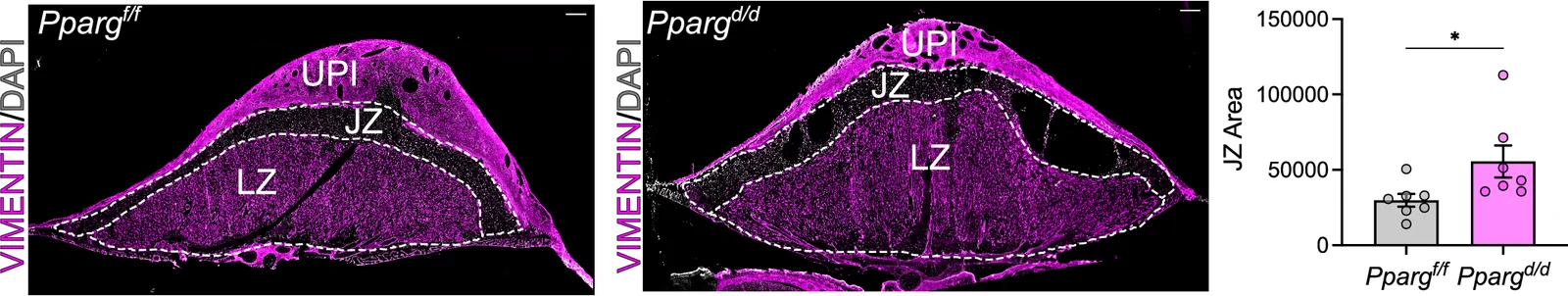
Junctional zone areas in *Pparg*^*f/f*^ and *Pparg*^*d/d*^ placentation sites. Left and central panels, Immunohistochemical staining for vimentin, which is positive in the labyrinth zone (LZ) and uterine–placental interface (UPI) and negative in the junctional zone (JZ). Right panel, quantification of JZ areas in *Pparg*^*f/f*^ and *Pparg*^*d/d*^ placentation sites. *Pparg*^*d/d*^ JZs are significantly larger than *Pparg*^*f/f*^ JZs (*p = 0.0442). Data were presented as mean ± standard error of the mean. Each data point represents a biological replicate from six independent pregnancies (*n* = 7). Statistical analyses were performed with an unpaired *t*-test. White dashed lines demarcate placental and uterine compartments.

The structure and integrity of *Pparg*^*f/f*^ and *Pparg*^*d/d*^ placentation sites were different. An expanded decidual compartment was evident within *Pparg*^*d/d*^ placentation sites (Fig. 7C). *Pparg*^*d/d*^ placentas were larger (Fig. 7C), possessed an enlarged junctional zone (Fig. EV5) containing prominent cyst-like structures (Fig. 7C), and were less efficient than *Pparg*^*f/f*^ placentas (Fig. 7D).

**Figure 8:**
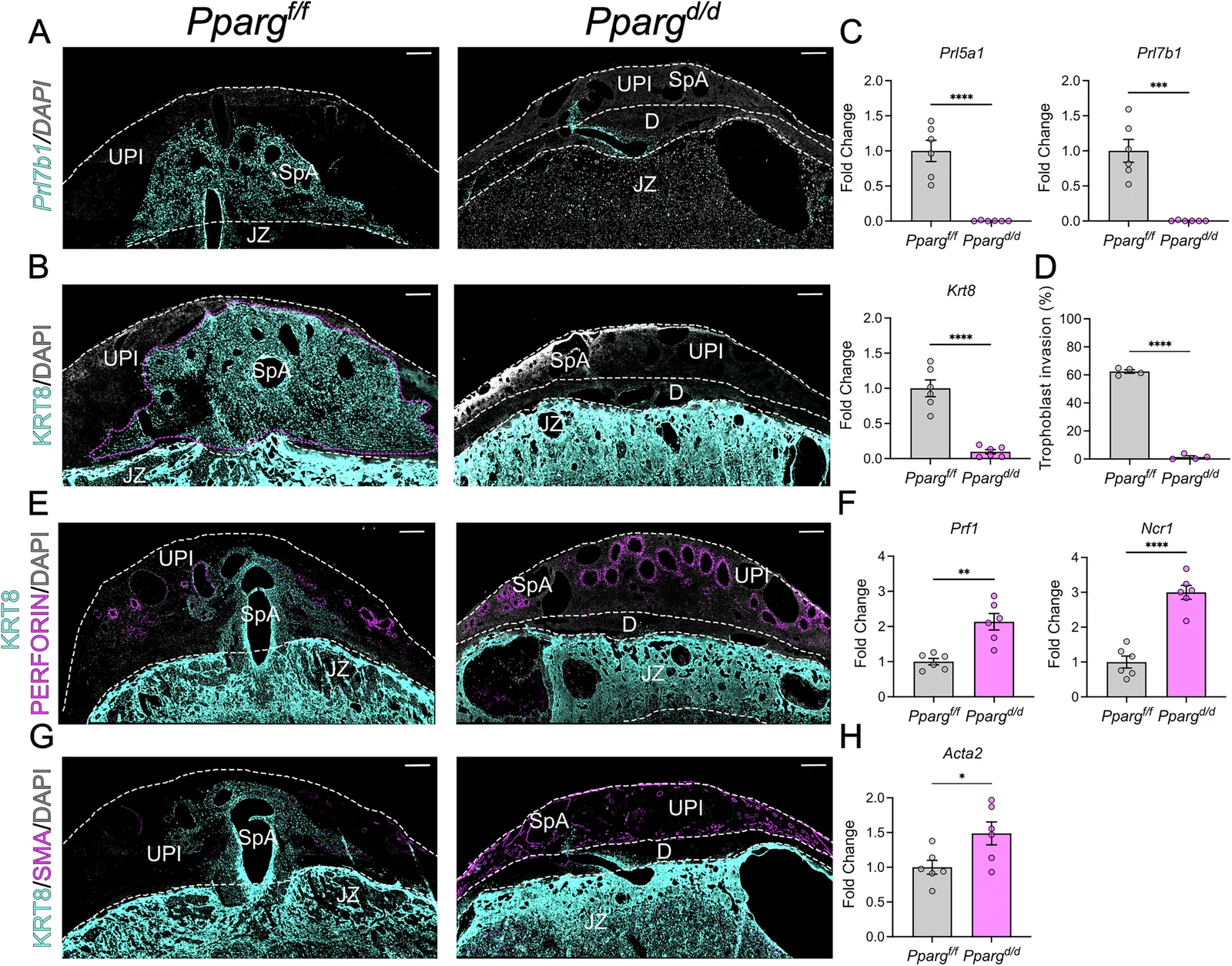
Invasive trophoblast cell-specific *Pparg* disruption impacts pregnancy-dependent transformation of the uterine–placental interface. (**A**) In situ hybridization showing *Prl7b1* transcripts (cyan) in gestation day (gd) 18.5 *Pparg*^*f/f*^ and *Pparg*^*d/d*^ placentation sites. White dashed lines demarcate placental and uterine compartments. (**B**) Immunohistochemical localization of KRT8 (cyan) in gd 18.5 *Pparg*^*f/f*^ and *Pparg*^*d/d*^ placentation sites. The magenta dashed line demarcates the area of trophoblast cell invasion. (**C**) RT-qPCR analysis of *Prl7b1, Prl5a1, and Krt8* expression in the uterine–placental interface of gd 18.5 *Pparg*^*f/f*^ and *Pparg*^*d/d*^ placentation sites (*Pparg*^*f/f*^ versus *Pparg*^*d/d*^: *Prl5a1*, ****p < 0.0001; *Prl7b1*, ***p = 0.0001; *Krt8*, ****p < 0.0001; *n* = 6). (**D**) Quantification of trophoblast cell invasion area (*Pparg*^*f/f*^ versus *Pparg*^*d/d*^: ****p < 0.0001, *n* = 4). (**E**) Immunohistochemical localization of perforin (PRF1), a natural killer (NK) cell-associated protein, (magenta) and KRT8 (cyan). White dashed lines demarcate the placental and uterine compartments. (**F**) RT-qPCR analysis of NK cell-associated transcripts (*Prf1* and *Ncr1*) in the uterine–placental interface of gd 18.5 *Pparg*^*f/f*^ and *Pparg*^*d/d*^ placentation sites (*Pparg*^*f/f*^ versus *Pparg*^*d/d*^: *Prf1*, **p = 0.0011; *Ncr1*, ****p < 0.0001; *n* = 6). (**G**) Immunohistochemical localization of smooth muscle actin (SMA, magenta) and KRT8 (cyan) in the uterine–placental interface of gd 18.5 *Pparg*^*f/f*^ and *Pparg*^*d/d*^ placentation sites. White dashed lines demarcate the placental and uterine compartments. (**H**) RT-qPCR analysis of *Acta2* transcript in the uterine–placental interface of gd 18.5 *Pparg*^*f/f*^ and *Pparg*^*d/d*^ placentation sites (*Pparg*^*f/f*^ versus *Pparg*^*d/d*^: *Acta2*, *p = 0.0294, *n* = 6). Scale bars for images in panels (**A**, **B**, **E**, **G**) = 500 μm. Data were presented as mean ± standard error of the mean. Each data point represents a distinct uterine–placental interface sample obtained from four to six pregnancies. Statistical analyses were performed using unpaired *t*-tests. UPI uterine–placental interface, JZ junctional zone, LZ labyrinth zone, D decidua, SpA spiral artery. Source data are available online for this figure.

Invasive trophoblast cells arise from the junctional zone (Soares et al, 2012; Shukla and Soares, 2022) and exhibited pronounced differences between *Pparg*^*f/f*^ and *Pparg*^*d/d*^ placentation sites (Fig. 8).

The uterine–placental interface of *Pparg*^*f/f*^ placentation sites was infiltrated with invasive trophoblast cells, which was not observed in *Pparg*^*d/d*^ placentation sites, as detected by in situ hybridization for *Prl7b1* or pan-cytokeratin immunostaining (Fig. 8A,B). Deficits in intrauterine trophoblast cell invasion correlated with diminished expression of invasive trophoblast cell-associated transcripts (*Prl5a1*, *Prl7b1*, *Krt8, Krt7, Cited2, Peg3, and Tfpi*) and pan-cytokeratin immunoreactive surface area within the uterine–placental interfaces of *Pparg*^*d/d*^ placentation sites (Fig. 8C,D; Appendix Fig. S3). Previously published single-cell RNA-sequencing and single nucleus assay for transposase-accessible chromatin-sequencing datasets [(Scott et al, 2022; Data ref: Scott et al, 2022; Vu et al, 2023; Data ref: Vu et al, 2023)] were used to reveal a network of genes potentially regulated by PPARG in rat invasive trophoblast cells. These putative PPARG targets include candidate contributors to remodeling the extracellular matrix, regulation of cell movement, cytoskeletal restructuring, and transcriptional regulators that act on genes encoding proteins affecting the invasive trophoblast cell phenotype (Appendix Table S1; Dataset EV9).

Diminished intrauterine trophoblast cell invasion in *Pparg*^*d/d*^ placentation sites was accompanied by a retention of natural killer (NK) cells within the uterine–placental interface (Fig. 8E) and increased expression of NK cell-associated transcripts (Fig. 8F). NK cells are known contributors to uterine spiral artery remodeling (Chakraborty et al, 2011; Rätsep et al, 2015; Renaud et al, 2017; Erlebacher, 2013) but were less effective than invasive trophoblast cells in disabling smooth muscle cells associated with uterine spiral arteries (Fig. 8G,H).

Overall, these results highlight a key role for PPARG in directing development of the invasive trophoblast cell lineage and the important role that invasive trophoblast cells possess in regulating the cell composition and function of the uterine–placental interface.

## Discussion

Hemochorial placentation serves the important function of delivering nutrients to ensure coordinated on-time growth and development of the fetus within the female reproductive tract (Soares et al, 2018). This type of placentation is found in many primates and rodents, including the human and rat (Carter and Enders, 2004; Roberts et al, 2016). These two species exhibit extensive invasive trophoblast cell-guided uterine transformation, including restructuring of spiral arteries, which is fundamental to the acquisition of maternal resources required for fetal development (Pijnenborg et al, 1981). We examined a role for PPARG in the development of invasive trophoblast cells using human TS cells and a genetically manipulated rat model. In human placentation, PPARG was expressed in EVT cell column cells and was shown to coordinate the upregulation of genes required for cell elongation and the process of EVT cell movement. In the rat, PPARG was expressed throughout the placenta, including invasive trophoblast cells, and was essential for the development of the invasive trophoblast cell lineage. Failures in PPARG-mediated gene regulation also affected human TS cell proliferation and led to abnormalities within compartments of the rat placenta. The experimentation demonstrated that PPARG is a conserved regulator of invasive trophoblast cell development and function.

Earlier research connected PPARG to placentation (Schaiff et al, 2006; Fournier et al, 2011; Kadam et al, 2015). Insights regarding PPARG actions in placentation were unequivocally demonstrated from analysis of global PPARG null mouse models (Barak et al, 1999; Kubota et al, 1999). PPARG disruption led to midgestation lethality due to an impairment of early events in placental morphogenesis (Barak et al, 1999; Kubota et al, 1999). Considerable effort has also been directed towards analyses of roles for PPARG in regulating trophoblast cells using an assortment of in vitro models. PPARG-deficient mouse TS cells exhibit impairments in proliferation and syncytiotrophoblast development and enhanced trophoblast giant cell differentiation (Parast et al, 2009), whereas human cytotrophoblast exposure to PPARG agonists promoted syncytiotrophoblast formation and trophoblast cell-lipid accumulation, while inhibiting trophoblast cell invasive properties (Tarrade, 2001; Tarrade et al, 2001; Schaiff et al, 2005). Our in vitro and in vivo experimentation supported an essential role for PPARG in EVT/invasive trophoblast cell lineage development. Similarly, using human TS cells, Guo and coworkers recently demonstrated the involvement of PPARG in EVT cell differentiation (Guo et al, 2025). Exposure to a PPARG antagonist supported the involvement of PPARG actions in promoting EVT cell differentiation. These PPARG actions on the EVT/invasive trophoblast cell lineage differ from observations with PPARG-activating ligands. PPARG can act independently of ligand, and its actions can vary depending on the ligand investigated and may differ depending on the developmental state of the target cells examined (Sauer, 2015). Thus, PPARG is a broad-spectrum regulator of placental biology with critical actions on the development of the EVT/invasive trophoblast cell lineage.

As indicated above, PPARG is expressed in multiple trophoblast cell lineages (Barak et al, 1999; Tarrade, 2001; Fournier et al, 2007; Waite et al, 2005; Asami-Miyagishi et al, 2004). Each trophoblast cell lineage is responsible for controlling tasks critical for the development and function of the placenta (Soares et al, 2018). Context is important. PPARG actions are not only determined by its presence but also by the complement of posttranslational modifiers, heterodimerization binding partners, and co-regulators expressed by a cell (Berger and Moller, 2002; Feige et al, 2006; Ahmadian et al, 2013).

In addition to PPARG, several other proteins have been shown to contribute to the regulation of EVT cell differentiation. ADAMTS20, ASCL2, CCNB1, CEBPB, CITED2, DLX5, DLX6, EPAS1, GCM1, NOTUM, NRIP1, SNAI1, TFAP2C, TFPI, WWTR1, and ZNF439, are required for optimal transition from the TS cell stem state to EVT cells (Varberg et al, 2023; Shukla et al, 2024; Varberg et al, 2021; Muto et al, 2021; Kuna et al, 2023; Kim et al, 2024; Jeyarajah et al, 2022; Meinhardt et al, 2025; Moreno-Irusta et al, 2026a). A *CRISPR* screen identified additional transcription factors contributing to the regulation of human TS cells (Shimizu et al, 2023). The screen demonstrated the involvement of PPARG in the control of TS cell proliferation, which was consistent with our observations, but not EVT cell differentiation. The *CRISPR* screen utilized HLA-G expression as a method for monitoring EVT cell differentiation. PPARG does not significantly affect HLA-G gene expression, which may account for differences in the outcome of the *CRISPR* screen versus our findings. Based on RNA-seq and ChIP-seq experiments, we can place PPARG in a potential hierarchy of transcription factors controlling EVT cell development (Appendix Fig. S4, Varberg et al, 2023, 2021; Kuna et al, 2023; Kim et al, 2024; Jeyarajah et al, 2022; Guo et al, 2025). Among the regulators impacting EVT cell differentiation, ASCL2, TFPI, CITED2, TFAP2C, and PPARG possess conserved actions promoting development of the invasive trophoblast cell lineage in the rat (Varberg et al, 2021; Muto et al, 2021; Kuna et al, 2023; Dominguez et al, 2025).

PPARG deficiency was associated with failed trophoblast cell invasion into the uterus, leading to a dramatic reorganization of the rat placentation site. The reorganization included the retention of NK cells within the uterine–placental interface, expanded decidual tissue, and a disruption in the structural organization of the two main placental compartments (junctional and labyrinth zones). NK cells are known contributors to early events in uterine spiral artery remodeling (Chakraborty et al, 2011; Erlebacher, 2013; Rätsep et al, 2015; Renaud et al, 2017). NK cells vacate the uterine–placental interface as invasive trophoblast cells enter the uterus (Ain et al, 2003). In addition to PPARG, TFAP2C and PLAC1 are also drivers of invasive trophoblast cell infiltration into the uterus (Dominguez et al, 2025; Moreno-Irusta et al, 2026b). Impairments of PPARG, TFAP2C, or PLAC1 lead to failed intrauterine trophoblast cell invasion and retention of NK cells within the uterine–placental interface. The implication is that invasive trophoblast cells are engineering the exit of NK cells from the uterine–placental interface. In the absence of invasive trophoblast cells, NK cells are supporting, at least partially, uterine spiral artery remodeling and minimize adverse pregnancy outcomes. The nature of the signal(s) emanating from invasive trophoblast cells responsible for NK cell exodus is unknown. Cooperation of invasive trophoblast cells and NK cells at the uterine–placental interface is robust. It is expected that deficits in both maternal and extraembryonic contributions to uterine transformation would exhibit more adverse consequences. Expansion of the decidua compartment in *Pparg*^*d/d*^ placentation sites could also be contributing to decreased trophoblast cell migration towards the uterine–placental interface within the *Pparg*^*d/d*^ placentation sites. In addition to the uterine–placental interface, failure in the developmental progression of progenitor cells situated in the junctional zone into invasive trophoblast cells resulted in malformations within the junctional zone. These aberrations included the appearance of large cystic structures within the junctional zone and an irregular junctional zone-labyrinth zone interface. Specific cellular dysfunctions linked to PPARG deficiency underlying these structural defects in junctional zone morphogenesis are unknown.

Finally, PPARG transcriptional activity is influenced by the availability of glucose and lipids (Berger and Moller, 2002; Feige et al, 2006; Ahmadian et al, 2013). Thus, PPARG acts as a nutrient sensor. Coupling nutrient sensing with the regulation of invasive trophoblast cell transformation of the uterine vasculature would be an effective strategy for adapting to a nutrient-variable maternal environment and is testable with the in vitro and in vivo models described in this report.

## Methods

**Table:** Reagents and tools table

Reagent/resource	Reference or source	Identifier or catalog number
**Experimental models**		
Holtzman SD rat	Envigo (Inotiv)	003
Human trophoblast stem cells	(Okae et al, 2018)	CT27 (X;X) and CT29 (X;Y)
**Recombinant DNA**		
Lenti-X cells	Takara Bio USA	632180
pMDLg/pRRE plasmid	Addgene	12251
pRSV-Rev plasmid	Addgene	12253
pMD2.G plasmid	Addgene	12259
**Antibodies**		
phycoerythrin (**PE**)-labeled anti-HLA-G antibody	Abcam	ab24384
Vimentin antibody	Santa Cruz Biotechnology	sc-6260
Pan-cytokeratin antibody	Sigma-Aldrich	F3418
PPARG antibody	Santa Cruz Biotechnology	sc-7273
PPARG antibody	Cell Signaling Technology	2435S
Alexa Fluor 488-conjugated goat anti-mouse IgG antibody	Thermo Fisher	A11001
Alexa Fluor 568-conjugated goat anti-rabbit IgG antibody	Thermo Fisher	A1101
CGB antibody	Abcam	ab9528
TP63 antibody	Abcam	ab124762
GAPDH antibody	Invitrogen	AM4300
Anti-mouse immunoglobulin G conjugated to horseradish peroxidase	Cell Signaling Technology	7076
Anti-rabbit immunoglobulin G conjugated to horseradish peroxidase	Cell Signaling Technology	5127
**Oligonucleotides and other sequence-based reagents**		
PPARG and Control shRNA sequences	This study	Appendix Table S2
Guide RNA and template for LoxP Ppparg gene	This study	Appendix Table S3
Primers for genotyping and sex determination	This study	Appendix Table S4
RT-qPCR human primer list	This study	Appendix Table S5
RT-qPCR rat primer list	This studay	Appendix Table S6
human *PPARG* probe	Advanced Cell Diagnostics	441691
human *NOTUM* probe	Advanced Cell Diagnostics	430311
rat *Pparg* probe	Advanced Cell Diagnostics	313251
rat *Krt8* probe	Advanced Cell Diagnostics	87304-C2
rat *Prl7b1* probe	Advanced Cell Diagnostics	860181-C2
**Chemicals, enzymes and other reagents**		
Attractene	Qiagen	301005
Opti-MEM I	Thermo Fisher	51985-034
DMEM	Thermo Fisher	11995-065
FBS	Thermo Fisher	16141-061
collagen IV	Thermo Fisher	CB40233
polybrene	Sigma-Aldrich	R-1003-50UL
puromycin dihydrochloride	Thermo Fisher	A11138-03
DMEM/F12	Thermo Fisher	11320033
2-mercaptoethanol	Sigma-Aldrich	M6250
Penicillin/Streptomycin	Gibco	15-140-122
BSA	Thermo Fisher	BP9704100
ITS-X	Gibco	51-500-056
L-ascorbic acid	Sigma-Aldrich	A8960
**EGF**	Sigma-Aldrich	E9644
CHIR99021	Reprocell	04-0004
A83-01	Reprocell	04-0014
SB431542	Reprocell)	04-0010
valproic acid	Sigma-Aldrich	P4543
Y27632	Reprocell	04-0012-02
neuregulin 1 (**NRG**1)	Cell Signaling	5218SC
KnockOut Serum Replacement (KSR)	Thermo Fisher	10828028
Matrigel®	Thermo Fisher	CB-40234
GW9662	Cayman Chemical	70785
N2 1X	Thermo Fisher	17502-048
B27	Thermo Fisher	17504044
Primocin	Invitrogen	ant-pm-1
N-acetyl-L-cysteine	Sigma-Aldrich	A9165
L-glutamine	Gibco	25030081
hepatic growth factor (**HGF**)	Peprotech	100-39
R-Spondin 1	BioTechne	4645-RS
Fibroblast growth factor-2 (**FGF2**)	Peprotech	100-18B
prostaglandin E2	Sigma-Aldrich	P0409
TrypLE	Thermo Fisher	12604021
paraformaldehyde	Sigma-Aldrich	P6148-500G
crystal violet	Fisher Scientific	C581-100
4’,6-diamidino-2-phenylindole (**DAPI**)	Invitrogen	D1306
TRIzol	Thermo Fisher	15596018
High-Capacity Reverse Transcription Kit	Applied Biosystems	4368814
PowerSYBR Green PCR Master Mix	Thermo Fisher	4367659
RNAscope® Multiplex Fluorescent Reagent Kit v2	Advanced Cell Diagnostics	323100
4’,6-diamidino-2-phenylindole (**DAPI**)	Advanced Cell Diagnostics	323108
Goat serum	Thermo Fisher	50062Z
Fluoromount-G	Southern Biotech	0100-01
Cell Recovery Solution	Corning	354253
Radioimmunoprecipitation assay lysis buffer	Santa Cruz Biotechnology	sc-24948A
DC Protein Assay Kit	Bio-Rad	5000112
Polyvinylidene difluoride membranes	GE Healthcare	10600023
Immobilon Crescendo Western HRP Substrate	Sigma-Aldrich	WBLUR0500
SimpleChIP^®^ Enzymatic Chromatin Immunoprecipitation Kit (Magnetic Beads)	Cell Signaling Technology	9003
**Software**		
ImageJ software		
GraphPad Prism 10.2.3 software		
**Other**		

### Human placentation site specimens

Paraffin-embedded first-trimester placental tissue specimens were obtained from the Lunenfeld-Tanenbaum Research Institute at Mount Sinai Hospital (Toronto, Canada). All tissue samples were deidentified prior to use in this study. Tissue collection was conducted following written informed consent and with approval from the Human Research Ethics Review Committees at the University of Toronto and the University of Kansas Medical Center (KUMC); approval number 12-0051-E.

### Human TS cell culture

Human TS cells (CT27, X,X and CT29, X,Y) used in this study have been previously described (Okae et al, 2018). The TS cells originated from deidentified first-trimester human placental tissue obtained from healthy women with signed informed consent and approval from the Ethics Committee of Tohoku University School of Medicine (approval number 2014-1-879). Experimentation with human TS cells was approved by the KUMC Human Research Protection Program and the KUMC Human Stem Cell Research Oversight Committee (approval number 2024-001). Human TS cells were cultured in 100 mm tissue culture dishes coated with 5 μg/mL collagen IV (CB40233, Thermo Fisher). Complete Human TS Cell Medium was used to maintain cells in the stem state [DMEM/F12 (11320033, Thermo Fisher), 100 μM 2-mercaptoethanol, 0.2% vol/vol (M6250, Sigma-Aldrich), fetal bovine serum (FBS, 16141-061, Thermo Fisher), 50 U/ml penicillin, 50 μg/mL streptomycin (15-140-122, Gibco), 0.3% bovine serum albumin (BSA, BP9704100, Thermo Fisher), 1% insulin-transferrin-selenium-ethanolamine (ITS-X, solution, vol/vol, 51-500-056, Gibco), 1.5 μg/mL L-ascorbic acid (A8960, Sigma-Aldrich), 50 ng/mL epidermal growth factor (EGF, E9644, Sigma-Aldrich), 2 μM CHIR99021 (04-0004, Reprocell), 0.5 μM A83-01 (04-0014, Reprocell), 1 μM SB431542 (04-0010, Reprocell), 0.8 mM valproic acid (P4543, Sigma-Aldrich), and 5 μM Y27632 (04-0012-02, Reprocell)].

To induce EVT cell differentiation, TS cells were plated in 6-well plates pre-coated with 1 μg/mL collagen IV at a density of 60,000 cells per well. Cells were cultured in EVT Cell Differentiation Medium [DMEM/F12, 100 μM 2-mercaptoethanol, 50 U/ml penicillin, 50 μg/mL streptomycin, 0.3% BSA, 1% ITS-X solution, 100 ng/mL of neuregulin 1 (NRG1, 5218SC, Cell Signaling), 7.5 μM A83-01, 2.5 μM Y27632, 4% KnockOut Serum Replacement (KSR, 10828028, Thermo Fisher), and 2% Matrigel® (CB-40234, Thermo Fisher)]. On day 3 of EVT cell differentiation, the medium was replaced with EVT Cell Differentiation Medium excluding NRG1 and with a reduced Matrigel® concentration of 0.5%. On day 6 of EVT cell differentiation, the medium was replaced with EVT Cell Differentiation medium excluding NRG1 and KSR, and with a Matrigel® concentration of 0.5%.

Effects of a PPARG antagonist, GW9662 10 μM (70785, Cayman Chemical), on EVT cell differentiation were also examined.

### Design and generation of lentiviral shRNA constructs

PPARG shRNA sequences were designed using the Genetic Perturbation Platform web portal from the Broad Institute (https://portals.broadinstitute.org/gpp/public/analysis-tools/sgrna-design) and subcloned into the pLKO.1 vector at the *AgeI* and *EcoRI* restriction sites. An shRNA control (shCTRL, Plasmid 1864, Addgene), which does not recognize any known mammalian gene, was similarly subcloned into the pLKO.1 vector and used as a control. The shRNA sequences used in these analyses are listed in Appendix Table S2. Lentiviral packaging plasmids were obtained from Addgene and included pMDLg/pRRE (plasmid 12251), pRSV-Rev (plasmid 12253), and pMD2.G (plasmid 12259). Lentiviral particles were produced via transient transfection of the shRNA-pLKO.1 vector along with the packaging plasmids into Lenti-X cells (632180, Takara Bio USA) using Attractene (301005, Qiagen) in Opti-MEM I (51985-034, Thermo Fisher). Cells were maintained in Dulbecco’s Modified Eagle Medium (DMEM, 11995-065, Thermo Fisher) supplemented with 10% FBS for 24 h prior to supernatant collection. At that point, cells were switched to Basal Human TS Cell Medium. Conditioned medium containing viral particles was collected every 24 h over 2 days and stored at −80 °C until used.

### Lentiviral transduction

Human TS cells were plated at 65,000 cells per well in 6-well tissue culture-treated plates coated with 5 μg/mL collagen IV (CB40233, Thermo Fisher) and incubated for 24 h. Prior to transduction, the medium was changed, and cells were incubated with 2.5 μg/mL polybrene for 30 min at 37 °C. Following polybrene treatment, TS cells were transduced with 500 μL of conditioned medium containing lentiviral particles and then incubated for 24 h. Medium was changed at 24 h post-transduction and selected with puromycin dihydrochloride (5 μg/mL, A11138-03, Thermo Fisher) for 2 days. Cells recovered were cultured for 1 to 3 days in Complete TS Cell Medium and then used to evaluate the effects of PPARG disruption on EVT cell differentiation.

### Cell proliferation assay

Cells were seeded in six-well plates at a density of 50,0000 cells/well and allowed to adhere and grow for 5 days in Complete Human TS Cell Medium at 37 °C with 5% CO₂. At the indicated time point, cells were fixed with 4% paraformaldehyde for 15 min at room temperature, then washed twice with phosphate-buffered saline (PBS, pH 7.4). Fixed cells were stained with 0.5% crystal violet (C581-100, Fisher Scientific) solution (in 20% methanol) for 20 min. Excess stain was removed by rinsing the wells gently with tap water until clear. Plates were air-dried. Stained cells were solubilized with 100% methanol, and absorbance was measured at 570 nm using a microplate reader. Absorbance values were normalized to control samples to assess relative cell proliferation.

### Cell migration assay

Trophoblast cell migration was assessed according to the method described by Shimizu and coworkers (Shimizu et al, 2023) with minor modifications. A suspension of 200,000 human TS cells was prepared in Matrigel (100%) and EVT Cell Differentiation Medium (10 μL). From this mixture, 1 μL was dispensed onto a six-well plate to form individual Matrigel drops. Six Matrigel drops were prepared. The drops were cultured in EVT Cell Differentiation Medium supplemented with 0.5% Matrigel (CB-40234, Thermo Fisher) at 37 °C in a humidified atmosphere containing 5% CO₂. The culture medium was replaced on day 3, and the cells were incubated for a total of five days. EVT cells migrating from the cell aggregate were identified using immunocytochemistry for HLA-G [1:300, phycoerythrin (PE)-labeled anti-HLA-G antibody, ab24384, Abcam]. After 15 min of incubation at 37 °C, cells were fixed with 4% paraformaldehyde (PFA). Nuclei were counterstained with 4’,6-diamidino-2-phenylindole (DAPI, 1:50,000, D1306, Invitrogen). Following washes with PBS, the samples were imaged using a Zeiss Axio Observer 7 microscope. The area of the PE-labeled signal was quantified using ImageJ software.

### Trophoblast organoids

Human TS cells were used to establish trophoblast organoids as previously described (Karvas et al, 2022) with minor modifications. The effects of PPARG on trophoblast organoid formation and capacity to differentiate into EVT cells were determined. Human TS cells (3000) were embedded in 30 μL of Matrigel (final concentration 72%) and plated as droplets in 24-well plates. After a 2-min incubation and 30-min polymerization at 37 °C, 500 μL of trophoblast organoid medium (TOM) was added. TOM consisted of Advanced DMEM/F12 (12-634-010, Thermo Fisher) supplemented with N2 1X (17502-048, Thermo Fisher), B27 1X (17504044, Thermo Fisher), 100 μg/mL Primocin (ant-pm-1, Invitrogen), 1.25 mM *N*-acetyl-L-cysteine (A9165, Sigma-Aldrich), 2 mM L-glutamine (25030081, Gibco), 500 nM A8301, 1.5 μM CHIR99021, 2 μM Y-27632, 50 ng/mL EGF, 50 ng/mL hepatocyte growth factor (HGF, 100-39, Peprotech), 80 ng/mL R-Spondin 1 (4645-RS, BioTechne), 100 ng/mL fibroblast growth factor-2 (FGF2, 100-18B Peprotech), and 2.5 μM prostaglandin E2 (P0409, Sigma-Aldrich). Medium was changed daily, and organoids were passaged every 8–10 days. Matrigel droplets were digested with TrypLE for 20 min at 37 °C, followed by three washes in Advanced DMEM/F12. Large aggregates were removed with a 40-μm filter, and 3000–5000 cells were reseeded per 30 μL Matrigel droplet.

To induce EVT cell differentiation, dissociated trophoblast organoid cells were embedded in 30 μL Matrigel droplets in 24-well plates and maintained in TOM for 3 days. The culture medium was then replaced with culture medium consisting of Advanced DMEM/F12 supplemented with 0.1 mM 2-mercaptoethanol, 0.5% penicillin/streptomycin, 0.3% bovine serum albumin, 1% ITS-X, 100 ng/mL NRG1, 7.5 μM A83-01, and 4% KSR for 5 days with daily culture medium replacement (500 μL per well). EVT cells were analyzed following 2 additional days of culture in the culture medium without NRG1.

### Animals and tissue collection

Holtzman Sprague–Dawley rats were obtained from Envigo and used for in vivo experiments. Animals were housed in an environmentally controlled facility under a 14-h light/10-h dark lighting schedule with food and water available ad libitum. Timed pregnancies were established by mating virgin female rats (8–10 weeks of age) with adult male rats (>3 months of age). Mating was confirmed by the presence of sperm in the vaginal lavage. The day of sperm detection was defined as gd 0.5. Pseudopregnant females were generated by mating with vasectomized males. The presence of a seminal plug was designated as day 0.5 of pseudopregnancy. Pregnant rats were euthanized on different days during gestation. Some placentation sites were frozen intact in dry ice–cooled heptane and stored at –80 °C for subsequent histological analysis, while other placentation sites were dissected into uterine–placental interface, junctional zone, and labyrinth zone compartments, as previously described (Ain et al, 2006).

### Conditional Pparg mutant rat

In vivo *Pparg* disruption in invasive trophoblast cells was achieved using the Cre-loxP system. LoxP cassettes were inserted into introns flanking Exons 2 and 3 of the *Pparg* locus using *CRISPR*/Cas9-mediated homologous recombination (Fig. 7; Appendix Table S3). *CRISPR*/Cas9 reagents and templates were electroporated into one-cell rat embryos using a NEPA21 electroporator (Nepa Gene), and embryos were subsequently transferred to pseudopregnant rats. Offspring were screened for mutations via polymerase chain reaction (PCR), and successful loxP site insertion was confirmed by DNA sequencing (GeneWiz). A founder mutant animal was backcrossed with wild-type rats to assess germline transmission. A *Prl7b1-Cre* recombinase rat model was used to specifically disrupt *Pparg* in invasive trophoblast cells (Iqbal et al, 2024).

Genotyping and sex chromosome determinations were performed as previously described (Dhakal and Soares, 2017). Genomic DNA was extracted from tail-tip biopsies using the Red Extract-N-Amp Tissue PCR Kit (XNAT-1000RXN, Sigma-Aldrich) and used for genotyping. Primer sequences for detecting conditional mutations and determining sex chromosome composition are provided in Appendix Table S4.

### RT-qPCR

Total RNA was isolated from tissues using TRIzol reagent (15596018, Thermo Fisher). One μg of RNA was used to synthesize complementary DNA (cDNA) using the High-Capacity Reverse Transcription Kit (4368814, Applied Biosystems), followed by a 1:10 dilution in nuclease-free water. Quantitative PCR (qPCR) was performed using PowerSYBR Green PCR Master Mix (4367659, Thermo Fisher) and transcript-specific primer sets (250 nM). Primer sequences are provided in Appendix Tables S5 and S6. qPCR was conducted with a QuantStudio 5 Real-Time PCR System (Thermo Fisher) using the following cycling conditions: an initial denaturation step at 95 °C for 10 min, followed by 40 cycles of two-step PCR (95 °C for 15 s, 60 °C for 1 min). A dissociation curve was then generated with the following steps: 95 °C for 15 s, 60 °C for 1 min, and a final step at 95 °C for 15 s. Relative mRNA expression levels were calculated using the ΔΔCt method. RNA polymerase II subunit A (*POLR2A*) and glyceraldehyde 3-phosphate dehydrogenase (*Gapdh*) were used as reference genes for human and rat samples, respectively.

### In situ hybridization

In situ hybridization was performed on paraffin-embedded human placenta and frozen rat placentation site tissue sections with the RNAscope® Multiplex Fluorescent Reagent Kit v2 (Advanced Cell Diagnostics) according to the manufacturer’s instructions. Probes were prepared by Advanced Cell Diagnostics to detect the following transcripts: human *PPARG* (441691, NM_138712.3, target region: 699–1779), human *NOTUM* (430311, NM_178493.5, target region: 259–814), rat *Pparg* (313251, NM_001145367.1, target region: 305–1932); rat *Krt8* (87304-C2, NM_199370.1, target region: 134–1472), and rat *Prl7b1* (860181-C2, NM_153738.1, target region: 28–900). Nuclei were visualized using DAPI (323108, Advanced Cell Diagnostics). Images were captured using a Zeiss Axio Observer 7 microscope.

### Immunohistochemistry of placental tissues

Formalin-fixed paraffin-embedded human placental tissue sections and frozen rat placentation site sections (10 μm thick) fixed in 4% paraformaldehyde were processed for immunohistochemical analyses. Tissue sections were blocked with 10% goat serum (50062Z, Thermo Fisher) and incubated overnight at 4 °C with primary antibodies against vimentin (1:300, sc-6260, Santa Cruz Biotechnology), pan-cytokeratin (1:100, F3418, Sigma-Aldrich), perforin (1:300, TP251, Amsbio), or PPARG (human: 1:100, sc-7273, Santa Cruz Biotechnology; rat: 1:100, 2435S, Cell Signaling Technology). Immunostaining was visualized using fluorescence-tagged secondary antibodies: Alexa Fluor 488-conjugated goat anti-mouse IgG (A11001, Thermo Fisher) and Alexa Fluor 568-conjugated goat anti-rabbit IgG (A11011, Thermo Fisher). Sections were counterstained with DAPI, mounted in Fluoromount-G (0100-01, Southern Biotech), and imaged using a Nikon 90i upright microscope equipped with Photometrics CoolSNAP-ES monochrome camera (Roper). Regions of the uterine–placental interface containing cytokeratin-positive cells were quantified using ImageJ software, as previously described (Dominguez et al, 2025; Rosario et al, 2008).

### Immunohistochemistry of trophoblast organoids

Organoids were recovered from Matrigel using Cell Recovery Solution (354253, Corning) and dissociated by gentle pipetting. After 30 min incubation on ice, samples were centrifuged (600×*g*, 5 min), washed with PBS, and fixed in 4% paraformaldehyde on ice for 30 min. Fixed organoids were suspended in 20% sucrose solution and incubated at 4 °C overnight. The sucrose solution was subsequently replaced with 1 mL of warm gelatin/sucrose solution and incubated at 37 °C for 15 min. Blocks of gelatin containing trophoblast organoids were frozen for 1–2 min and stored at −80 °C until processed for cryosectioning. Sections were permeabilized in PBS containing 4% BSA and 0.5% Triton X-100. Primary antibodies to HLA-G (1:300, phycoerythrin (PE)-labeled, ab24384, Abcam), CGB (1:500, ab9528, Abcam) and TP63 (1:500, ab124762, Abcam) were incubated with tissue sections overnight at 4 °C, followed by secondary antibody incubation. Sections were counterstained with DAPI, mounted in Fluoromount-G (0100-01, Southern Biotech), and imaged using a Nikon 90i upright microscope equipped with Photometrics CoolSNAP-ES monochrome camera (Roper).

### Western blotting

Human trophoblast cell lysates were processed using radioimmunoprecipitation assay lysis buffer (sc-24948A, Santa Cruz Biotechnology) and protein concentrations determined using the DC Protein Assay Kit (5000112, Bio-Rad). Proteins (50  µg/lane) were separated by sodium dodecyl sulfate polyacrylamide gel electrophoresis. Separated proteins were electrophoretically transferred to polyvinylidene difluoride membranes (10600023, GE Healthcare) for 1 h at 25 V on a semi-dry transfer apparatus (Bio-Rad). Membranes were subsequently blocked with 5% milk for 1 h at room temperature, followed by incubation with antibodies to PPARG (1:200, 2435S, Cell Signaling Technology or GAPDH (1:5000, AM4300, Invitrogen) in 5% milk overnight at 4 °C. After primary antibody incubation, the membranes were washed in Tris-buffered saline with Tween 20 (TBST) three times (10 min/wash) at room temperature. The membranes were then incubated with anti-mouse immunoglobulin G conjugated to horseradish peroxidase (HRP; 1:5000, 7076, Cell Signaling Technology) or anti-rabbit immunoglobulin G conjugated to horseradish peroxidase (HRP; 1:5000, 5127, Cell Signaling Technology) in 5% milk for 1 h at room temperature, washed in TBST three times (10 min/wash) at room temperature, immersed in Immobilon Crescendo Western HRP Substrate (WBLUR0500, Sigma-Aldrich), and luminescence detected using Chemi Doc MP Imager (Bio-Rad). The integrated densities of proteins separated by western blotting were quantified using NIH ImageJ. PPARG protein intensities were normalized to GAPDH protein intensities. All quantifications were performed on at least three independent biological replicates.

### RNA-seq analysis

Transcript profiles were generated from control and PPARG shRNA-1-treated CT27 human TS cells following EVT cell differentiation. Three independent lentiviral transductions for each shRNA were analyzed. RNA was isolated and integrity assessed using an Agilent 2100 Bioanalyzer. cDNA libraries were prepared with Illumina TruSeq RNA preparation kits according to the manufacturer’s instructions. Barcoded cDNA libraries were multiplexed onto a TruSeq paired-end flow cell and sequenced (100-bp paired-end reads) with a TruSeq 200-cycle sequencing-by-synthesis kit. Samples were sequenced on an Illumina NovaSeq platform at the KUMC Genome Sequencing Facility. Reads from *.fastq files were mapped to the human reference genome (GRCh37) using CLC Genomics Workbench 12.0 (Qiagen). Transcript abundance was expressed as total reads, and a false discovery rate of 0.05 was used as a cutoff for significant differential expression. Statistical significance was calculated by empirical analysis of digital gene expression followed by Bonferroni’s correction.

### ChIP-seq analysis

PPARG ChIP was performed on CT27 human TS cells following EVT cell differentiation using commercially available SimpleChIP^®^ Enzymatic Chromatin Immunoprecipitation Kit (Magnetic Beads, 9003, Cell Signaling Technology) and PPARG antibody (2435S, Cell Signaling Technology). Three independent replicates were processed. The ChIP-seq library was constructed using the Tecan Ovation Ultralow DNA System V2 (NuGEN Technologies, Redwood City, CA) and sequenced on an Illumina NovaSeq platform. The sequence quality of the raw FASTQ files was evaluated by fastqc (version 0.11.9). Quality control for the FASTQ files was performed by HTStream (version 1.3.3; https://github.com/s4hts/HTStream) to remove: (i) PhiX reads (by hts_SeqScreener with the default setting); (ii) duplicates (by hts_SuperDeduper, -e 250000); (iii) adapter sequences (by hts_AdapterTrimmer); (iv) unknown nucleotides (N) (by hts_NTrimmer); (v) low-quality reads (i.e., quality score <20 in a 10-bp sliding window by hts_QWindowTrim, -q 20 -w 10); (vi) short reads (<50 bp) and orphaned reads from a pair (by hits_LengthFilter). The trimmed reads were aligned to the human genome (GRCh38.p14) using BWA mem (version 0.7.17-r1188) (Li and Durbin, 2009). Quality control of the alignment includes removal of the secondary alignments, low-quality alignments (quality score <30), and alignments in the ENCODE blacklisted regions (https://zenodo.org/records/1491733) using samtools (version 1.19.2) (Danecek et al, 2021) and bedtools (version 2.31.1) (Quinlan and Hall, 2010).

For each biological replicate, peaks were independently identified using MACS3 (version 3.0.0b1) (Zhang et al, 2008) with fold enrichment >1.5 and *q* value <0.01. Bigwig files were created by deeptools (version 3.5.1) (Ramírez et al, 2016) to visualize peaks in the UCSC genome browser (https://genome.ucsc.edu/s/kchen7/hg38_p14_Pparg_ChIPseq). The final peak list was defined as the peaks reported in all three biological replicates through cross-referencing the peak regions using bedtools intersect (3). Genomic features of the peak regions and motif analysis were performed using HOMER (version 5.1) scripts annotatePeaks.pl and findMotifsGenome.pl (Heinz et al, 2010). Gene ontology (GO) enrichment analysis was executed using GREAT (version 4.0.4) (McLean et al, 2010). Peak-associated genes were identified using GREAT and cross-referenced with the differentially expressed genes identified by RNA-seq analysis (with fold change <1.5 and *q* value <0.05). The overlapping genes were analyzed by clusterProfiler (version 4.14.0) (Yu, 2024) to identify the enriched GO terms of the Biological Process Ontology (*p* value <0.05 and *q* value <0.05). Codes for all described analyses are available (https://github.com/Tuteja-Lab/ChIP_seq_pparg).

### Identification of potential PPARG gene targets in rat invasive trophoblast cells

Uniform manifold approximation and projection (UMAP) plots for *Pparg* and *Prl7b1* were generated from rat gd 19.5 uterine–placental interface single-cell RNA sequencing (Gene Expression Omnibus database accession number GSE206086). Gene expression profiles and open chromatin containing PPARG DNA binding motifs identified in rat invasive trophoblast cells [(Scott et al, 2022; Data ref: Scott et al, 2022; Vu et al, 2023; Data ref: Vu et al, 2023)] were integrated and ascribed a function through manual annotation using the UniProt (https://www.uniprot.org) and National Center for Biotechnology (https://www.ncbi.nlm.nih.gov/) databases.

### Statistics

Statistical analyses were performed with GraphPad Prism 10.2.3 software. Statistical comparisons were evaluated using Student’s *t*-test or one-way analysis of variance with Dunnett’s post hoc test. Statistical significance was determined as *P* < 0.05.

### Study approval

All protocols using rats were approved by the University of Kansas Medical Center Animal Care and Use Committee, Kansas City, Kansas (protocol number: 22-01-220).

## Supplementary information

Appendix

Peer Review File

Dataset EV1

Dataset EV2

Dataset EV3

Dataset EV4

Dataset EV5

Dataset EV6

Dataset EV7

Dataset EV8

Dataset EV9

Source data Fig. 1

Source data Fig. 2

Source data Fig. 3

Source data Fig. 4

Source data Fig. 6

Source data Fig. 7

Source data Fig. 8

Expanded View Figures

## Data Availability

RNA-seq and ChIP-seq datasets are available at the Gene Expression Omnibus database, https://www.ncbi.nlm.nih.gov/geo/ (GSE302291 and GSE302293). The source data of this paper are available in the following database record, https://www.ebi.ac.uk/biostudies/bioimages/studies/S-BIAD2948. The source data of this paper are collected in the following database record: biostudies:S-SCDT-10_1038-S44319-026-00896-0.

## References

[CR1] Ahmadian M, Suh JM, Hah N, Liddle C, Atkins AR, Downes M, Evans RM (2013) PPARγ signaling and metabolism: the good, the bad and the future. Nat Med. 10.1038/nm.3159 PMC3870016 23652116

[CR2] Ain R, Canham LN, Soares MJ (2003) Gestation stage-dependent intrauterine trophoblast cell invasion in the rat and mouse: novel endocrine phenotype and regulation. Dev Biol 260:176–190 10.1016/s0012-1606(03)00210-0 12885563

[CR3] Ain R, Konno T, Canham LN, Soares MJ (2006) Phenotypic analysis of the rat placenta. Methods Mol Med 121:295–313 10.1385/1-59259-983-4:293 16251750

[CR4] Asami-Miyagishi R, Iseki S, Usui M, Uchida K, Kubo H, Morita I (2004) Expression and function of PPARgamma in rat placental development. Biochem Biophys Res Commun 315:497–501 10.1016/j.bbrc.2004.01.074 14766236

[CR5] Aye ILMH, Tong S, Charnock-Jones DS, Smith GCS (2025) The human placenta and its role in reproductive outcomes revisited. Physiol Rev 105:2305–2376 10.1152/physrev.00039.2024 PMC7617900 40497429

[CR6] Barak Y, Nelson MC, Ong ES, Jones YZ, Ruiz-Lozano P, Chien KR, Koder A, Evans RM (1999) PPAR gamma is required for placental, cardiac, and adipose tissue development. Mol Cell 4:585–595 10.1016/s1097-2765(00)80209-9 10549290

[CR7] Berger J, Moller DE (2002) The mechanisms of action of PPARs. Annu Rev Med 53:409–435 10.1146/annurev.med.53.082901.104018 11818483

[CR8] Brosens I, Pijnenborg R, Vercruysse L, Romero R (2011) The “great obstetrical syndromes” are associated with disorders of deep placentation. Am J Obstet Gynecol 204:193–201 10.1016/j.ajog.2010.08.009 PMC3369813 21094932

[CR9] Brosens I, Puttemans P, Benagiano G (2019) Placental bed research: I. The placental bed: from spiral arteries remodeling to the great obstetrical syndromes. Am J Obstet Gynecol 221:437–456 10.1016/j.ajog.2019.05.044 31163132

[CR10] Calkins K, Devaskar SU (2011) Fetal origins of adult disease. Curr Probl Pediatr Adolesc Health Care 41:158–176 10.1016/j.cppeds.2011.01.001 PMC4608552 21684471

[CR11] Carter AM, Enders AC (2004) Comparative aspects of trophoblast development and placentation. Reprod Biol Endocrinol 2:46 10.1186/1477-7827-2-46 PMC455692 15236656

[CR12] Chakraborty D, Cui W, Rosario GX, Scott RL, Dhakal P, Renaud SJ, Tachibana M, Rumi MAK, Mason CW, Krieg AJ et al (2016) HIF-KDM3A-MMP12 regulatory circuit ensures trophoblast plasticity and placental adaptations to hypoxia. Proc Natl Acad Sci USA 113:E7212–E7221 10.1073/pnas.1612626113 PMC5135375 27807143

[CR13] Chakraborty D, Rumi MAK, Konno T, Soares MJ (2011) Natural killer cells direct hemochorial placentation by regulating hypoxia-inducible factor dependent trophoblast lineage decisions. Proc Natl Acad Sci USA 108:16295–16300 10.1073/pnas.1109478108 PMC3182743 21900602

[CR14] Chang W-L, Liu Y-W, Dang Y-L, Jiang X-X, Xu H, Huang X, Wang Y-L, Wang H, Zhu C, Xue L-Q et al (2018) PLAC8, a new marker for human interstitial extravillous trophoblast cells, promotes their invasion and migration. Development 145:dev148932 10.1242/dev.148932 PMC5825838 29361555

[CR15] Danecek P, Bonfield JK, Liddle J, Marshall J, Ohan V, Pollard MO, Whitwham A, Keane T, McCarthy SA, Davies RM et al (2021) Twelve years of SAMtools and BCFtools. GigaScience 10:giab008 10.1093/gigascience/giab008 PMC7931819 33590861

[CR16] Dhakal P, Soares MJ (2017) Single step PCR-based genetic sex determination for rat tissues and cells. Biotechniques 62:232–233 10.2144/000114548 PMC5831150 28528577

[CR17] Dominguez EM, Moreno-Irusta A, Scott RL, Iqbal K, Soares MJ (2025) TFAP2C is a key regulator of intrauterine trophoblast cell invasion and deep hemochorial placentation. JCI Insight 10:e186471 10.1172/jci.insight.186471 PMC11790029 39625795

[CR18] Erlebacher A (2013) Immunology of the maternal-fetal interface. Annu Rev Immunol 31:387–411 10.1146/annurev-immunol-032712-100003 23298207

[CR19] Feige JN, Gelman L, Michalik L, Desvergne B, Wahli W (2006) From molecular action to physiological outputs: peroxisome proliferator-activated receptors are nuclear receptors at the crossroads of key cellular functions. Prog Lipid Res 45:120–159 10.1016/j.plipres.2005.12.002 16476485

[CR20] Fisher SJ (2015) Why is placentation abnormal in preeclampsia?. Am J Obstet Gynecol 213:S115–S122 10.1016/j.ajog.2015.08.042 PMC4592742 26428489

[CR21] Fournier T, Guibourdenche J, Handschuh K, Tsatsaris V, Rauwel B, Davrinche C, Evain-Brion D (2011) PPARγ and human trophoblast differentiation. J Reprod Immunol 90:41–49 10.1016/j.jri.2011.05.003 21704384

[CR22] Fournier T, Tsatsaris V, Handschuh K, Evain-Brion D (2007) PPARs and the placenta. Placenta 28:65–76 10.1016/j.placenta.2006.04.009 16834993

[CR23] Georgiades P, Ferguson-Smith AC, Burton GJ (2002) Comparative developmental anatomy of the murine and human definitive placentae. Placenta 23:3–19 10.1053/plac.2001.0738 11869088

[CR24] Gluckman PD, Hanson MA, Cooper C, Thornburg KL (2008) Effect of in utero and early-life conditions on adult health and disease. N Engl J Med 359:61–73 10.1056/NEJMra0708473 PMC3923653 18596274

[CR25] Guo Q, Choi J, Lee M, Kim J (2025) PPARG-centric transcriptional re-wiring during differentiation of human trophoblast stem cells into extravillous trophoblasts. Nucleic Acids Res 53:gkaf669 10.1093/nar/gkaf669 PMC12288872 40705926

[CR26] Harris LK (2010) Review: trophoblast-vascular cell interactions in early pregnancy: how to remodel a vessel. Placenta 31:S93–S98 10.1016/j.placenta.2009.12.012 20060584

[CR27] Harris LK, Benagiano M, D’Elios MM, Brosens I, Benagiano G (2019) Placental bed research: II. Functional and immunological investigations of the placental bed. Am J Obstet Gynecol 221:457–469 10.1016/j.ajog.2019.07.010 31288009

[CR28] Heinz S, Benner C, Spann N, Bertolino E, Lin YC, Laslo P, Cheng JX, Murre C, Singh H, Glass CK (2010) Simple combinations of lineage-determining transcription factors prime cis-regulatory elements required for macrophage and B cell identities. Mol Cell 38:576–589 10.1016/j.molcel.2010.05.004 PMC2898526 20513432

[CR29] Hemberger M, Nozaki T, Masutani M, Cross JC (2003) Differential expression of angiogenic and vasodilatory factors by invasive trophoblast giant cells depending on depth of invasion. Dev Dyn 227:185–191 10.1002/dvdy.10291 12761846

[CR30] Holdsworth-Carson SJ, Lim R, Mitton A, Whitehead C, Rice GE, Permezel M, Lappas M (2010) Peroxisome proliferator-activated receptors are altered in pathologies of the human placenta: gestational diabetes mellitus, intrauterine growth restriction and preeclampsia. Placenta 31:222–229 10.1016/j.placenta.2009.12.009 20045185

[CR31] Iqbal K, Dominguez EM, Nixon B, Moreno-Irusta A, Crnkovich B, Scott RL, Vu HTH, Tuteja G, Vivian JL, Soares MJ (2024) Conditionally mutant animal model for investigating the invasive trophoblast cell lineage. Development 151:dev202239 10.1242/dev.202239 PMC10820817 38112206

[CR32] Jeyarajah MJ, Jaju Bhattad G, Kelly RD, Baines KJ, Jaremek A, Yang F-HP, Okae H, Arima T, Dumeaux V, Renaud SJ (2022) The multifaceted role of GCM1 during trophoblast differentiation in the human placenta. Proc Natl Acad Sci USA 119:e2203071119 10.1073/pnas.2203071119 PMC9894182 36442132

[CR33] Kadam L, Kohan-Ghadr HR, Drewlo S (2015) The balancing act – PPAR-γ’s roles at the maternal-fetal interface. Syst Biol Reprod Med 61:65–71 10.3109/19396368.2014.991881 PMC6364849 25475254

[CR34] Karvas RM, Khan SA, Verma S, Yin Y, Kulkarni D, Dong C, Park K, Chew B, Sane E, Fischer LA et al (2022) Stem-cell-derived trophoblast organoids model human placental development and susceptibility to emerging pathogens. Cell Stem Cell 29:810–825.e8 10.1016/j.stem.2022.04.004 PMC9136997 35523141

[CR35] Kaufmann P, Black S, Huppertz B (2003) Endovascular trophoblast invasion: implications for the pathogenesis of intrauterine growth retardation and preeclampsia. Biol Reprod 69:1–7 10.1095/biolreprod.102.014977 12620937

[CR36] Kim M, Jang YJ, Lee M, Guo Q, Son AJ, Kakkad NA, Roland AB, Lee B-K, Kim J (2024) The transcriptional regulatory network modulating human trophoblast stem cells to extravillous trophoblast differentiation. Nat Commun 15:1285 10.1038/s41467-024-45669-2 PMC10861538 38346993

[CR37] Kubota N, Terauchi Y, Miki H, Tamemoto H, Yamauchi T, Komeda K, Satoh S, Nakano R, Ishii C, Sugiyama T et al (1999) PPAR gamma mediates high-fat diet-induced adipocyte hypertrophy and insulin resistance. Mol Cell 4:597–609 10.1016/s1097-2765(00)80210-5 10549291

[CR38] Kuna M, Dhakal P, Iqbal K, Dominguez EM, Kent LN, Muto M, Moreno-Irusta A, Kozai K, Varberg KM, Okae H et al (2023) CITED2 is a conserved regulator of the uterine–placental interface. Proc Natl Acad Sci USA 120:e2213622120 10.1073/pnas.2213622120 PMC9934066 36626551

[CR39] Li H, Durbin R (2009) Fast and accurate short read alignment with Burrows–Wheeler transform. Bioinformatics 25:1754–1760 10.1093/bioinformatics/btp324 PMC2705234 19451168

[CR40] Liu Y, Fan X, Wang R, Lu X, Dang Y-L, Wang H, Lin H-Y, Zhu C, Ge H, Cross JC et al (2018) Single-cell RNA-seq reveals the diversity of trophoblast subtypes and patterns of differentiation in the human placenta. Cell Res 28:819–832 10.1038/s41422-018-0066-y PMC6082907 30042384

[CR41] Maltepe E, Fisher SJ (2015) Placenta: the forgotten organ. Annu Rev Cell Dev Biol 31:523–552 10.1146/annurev-cellbio-100814-125620 26443191

[CR42] McLean CY, Bristor D, Hiller M, Clarke SL, Schaar BT, Lowe CB, Wenger AM, Bejerano G (2010) GREAT improves functional interpretation of cis-regulatory regions. Nat Biotechnol 28:495–501 10.1038/nbt.1630 PMC4840234 20436461

[CR43] Meinhardt G, Waldhäusl H, Lackner AI, Wächter J, Maxian T, Höbler A-L, Vondra S, Kunihs V, Saleh L, Haslinger P et al (2025) The multifaceted roles of the transcriptional coactivator TAZ in extravillous trophoblast development of the human placenta. Proc Natl Acad Sci USA 122:e2426385122 10.1073/pnas.2426385122 PMC12037006 40228123

[CR44] Moreno-Irusta A, Basu MK, Dominguez EM, Chen TS, Smith SH, Varberg KM, Okae H, Arima T, Soares MJ (2026a) Developmental landmarks and cellular transitions during extravillous trophoblast cell differentiation. Proc Natl Acad Sci USA 123:e2529836123 10.1073/pnas.2529836123 PMC13168549 42090250

[CR45] Moreno-Irusta A, Urosevic J, Iqbal K, Seetharam AS, Nteeba J, Scott RL, Kuna M, Muto M, Kozai K, Celic A et al (2026b) Fundamental and unique roles of PLAC1 in the regulation of rat and human trophoblast cell development. Development 153:dev205290 10.1242/dev.205290 PMC13245914 42007670

[CR46] Muto M, Chakraborty D, Varberg KM, Moreno-Irusta A, Iqbal K, Scott RL, McNally RP, Choudhury RH, Aplin JD, Okae H et al (2021) Intersection of regulatory pathways controlling hemostasis and hemochorial placentation. Proc Natl Acad Sci USA 118:e2111267118 10.1073/pnas.2111267118 PMC8685669 34876522

[CR47] Okae H, Toh H, Sato T, Hiura H, Takahashi S, Shirane K, Kabayama Y, Suyama M, Sasaki H, Arima T (2018) Derivation of human trophoblast stem cells. Cell Stem Cell 22:50–63.e6 10.1016/j.stem.2017.11.004 29249463

[CR48] Parast MM, Yu H, Ciric A, Salata MW, Davis V, Milstone DS (2009) PPARγ regulates trophoblast proliferation and promotes labyrinthine trilineage differentiation. PLoS One 4:e8055 10.1371/journal.pone.0008055 PMC2778869 19956639

[CR49] Pavan L, Tarrade A, Hermouet A, Delouis C, Titeux M, Vidaud M, Thérond P, Evain-Brion D, Fournier T (2003) Human invasive trophoblasts transformed with simian virus 40 provide a new tool to study the role of PPARγ in cell invasion process. Carcinogenesis 24:1325–1336 10.1093/carcin/bgg074 12807721

[CR50] Pijnenborg R, Robertson WB, Brosens I, Dixon G (1981) Review article: trophoblast invasion and the establishment of haemochorial placentation in man and laboratory animals. Placenta 2:71–91 10.1016/s0143-4004(81)80042-2 7010344

[CR51] Pijnenborg R, Vercruysse L, Hanssens M (2006) The uterine spiral arteries in human pregnancy: facts and controversies. Placenta 27:939–958 10.1016/j.placenta.2005.12.006 16490251

[CR52] Quinlan AR, Hall IM (2010) BEDTools: a flexible suite of utilities for comparing genomic features. Bioinformatics 26:841–842 10.1093/bioinformatics/btq033 PMC2832824 20110278

[CR53] Rai A, Cross JC (2014) Development of the hemochorial maternal vascular spaces in the placenta through endothelial and vasculogenic mimicry. Dev Biol 387:131–141 10.1016/j.ydbio.2014.01.015 24485853

[CR54] Ramírez F, Ryan DP, Grüning B, Bhardwaj V, Kilpert F, Richter AS, Heyne S, Dündar F, Manke T (2016) deepTools2: a next generation web server for deep-sequencing data analysis. Nucleic Acids Res 44:W160–W165 10.1093/nar/gkw257 PMC4987876 27079975

[CR55] Rätsep MT, Felker AM, Kay VR, Tolusso L, Hofmann AP, Croy BA (2015) Uterine natural killer cells: supervisors of vasculature construction in early decidua basalis. Reproduction 149:R91–R102 10.1530/REP-14-0271 25342175

[CR56] Renaud SJ, Scott RL, Chakraborty D, Rumi MAK, Soares MJ (2017) Natural killer-cell deficiency alters placental development in rats. Biol Reprod 96:145–158 10.1095/biolreprod.116.142752 PMC6366547 28395334

[CR57] Roberts RM, Green JA, Schulz LC (2016) The evolution of the placenta. Reproduction 152:R179–R189 10.1530/REP-16-0325 PMC5033709 27486265

[CR58] Rodie VA, Young A, Jordan F, Sattar N, Greer IA, Freeman DJ (2005) Human placental peroxisome proliferator-activated receptor delta and gamma expression in healthy pregnancy and in preeclampsia and intrauterine growth restriction. J Soc Gynecol Investig 12:320–329 10.1016/j.jsgi.2005.03.004 15979543

[CR59] Rosario GX, Konno T, Soares MJ (2008) Maternal hypoxia activates endovascular trophoblast cell invasion. Dev Biol 314:362–375 10.1016/j.ydbio.2007.12.007 PMC2266816 18199431

[CR60] Sauer S (2015) Ligands for the nuclear peroxisome proliferator-activated receptor gamma. Trends Pharmacol Sci 36:688–704 10.1016/j.tips.2015.06.010 26435213

[CR61] Schaiff WT, Barak Y, Sadovsky Y (2006) The pleiotropic function of PPARγ in the placenta. Mol Cell Endocrinol 249:10–15 10.1016/j.mce.2006.02.009 16574314

[CR62] Schaiff WT, Bildirici I, Cheong M, Chern PL, Nelson DM, Sadovsky Y (2005) Peroxisome proliferator-activated receptor-gamma and retinoid X receptor signaling regulate fatty acid uptake by primary human placental trophoblasts. J Clin Endocrinol Metab 90:4267–4275 10.1210/jc.2004-2265 15827101

[CR63] Schaiff WT, Carlson MG, Smith SD, Levy R, Nelson DM, Sadovsky Y (2000) Peroxisome proliferator-activated receptor-gamma modulates differentiation of human trophoblast in a ligand-specific manner. J Clin Endocrinol Metab 85:3874–3881 10.1210/jcem.85.10.6885 11061552

[CR64] Scott RL, Vu HTH, Jain A, Iqbal K, Tuteja G, Soares MJ (2022) Conservation at the uterine–placental interface. Proc Natl Acad Sci USA 119:e2210633119 10.1073/pnas.2210633119 PMC9565169 36191208

[CR65] Scott RL, Vu HTH, Jain A, Iqbal K, Tuteja G. Soares MJ (2022) Gene expression omnibus GSE206086. https://www.ncbi.nlm.nih.gov/geo/query/acc.cgi?acc=GSE206086

[CR66] Shalom-Barak T, Nicholas JM, Wang Y, Zhang X, Ong ES, Young TH, Gendler SJ, Evans RM, Barak Y (2004) Peroxisome proliferator-activated receptor gamma controls Muc1 transcription in trophoblasts. Mol Cell Biol 24:10661–10669 10.1128/MCB.24.24.10661-10669.2004 PMC533980 15572671

[CR67] Shimizu T, Oike A, Kobayashi EH, Sekiya A, Kobayashi N, Shibata S, Hamada H, Saito M, Yaegashi N, Suyama M et al (2023) CRISPR screening in human trophoblast stem cells reveals both shared and distinct aspects of human and mouse placental development. Proc Natl Acad Sci USA 120:e2311372120 10.1073/pnas.2311372120 PMC10742386 38085778

[CR68] Shukla V, Moreno-Irusta A, Varberg KM, Kuna M, Iqbal K, Galligos AM, Aplin JD, Choudhury RH, Okae H, Arima T et al (2024) NOTUM-mediated WNT silencing drives extravillous trophoblast cell lineage development. Proc Natl Acad Sci USA 121:e2403003121 10.1073/pnas.2403003121 PMC11459147 39325428

[CR69] Shukla V, Soares MJ (2022) Modeling trophoblast cell-guided uterine spiral artery transformation in the rat. Int J Mol Sci 23:2947 10.3390/ijms23062947 PMC8950824 35328368

[CR70] Soares MJ, Chakraborty D, Karim Rumi MA, Konno T, Renaud SJ (2012) Rat placentation: an experimental model for investigating the hemochorial maternal-fetal interface. Placenta 33:233–243 10.1016/j.placenta.2011.11.026 PMC3288880 22284666

[CR71] Soares MJ, Varberg KM, Iqbal K (2018) Hemochorial placentation: development, function, and adaptations. Biol Reprod 99:196–211 10.1093/biolre/ioy049 PMC6044390 29481584

[CR72] Tarrade A (2001) PPAR /RXR heterodimers control human trophoblast invasion. J Clin Endocrinol Metab 86:5017–5024 10.1210/jcem.86.10.7924 11600579

[CR73] Tarrade A, Schoonjans K, Guibourdenche J, Bidart JM, Vidaud M, Auwerx J, Rochette-Egly C, Evain-Brion D (2001) PPARγ/RXRα heterodimers are involved in human cgβ synthesis and human trophoblast differentiation. Endocrinology 142:4504–4514 10.1210/endo.142.10.8448 11564716

[CR74] Varberg KM, Dominguez EM, Koseva B, Varberg JM, McNally RP, Moreno-Irusta A, Wesley ER, Iqbal K, Cheung WA, Schwendinger-Schreck C et al (2023) Extravillous trophoblast cell lineage development is associated with active remodeling of the chromatin landscape. Nat Commun 14:4826 10.1038/s41467-023-40424-5 PMC10415281 37563143

[CR75] Varberg KM, Iqbal K, Muto M, Simon ME, Scott RL, Kozai K, Choudhury RH, Aplin JD, Biswell R, Gibson M et al (2021) ASCL2 reciprocally controls key trophoblast lineage decisions during hemochorial placenta development. Proc Natl Acad Sci USA 118:e2016517118 10.1073/pnas.2016517118 PMC7958375 33649217

[CR76] Velicky P, Knöfler M, Pollheimer J (2015) Function and control of human invasive trophoblast subtypes: intrinsic vs. maternal control. Cell Adh Migr 10:154–162 10.1080/19336918.2015.1089376 PMC4853032 26418186

[CR77] Vento-Tormo R, Efremova M, Botting RA, Turco MY, Vento-Tormo M, Meyer KB, Park J-E, Stephenson E, Polański K, Goncalves A et al (2018) Single-cell reconstruction of the early maternal-fetal interface in humans. Nature 563:347–353 10.1038/s41586-018-0698-6 PMC7612850 30429548

[CR78] Vu HTH, Scott RL, Iqbal K, Soares MJ, Tuteja G (2023) Core conserved transcriptional regulatory networks define the invasive trophoblast cell lineage. Development 150:dev201826 10.1242/dev.201826 PMC10445752 37417811

[CR79] Vu HTH, Scott RL, Iqbal K, Soares MJ, Tuteja G (2023) Gene expression omnibus GSE227943. https://www.ncbi.nlm.nih.gov/geo/query/acc.cgi?acc=GSE227943

[CR80] Waite LL, Louie RE, Taylor RN (2005) Circulating Activators of peroxisome proliferator-activated receptors are reduced in preeclamptic pregnancy. J Clin Endocrinol Metab 90:620–626 10.1210/jc.2004-0849 15562025

[CR81] Waite LL, Person EC, Zhou Y, Lim KH, Scanlan TS, Taylor RN (2000) Placental peroxisome proliferator-activated receptor-gamma is up-regulated by pregnancy serum. J Clin Endocrinol Metab 85:3808–3814 10.1210/jcem.85.10.6847 11061543

[CR82] Wang L-J, Chen C-P, Lee Y-S, Ng P-S, Chang G-D, Pao Y-H, Lo H-F, Peng C-H, Cheong M-L, Chen H (2022) Functional antagonism between ΔNp63α and GCM1 regulates human trophoblast stemness and differentiation. Nat Commun 13:1626 10.1038/s41467-022-29312-6 PMC8956607 35338152

[CR83] Wiemers DO, Ain R, Ohboshi S, Soares MJ (2003) Migratory trophoblast cells express a newly identified member of the prolactin gene family. J Endocrinol 179:335–346 10.1677/joe.0.1790335 14656203

[CR84] Xie J, Xu Y, Wan L, Wang P, Wang M, Dong M (2018) Involvement of follistatin-like 3 in preeclampsia. Biochem Biophys Res Commun 506:692–697 10.1016/j.bbrc.2018.10.139 30454705

[CR85] Yu G (2024) Thirteen years of clusterProfiler. Innovation 5:100722 10.1016/j.xinn.2024.100722 PMC11551487 39529960

[CR86] Zhang Y, Liu T, Meyer CA, Eeckhoute J, Johnson DS, Bernstein BE, Nusbaum C, Myers RM, Brown M, Li W et al (2008) Model-based analysis of ChIP-seq (MACS). Gen Biol 9:R137 10.1186/gb-2008-9-9-r137 PMC2592715 18798982

