# Supplementary material for: PPARG directs trophoblast cell fate and establishment of the uterine-placental interface
Source: EMBO Rep. 2026 Aug 20;27(18):5341–64. doi: 10.1038/s44319-026-00896-0 (PMC13601578; doi:10.1038/s44319-026-00896-0)
Supplement: Supplementary file 1 [file 44319_2026_896_MOESM19_ESM.pdf]

Expanded View Figures

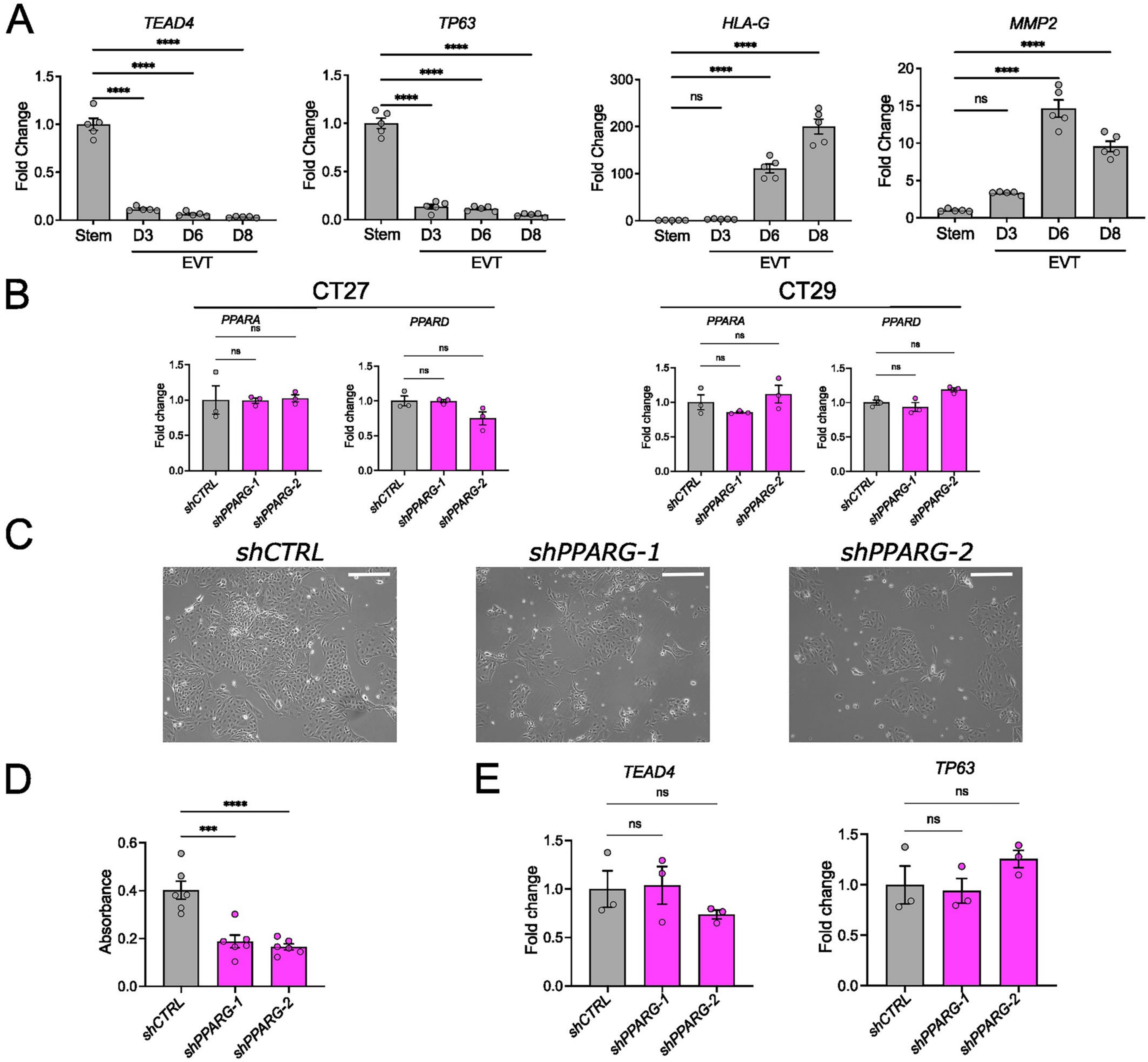

Figure EV1. Involvement of PPARG in the behavior of human trophoblast stem (TS) cells.

(**A**) Expression of TEAD4 and TP63 (stem-state-associated transcripts), and HLA-G and MMP2 (EVT cell-associated transcripts) in CT27 TS cells in the stem cell state (day 0, D0) and on day 3 (D3), day 6 (D6), and day 8 (D8) of EVT cell differentiation (D0 versus D3, D6, or D8: ****$p < 0.0001$, $n = 5$). (**B**) PPARA and PPARD transcript levels in CT27 and CT29 TS cells after 8 days of EVT cell differentiation in control shRNA (shCTRL), PPARG shRNA-1 (shPPARG-1), or PPARG shRNA-2 (shPPARG-2) treated cells (ns, nonsignificant; $n = 3$). (**C**) Phase-contrast images of TS cells in the stem state expressing shCTRL, shPPARG-1, or shPPARG-2 (scale bar = 100 µm). (**D**) Quantification of cell proliferation by crystal violet staining in shCTRL, shPPARG-1, or shPPARG-2 treated stem-state TS cells (shCTRL versus shPPARG-1: ***$p = 0.0001$; shCTRL versus shPPARG-2: ****$p < 0.0001$, $n = 6$). (**E**)*TEAD4* and *TP63* transcript levels in shCTRL, shPPARG-1, or shPPARG-2 treated TS cells in the stem state (ns nonsignificant; $n = 3$). RT-qPCR was used to measure transcript levels. Data were presented as mean ± standard error of the mean. Each data point represents a biological replicate. Statistical analyses were performed using ANOVA followed by Dunnett's post hoc test.

 © The Author(s)

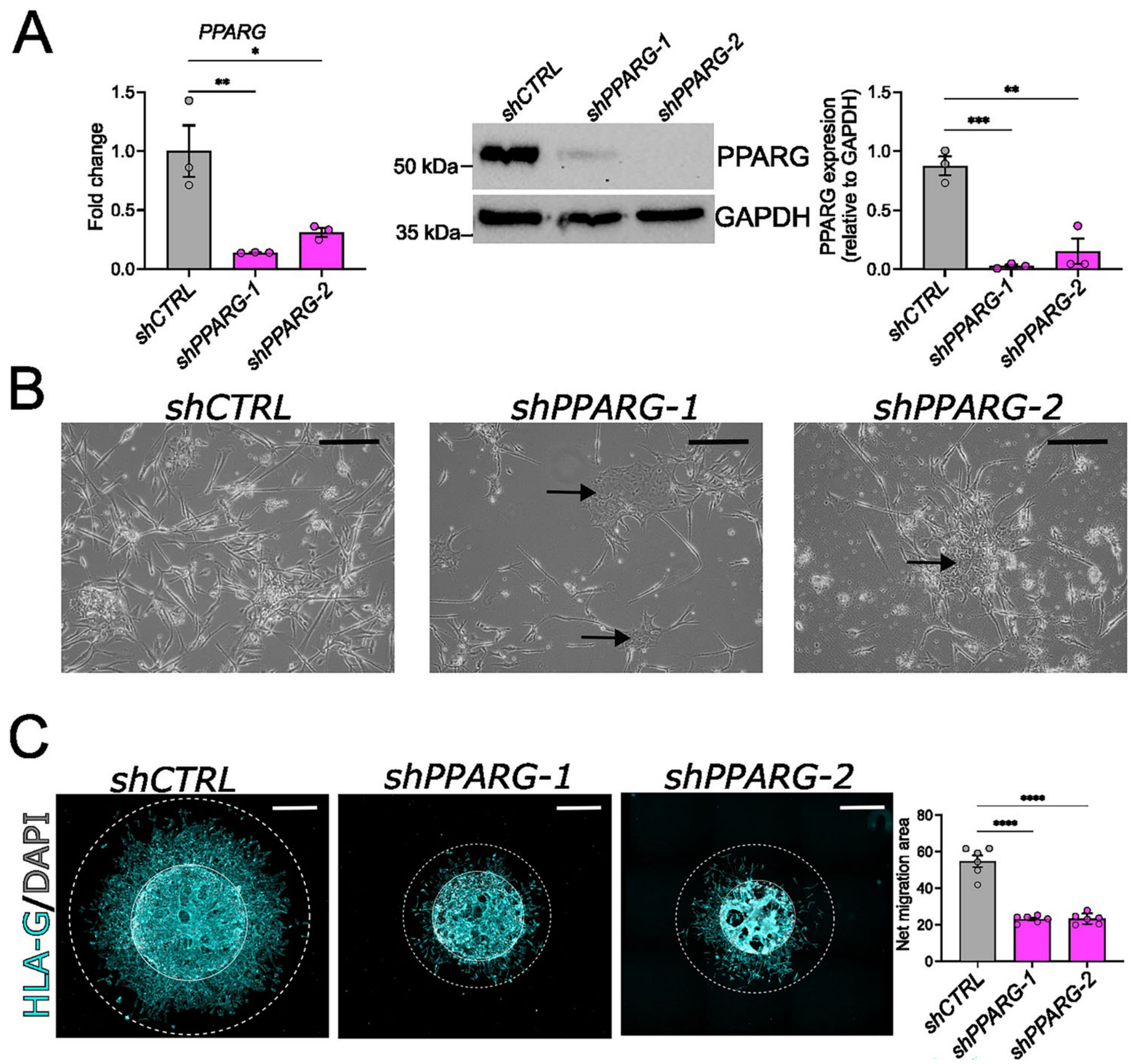

Figure EV2. Involvement of PPARG in the differentiation of CT29 (X,Y) trophoblast stem (TS) cells to extravillous trophoblast (EVT) cells.

(**A**) PPARG transcript and protein levels in CT29 TS cells after 8 days of EVT cells differentiation in control shRNA (shCTRL), PPARG shRNA-1 (shPPARG-1), or PPARG shRNA-2 (shPPARG-2) treatments (*PPARG* transcript for shCTRL versus shPPARG-1: $^{**}p = 0.0056$ and shCTRL versus shPPARG-2: $^{*}p = 0.0157$; PPARG protein for shCTRL versus shPPARG-1: $^{***}p = 0.0004$ and shCTRL versus shPPARG-2: $^{**}p = 0.0010$; $n = 3$). (**B**) Phase-contrast images of cells expressing shCTRL, shPPARG-1, or shPPARG-2 shRNAs (scale bar = 100 µm). Black arrows indicate cells exhibiting stem cell-like morphology. (**C**) HLA-G immunofluorescence detection of migrating cells (scale bar = 500 µm) and corresponding quantification of net migration area during exposure to EVT cell differentiation conditions in shCTRL, shPPARG-1, or shPPARG-2 treated cells (shCTRL versus shPPARG-1 or shPPARG-2: $^{****}p = <0.0001$; $n = 6$). RT-qPCR was used to measure transcript levels. Data were presented as mean ± standard error of the mean. Each data point represents a biological replicate. Statistical analysis was performed using ANOVA followed by Dunnett's post hoc test. White dashed lines demarcate areas of trophoblast cell migration.

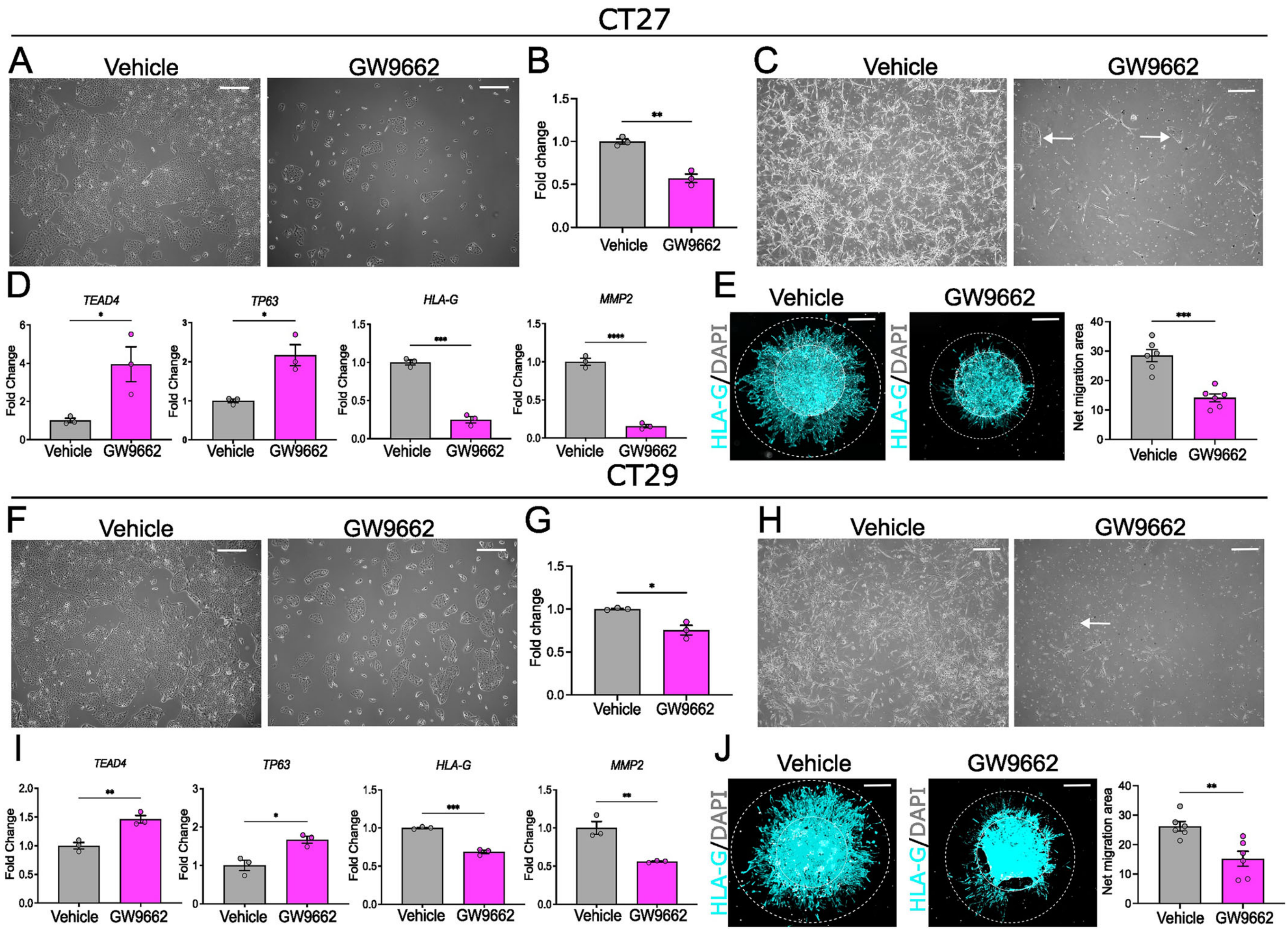

Figure EV3. Effects of a PPARG antagonist (GW9662) on EVT cell differentiation.

(**A**) Phase-contrast images of CT27 TS cells maintained in the stem cell state or treated with Vehicle (dimethyl sulfoxide) or GW9662 (10 µM), scale bar = 100 µm. (**B**) Quantification of cell numbers in Vehicle or GW9662-treated CT27 TS cells (Vehicle versus GW9662: $^{**}p = 0.0018$, $n = 3$). (**C**) Phase-contrast images of CT27 TS cells exposed to EVT cell differentiation conditions and treated with Vehicle or GW9662, scale bar = 100 µm. White arrows indicate cells exhibiting stem state-like morphology. (**D**) RT-qPCR measurements of *TEAD4* and *TP63* (stem-state-associated transcripts) and *HLA-G* and *MMP2* (EVT cell-associated transcripts) in Vehicle or GW9662-treated CT27 TS cells following 8 days of exposure to EVT cell differentiation culture conditions (Vehicle versus GW9662: *TEAD4*, $^{*}p = 0.0325$; *TP63*, $^{*}p = 0.0131$; *HLA-G*, $^{***}p = 0.0001$; *MMP2*, $^{****}p = <0.0001$; $n = 3$). (**E**) HLA-G immunofluorescence detection of migrating CT27 TS cells (scale bar = 500 µm) and corresponding quantification of net migration area during EVT cell differentiation in Vehicle or GW9662-treated cells (Vehicle versus GW9662: $^{***}p = 0.0002$, $n = 6$). White dashed lines demarcate the area of trophoblast cell migration. (**F**) Phase-contrast images of CT29 TS cells maintained in the stem cell state and treated with Vehicle or GW9662, scale bar = 100 µm. (**G**) Quantification of CT29 TS cell numbers treated with Vehicle or GW9662 (Vehicle versus GW9662: $^{*}p = 0.0121$, $n = 3$). (**H**) Phase-contrast images of CT29 TS cells exposed to EVT cell differentiation conditions and treated with Vehicle or GW9662, scale bar = 100 µm. White arrows indicate cells exhibiting stem state-like morphology. (**I**) RT-qPCR measurements of *TEAD4* and *TP63* (stem-state-associated transcripts) and HLA-G and MMP2 (EVT cell-associated transcripts) in Vehicle or GW9662-treated CT29 TS cells following 8 days of exposure to EVT cell differentiation culture conditions (Vehicle versus GW9662: *TEAD4*, $^{**}p = 0.0060$; *TP63*, $^{*}p = 0.0148$; *HLA-G*, $^{***}p = 0.0002$; *MMP2*, $^{**}p = 0.0067$, $n = 3$). (**J**) HLA-G immunofluorescence detection of migrating CT29 TS cells (scale bar = 500 µm) and corresponding quantification of net migration area during EVT cell differentiation in Vehicle or GW9662-treated cells (Vehicle versus GW9662: $^{**}p = 0.0043$, $n = 6$). White dashed lines demarcate the area of trophoblast cell migration. Data were presented as mean ± standard error of the mean. Each data point represents a biological replicate. Statistical analysis was performed using unpaired *t*-tests.

 © The Author(s)

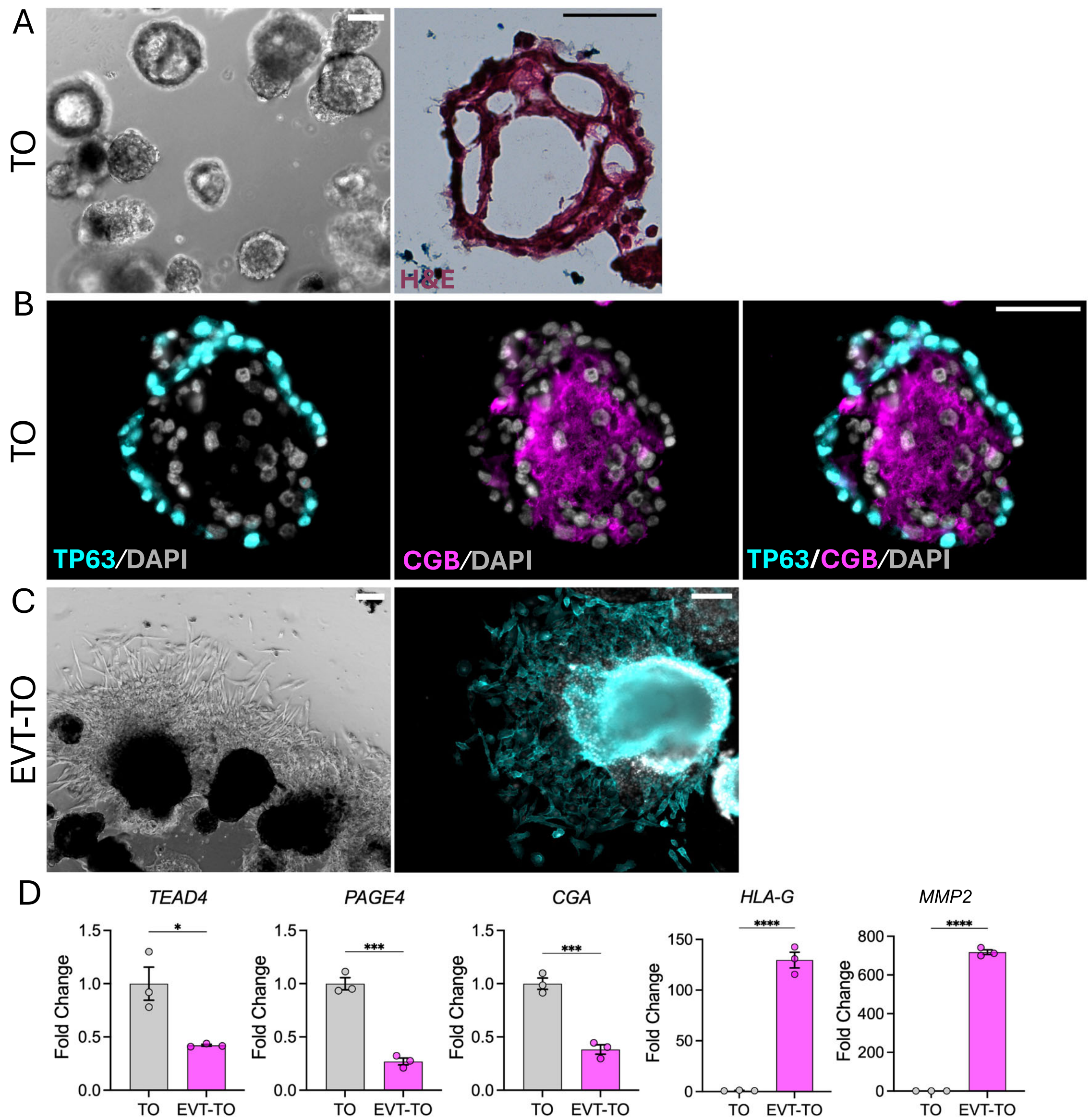

Figure EV4. Characterization of TS cell-derived trophoblast organoids (TO).

(**A**) Phase-contrast image and hematoxylin and eosin staining of TO, left panel: scale bar = 200 and right panel: scale bar = 50 µm. (**B**) TP63 (cyan) and CGB (magenta) immunodetection in TOs, scale bar = 50 µm. (**C**) HLA-G immunocytochemistry in TOs transferred to EVT cell differentiation conditions (EVT-TO), left panel: scale bar = 200 and right panel: scale bar = 50 µm. (**D**) RT-qPCR of stem state, syncytiotrophoblast and EVT cell-associated transcripts in TOs in the stem state and following exposure to EVT-TO (stem state versus EVT-TO: *TEAD4*, **p* = 0.0126; *PAGE4*, ****p* = 0.0001; *CGA*, ****p* = 0.0001; *HLA-G*, *****p* < 0.0001; *MMP2*, *****p* < 0.0001, *n* = 3). Data were presented as mean ± standard error of the mean. Each data point represents a biological replicate. Statistical analysis was performed using unpaired *t*-tests.

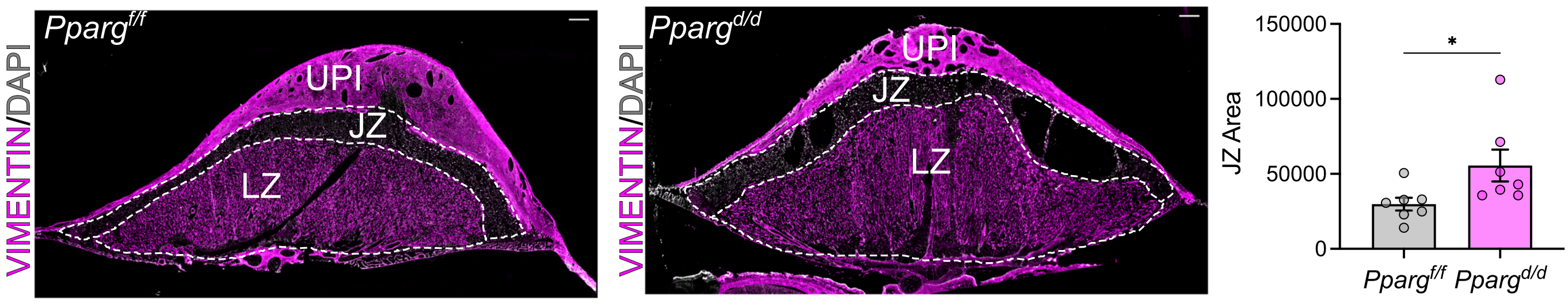

Figure EV5. Junctional zone areas in *Pparg*$^{f/f}$ and *Pparg*$^{d/d}$ placentation sites.

Left and central panels, Immunohistochemical staining for vimentin, which is positive in the labyrinth zone (LZ) and uterine–placental interface (UPI) and negative in the junctional zone (JZ). Right panel, quantification of JZ areas in *Pparg*$^{f/f}$ and *Pparg*$^{d/d}$ placentation sites. *Pparg*$^{d/d}$ JZs are significantly larger than *Pparg*$^{f/f}$ JZs (*$p = 0.0442$). Data were presented as mean ± standard error of the mean. Each data point represents a biological replicate from six independent pregnancies ($n = 7$). Statistical analyses were performed with an unpaired *t*-test. White dashed lines demarcate placental and uterine compartments.

© The Author(s)
