# Supplementary material for: PPARG directs trophoblast cell fate and establishment of the uterine-placental interface
Source: EMBO Rep. 2026 Aug 20;27(18):5341–64. doi: 10.1038/s44319-026-00896-0 (PMC13601578; doi:10.1038/s44319-026-00896-0)
Supplement: Supplementary file 2 [file 44319_2026_896_MOESM1_ESM.pdf]

Appendix

Table of content

Appendix Figures S1…..page 2

Appendix Figures S2…..page 3

Appendix Figures S3…..page 4

Appendix Figures S4…..page 5

Appendix tables S1.……page 6

Appendix tables S2.……page 7

Appendix tables S3.……page 8

Appendix tables S4.……page 9

Appendix tables S5.……page 10

Appendix tables S6.……page 11

Appendix Figures

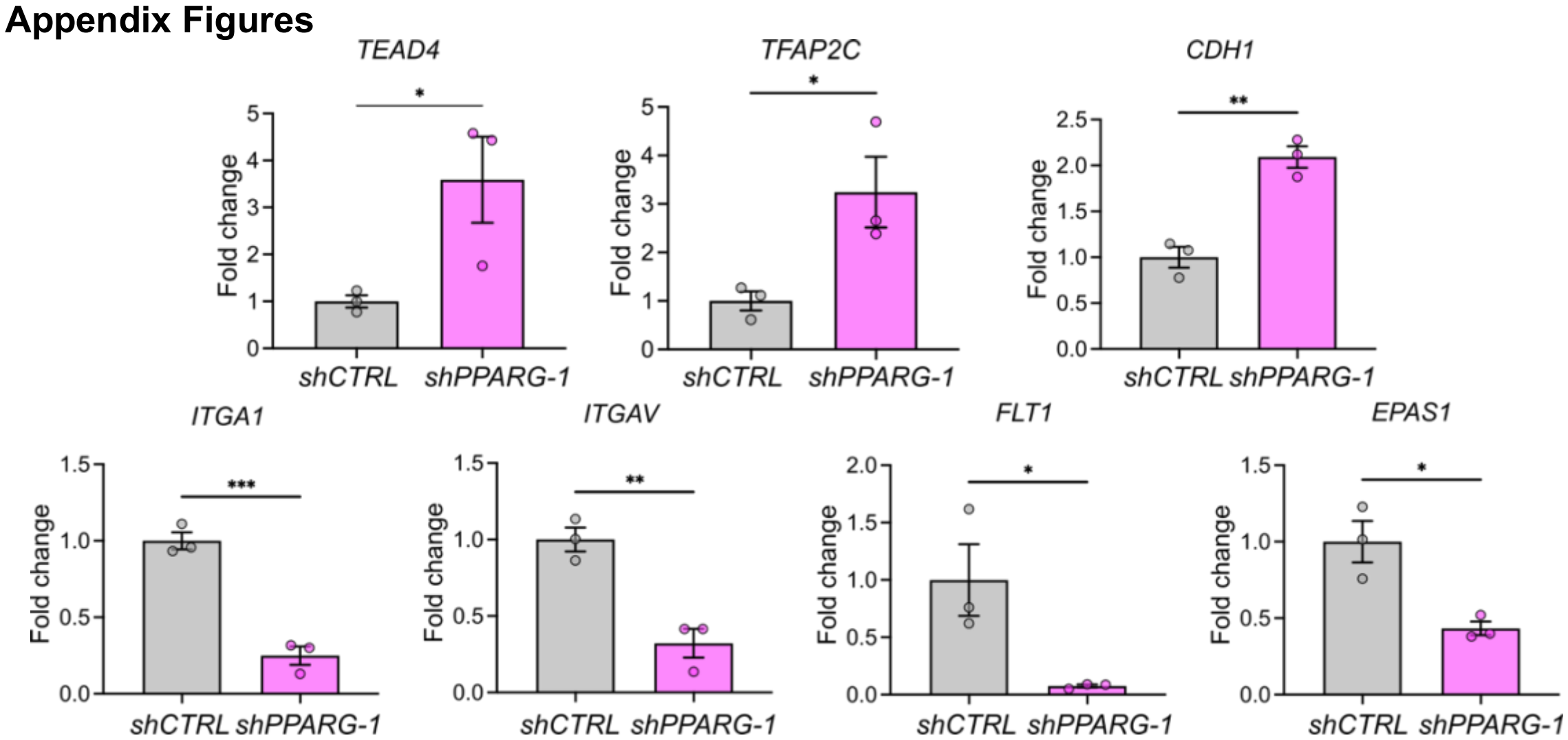

Appendix Figure S1. RNA-seq validation during EVT cell differentiation of CT27 (X,X) TS cells treated with control shRNA (shCTRL) and PPARG shRNA-1 (shPPARG-1). Transcripts were measured by RT-qPCR. Data are presented as mean ± standard error of the mean. Each data point represents a biological replicate. Statistical analyses were performed using unpaired *t*-tests: *$p<0.05$, **$p<0.01$, ***$p<0.001$.

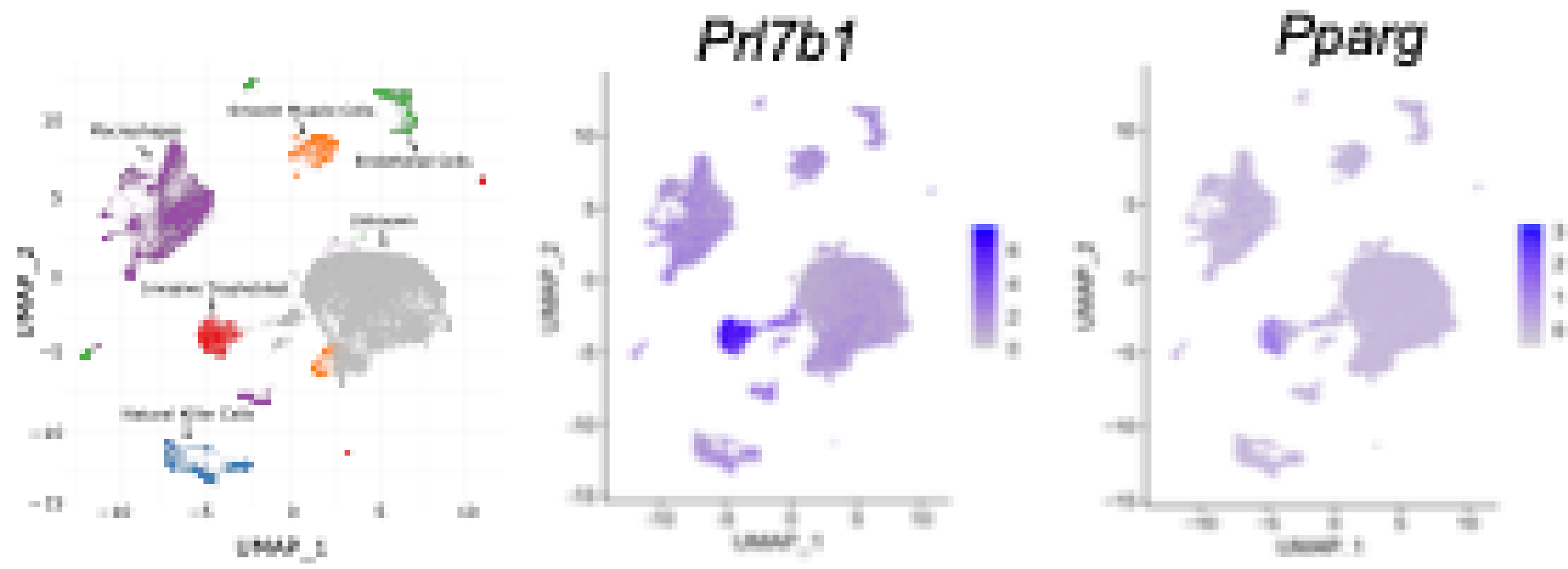

Appendix Figure S2. PPARG expression in invasive trophoblast cells within the rat uterine-placental interface. Uniform Manifold Approximation and Projection (UMAP) plots showing cell cluster identity (left), *Prl7b1* (center), and *Pparg* (right) transcript distribution from single-cell RNA sequencing of the rat uterine-placental interface at gestation day 19.5. (Scott *et al*, 2022).

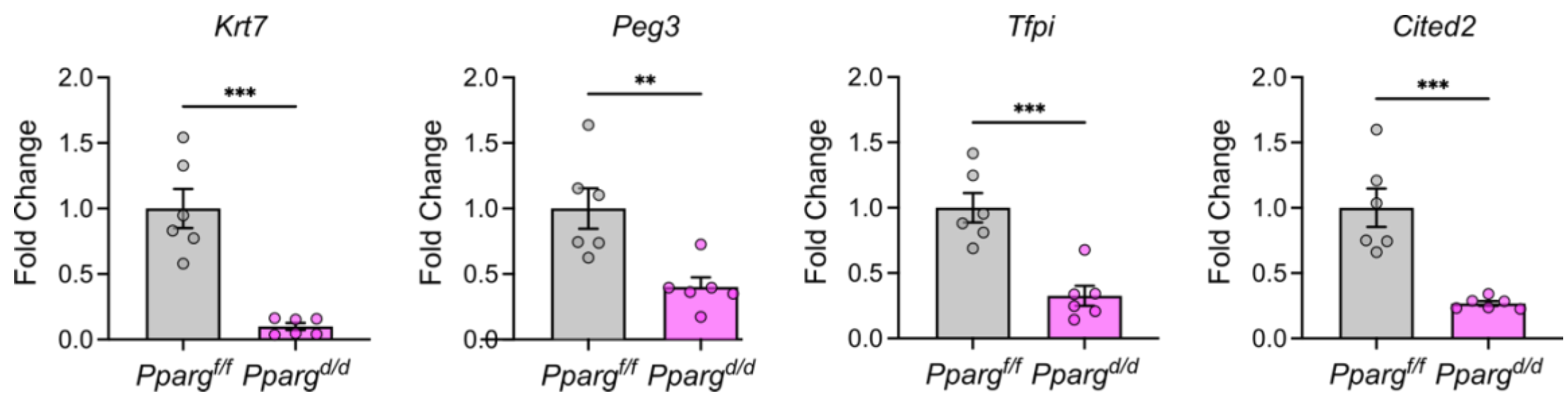

Appendix Figure S3. Assessment of invasive trophoblast cell-specific transcripts in *Pparg*$^{f/f}$ and *Pparg*$^{d/d}$ placentation sites. RT-qPCR analysis of *Krt7*, *Peg3*, *Tfpi*, and *Cited2* transcripts in the uterine-placental interface from gestation day 18.5 *Pparg*$^{f/f}$ and *Pparg*$^{d/d}$ placentation sites. Data are presented as mean ± standard error of the mean. Each data point represents a biological replicate obtained from six different pregnancies (n=6). Unpaired t-test: **p<0.01, ***p<0.001.

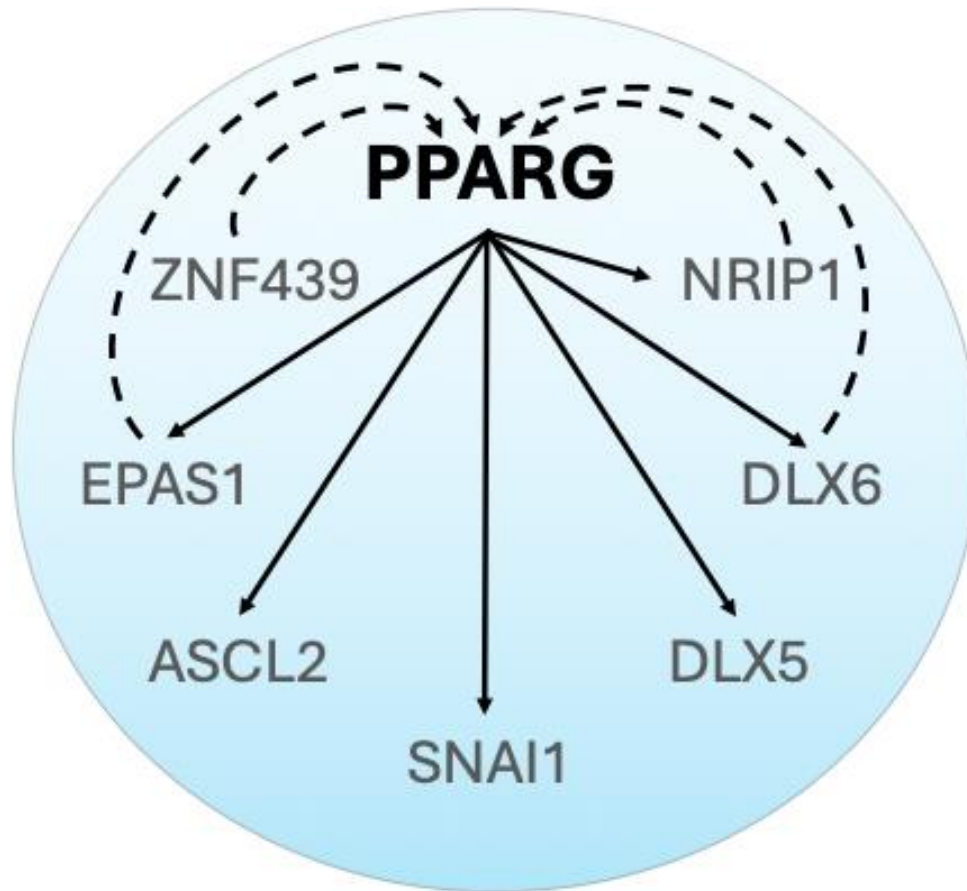

Appendix Figure S4. Transcription factor regulatory network directing EVT cell differentiation. Solid lines denote transcription factors regulated by PPARG and dashed lines refer to transcription factors regulating PPARG expression.

Appendix Tables

Appendix Table S1. Subset of genes expressed in rat invasive trophoblast cells possessing PPARG DNA binding motifs*

Extracellular matrix organization	Cell movement - migration	Cytoskeletal organization	Transcriptional regulation
Adamts3	*Arhgef16* *Ppp1r9a*	*Ankrd17*	*Ascl2* *Msx1*
Antxr2	*Dab2ip* *Raph1*	*Dsp*	*Bach2* *Ncor2*
Col4a1	*Dyrk2* *Setd3*	*Krt7*	*Chd7* *Peg3*
Fbln1	*Fgd6* *Setd3*	*Krt8*	*Cited2* *Phc1*
Lama4	*Frmd4b* *Stk10*	*Krt18*	*Ets2* *Pnrc1*
Ncam1	*Fscn1* *Unc5b*	*Mtcl1*	*Gata3* *Prdm15*
Nid1	*Furin*	*Setd3*	*Hdac4* *Rybp*
Tenm4		*Sptbn2*	*Irx3* *Tcf4*
			Med12l *Tfap2c*
			Zfhx3

*Analyses were based on previously published single cell RNA-seq and single nucleus ATAC-seq datasets from the rat uterine-placental interface (Scott *et al*, 2022; Vu *et al*, 2023).

Appendix Table S2. shRNA sequences for in vitro inhibition of PPARG in human TS cells

Name	Sequence
shPPARG-1	CAAGTAACTCTCCTCAAATAT
shPPARG-2	GACAGCGACTTGGCAATATTT
shCONTROL	CCTAAGGTTAAGTCGCCCTCG

Appendix Table S3. Guide RNA and templates used for inserting loxp sites flanking Exons 2 and 3 of the rat *Pparg* gene.

gRNA / DNA Template	Sequence
Pparg (5' of Exon 2)<br>*gRNA*	ATATATTTGAAATTTGCTAAGTTTTAGAGCTATGCT
Pparg (3' of Exon 3)<br>*gRNA*	CATCTCAATGCATCTTGCAGGTTTTAGAGCTATGCT
Pparg 5' loxP template<br>*(5' of Exon 2)*	AGAGTTTCCATTAGGCTCTGGAGACCTGATGGACAGCAAGAT<br>GATTAGAGTTAGGAACACTATATCATATATTTGAAATTTGCATA<br>ACTTCGTATAGCATACATTATACGAAGTTATTAAGGGAGCAAAT<br>CATAAGTCCTCTCATCTCAGAAACTCAAATAAAGTCTGACTTC<br>CTTCTTACCTGGATGGTGCTAGAAAGG
Pparg 3' loxP template<br>*(3' of Exon 3)*	AAGAAAAGCTAATAAAAGCAGGGTTACTCTGCCCCTCTTCCC<br>CTTCTCCAAAATGATTCTTTGAGGCATCTCAATGCATCTTGAT<br>AACTTCGTATAGCATACATTATACGAAGTTATCAGAGGTTTTTT<br>TGAGAGGATTTACCCAAGGTATCACAGCAAGATAGTGAATGG<br>TAAGCTAGTACTAGGACCTAAATTTTCTG

Appendix Table S4. Primers used for genotyping and sex determination.

Primer Name	Forward Primer	Reverse Primer
5' Floxed Pparg	AGGGAAATGAACTAGACGCAGA	AGAGAGGGAGCTGAATGAGT
3'Floxed Pparg	TTTTGCCTCTCCCATTTCCT	ACCCAAGTGATCCATGCCTT
Prl7b1Cre	TGCTGGAAGATGGCGATTAG	GGTTCCCAGCAAAGTACCAC
Kdm5d	TTGGTGAGATGGCTGATTCC	GGTTTCTTAAACCGTCGCC
Kdm5c	TTTGTACGACTAGGCCCCAC	GGTTTCTTAAACCGTCGCC

Appendix Table S5. RT-qPCR human primer list.

Primer Name	Forward Primer	Reverse Primer
PPARG	ACTTTGGGATCAGCTCCGTG	GGAGATGCAGGCTCCACTTT
PPARA	CTATCATTTGCTGTGGAGATCG	AAGATATCGTCCGGGTGGTT
PPARD	GTCACACAACGCTATCCGTTT	AGGCATTGTAGATGTGCTTGG
TEAD4	CAGGTGGTGGAGAAAGTTGAGA	GTGCTTGAGCTTGTGGATGAAG
TP63	AGAAACGAAGATCCCCAGATGA	CTGTTGCTGTTGCCTGTACGTT
HLA-G	CCACCACCCTGTCTTTGACTAT	ACGTCCTGGGTCTGGTCCT
MMP2	TGGCACCCATTTACACCTACAC	ATGTCAGGAGAGGCCCCATAGA
MMP15	CCTCCCTGAGCTAGAACCCT	CTCAGACCCCTGAGGCTAGT
PLAC8	CGTCGCAATGAGGACTCTCT	CTCTTGATTTGGCAAAGAGTACAA
TFPI	AACACCTGTGAGGATCCAGTG	TCCCAATGGCTGGGTTGTAG
DLX6	CCGAACTGGCAGCTTCCTTA	GGGTCGCTCTCATGAGGATT
FLT1	GTGACGTGTGGTCCTTTGG	CTGGCAGAACTCCTCATTGAT
FSTL3	CTACATCTCCTCGTGCCACA	TCTTCTGCAGACTCACCACCT
TFAP2C	AAGCCGCTCATGTGACTCTC	TCGGCTTCACAGACATAGGC
CDH1	CCCGGGACAACGTTTATTAC	GCTGGCTCAAGTCAAAGTCC
ITGA1	AATTGGCTCTAGTCACCATTGTT	CAAATGAAGCTGCTGACTGGT
ITGAV	GAGGGAAGCAAAGGACCGT	GAAGAAATCCACGGCGAAGC
PAGE4	CGTAAAGTAGAAGGTGATTG	ATGCTTAGGATTAGGTGGAG
CGA	TGCTGTGTAGCTAAATCATATAAC	TCAAGACAGCACTTGGTAAAACA
EPAS1	GTCAGCCCACAAGGTGTCA	CACAGTCATATCTGGTCAGTTCG
POLR2A	TCCGTATTCGCATCATGAAC	TCATCCATCTTGTCCACCAC

Appendix Table S6. RT-qPCR rat primer list.

Primer Name	Forward Primer	Reverse Primer
Pparg	GACTGAGTGTGACGACAAGATTT	CGATGGCCATGAGGGAGTTT
Peg3	AAGTTCACGTCCACTCCGTC	CGTCTGGTCTTGTTCGTGGA
Cited2	TCTTGGCTGCATGAACTTTG	GGGAGACAGCCAACTTGAAA
Tfpi	GCCCGAGGAAGACGATGATA	TCCGCCTTCATTGCACAG
Prl5a1	TCCACACCAGACATTCCAGA	TTTCCAGGAAGCCAACATTC
Prl7b1	CCGTCATACTGTCTCAGCACATC	AGCTGTTGAGACCATTGACAACAA
Gapdh	GACATGCCGCCTGGAGAAAC	AGCCCAGGATGCCCTTTAGT
Krt7	CGGAATGGGACCTGTGAA	GTAGATGTGGTCTTGATGGAATAGG
Krt8	TGGGCCAGGAGAAGCTGAA	CACATCCTTCTTGATGAGGACAAA
Prf1	GGCACTCAAGAACCTTCC	CTCAAGCAGTCTCCTACC
Klrb1c	GTCTCCAGGGCATAAGCAAG	AAGAAGGATCAGCGTAGCACA
Klrb1a	CGTTCACACAGGTTGGCTTT	TGGCTCCACTGATGGTTTTT
Klrb1	GGTCTCGCTGACTGTTCTTTG	TTGTTGTCCTTTTCCCTTTG
Ncr1	ATGGGAACATCCAAGCAGAG	ACAGGCTCACTGGGAAAAGA
Acta2	CCTCTTCCAGCCATCTTTCAT	CGAGAGGACGTTGTTAGCATAG
