# Supplementary material for: PPARG directs trophoblast cell fate and establishment of the uterine-placental interface
Source: EMBO Rep. 2026 Aug 20;27(18):5341–64. doi: 10.1038/s44319-026-00896-0 (PMC13601578; doi:10.1038/s44319-026-00896-0)
Supplement: Supplementary file 3 [file 44319_2026_896_MOESM2_ESM.pdf]

EMBO reports

PPARG directs trophoblast cell fate and establishment of the uterine-placental interface

Esteban Dominguez, Ayelen Moreno-Irusta, Khursheed Iqbal, Keting Chen, Alex Finlinson, Marc Parrish, Hiroaki Okae, Takahiro Arima, Geetu Tuteja, and Michael Soares

Corresponding author(s): Esteban Dominguez (edominguez@kumc.edu) , Michael Soares (msoares@kumc.edu)

Review Timeline:

Submission Date:	13th Nov 25
Editorial Decision:	31st Dec 25
Revision Received:	6th Mar 26
Editorial Decision:	3rd Jul 26
Revision Received:	13th Jul 26
Accepted:	21st Jul 26

Editor: Martina Rembold

Transaction Report:

(Note: With the exception of the correction of typographical or spelling errors that could be a source of ambiguity, letters and reports are not edited. Depending on transfer agreements, referee reports obtained elsewhere may or may not be included in this compilation. Referee reports are anonymous unless the Referee chooses to sign their reports.)

Dear Dr. Dominguez

Thank you for the submission of your manuscript to EMBO reports. I apologize for the delay in handling it, but we have meanwhile received the full set of referee reports as well as referee cross-comments that are all pasted below.

As you will see, the referees acknowledge that the findings are potentially interesting but they also point out quite a number of technical and experimental concerns. Referee #2 points out an earlier study reporting on a role of PPARG in the EVT lineage, but given the added value of the rat animal model, we have decided to invite you to revise your manuscript, with the understanding that all referee requests are addressed with one exception, i.e., the identification of genes which are directly regulated by Pparg in the invasive rat trophoblast (referee #2). This analysis is not required for potential publication at EMBO Reports.

I acknowledge that this will require a substantial revision and significant amount of work and am happy to discuss the revision outline and the experiments that can be done in a realistic timeframe of 3 - 5 months in more detail in a Zoom call. Please let me know if you want to go through the revisions.

Acceptance of the manuscript will depend on a positive outcome of a second round of review. It is EMBO reports policy to allow a single round of major revision only and acceptance or rejection of the manuscript will therefore depend on the completeness of your responses included in the next, final version of the manuscript.

We realize that it is difficult to revise to a specific deadline. In the interest of protecting the conceptual advance provided by the work, we recommend a revision within 3 months (31st March 2026). Please discuss the revision progress ahead of this time with the editor if you require more time to complete the revisions.

You can either publish the study as a short report or as a full article. For short reports, the revised manuscript should not exceed 27,000 characters (including spaces but excluding materials & methods and references) and 5 main plus 5 expanded view figures. The results and discussion sections must further be combined, which will help to shorten the manuscript text by eliminating some redundancy that is inevitable when discussing the same experiments twice. For a normal article there are no length limitations, but it should have more than 5 main figures and the results and discussion sections must be separate. In both cases, the entire materials and methods must be included in the main manuscript file.

IMPORTANT NOTE: we perform an initial quality control of all revised manuscripts before re-review. Your manuscript will FAIL this control and the handling will be DELAYED if the following APPLIES:
1) A data availability section providing access to data deposited in public databases is missing. If you have not deposited any data, please add a sentence to the data availability section that explains that.
2) Your manuscript contains statistics and error bars based on n=2. Please use scatter blots in these cases. No statistics should be calculated if n=2.

When submitting your revised manuscript, please review the submission guidelines (https://link.springer.com/journal/44319/submission-guidelines#cms-Revised-submissions) and carefully review the instructions that follow below. Failure to include requested items will delay the evaluation of your revision.

1) a .docx formatted version of the manuscript text (including legends for main figures, EV figures and tables). Please make sure that the changes are highlighted to be clearly visible.

2) individual production quality figure files as .eps, .tif, .jpg (one file per figure). See hhttps://media.springernature.com/original/springer-cms/rest/v1/content/27825798/data/v1 for more info on how to prepare your figures.

3) We replaced Supplementary Information with Expanded View (EV) Figures and Tables that are collapsible/expandable online. A maximum of 5 EV Figures can be typeset. EV Figures should be cited as 'Figure EV1, Figure EV2" etc... in the text and their respective legends should be included in the main text after the legends of regular figures.

- For the figures that you do NOT wish to display as Expanded View figures, they should be bundled together with their legends in a single PDF file called *Appendix*, which should start with a short Table of Content. Appendix figures should be referred to in the main text as: "Appendix Figure S1, Appendix Figure S2" etc. See 'Expanded View' section of the submission guidelines.

- Additional Tables/Datasets should be labeled and referred to as Table EV1, Dataset EV1, etc. Legends have to be provided in a separate tab in case of .xls files. Alternatively, the legend can be supplied as a separate text file (README) and zipped together with the Table/Dataset file.

4) a .docx formatted letter INCLUDING the reviewers' reports and your detailed point-by-point responses to their comments. As

part of the EMBO Press transparent editorial process, the point-by-point response is part of the Review Process File (RPF), which will be published alongside your paper.

5) a complete author checklist, which you can download from our author guidelines. Please insert information in the checklist that is also reflected in the manuscript. The completed author checklist will also be part of the RPF.

6) Please note that all corresponding authors are required to supply an ORCID ID for their name upon submission of a revised manuscript (<https://orcid.org/>), which can be linked to your profile in the manuscript submission system.

7) Before submitting your revision, primary datasets produced in this study need to be deposited in an appropriate public database. Please remember to provide a reviewer password if the datasets are not yet public. The accession numbers and database should be listed in a formal "Data Availability" section placed after Methods. Please note that the Data Availability Section is restricted to new primary data that are part of this study. * Note - All links should resolve to a page where the data can be accessed. *

If your study has not produced novel datasets, please mention this fact in the Data Availability Section.

8) At EMBO Press we ask authors to provide source data for the main manuscript figures. You will receive a separate email with instructions for providing source data with your revised manuscript, including how to upload and organize the files.

9) Our journal also encourages inclusion of *data citations in the reference list* to directly cite datasets that were re-used and obtained from public databases. Data citations in the article text are distinct from normal bibliographical citations and should directly link to the database records from which the data can be accessed. In the main text, data citations are formatted as follows: "Data ref: Smith et al, 2001" or "Data ref: NCBI Sequence Read Archive PRJNA342805, 2017". In the Reference list, data citations must be labeled with "[DATASET]". A data reference must provide the database name, accession number/identifiers and a resolvable link to the landing page from which the data can be accessed at the end of the reference.

10) Regarding data quantification, the following points must be specified in each figure legend:

- the name of the statistical test used to generate error bars and P values,

- the number (n) of independent experiments (please specify technical or biological replicates) underlying each data point,

- the nature of the bars and error bars (s.d., s.e.m.),

- If the data are obtained from n {less than or equal to} 5, show the individual data points in addition to the SD or SEM.
- If the data are obtained from n {less than or equal to} 2, use scatter blots showing the individual data points.

Discussion of statistical methodology can be reported in the materials and methods section, but figure legends should contain a basic description of n, P and the test applied.

- Please also include scale bars in all microscopy images.

11) The journal requires a statement specifying whether or not authors have competing interests (defined as all potential or actual interests that could be perceived to influence the presentation or interpretation of an article). In case of competing interests, this must be specified in your disclosure statement.

12) All Materials and Methods need to be described in the main text using our 'Structured Methods' format, which is required for all research articles. According to this format, the Methods section includes a Reagents and Tools Table (listing key reagents, experimental models, software and relevant equipment and including their sources and relevant identifiers) followed by a Methods and Protocols section describing the methods using a step-by-step protocol format. The aim is to facilitate adoption of the methodologies across labs. More information on how to adhere to this format as well as a downloadable template (.docx) for the Reagents and Tools Table can be found in our author guidelines.

We would also welcome the submission of cover suggestions, or motifs to be used by our Graphics Illustrator in designing a cover.

As part of the EMBO publication's Transparent Editorial Process, EMBO reports publishes online a Review Process File (RPF) to accompany accepted manuscripts. This File will be published in conjunction with your paper and will include the referee reports, your point-by-point response and all pertinent correspondence relating to the manuscript.

You are able to opt out of this by letting the editorial office know (emboreports@embo.org). If you do opt out, the Review Process File link will point to the following statement: "No Review Process File is available with this article, as the authors have

chosen not to make the review process public in this case."

I look forward to seeing a revised form of your manuscript when it is ready.

Yours sincerely,

Martina Rembold, PhD
Senior Editor
EMBO reports

==================

Referee #1:

The manuscript "PPARG directs trophoblast cell fate and establishment of the uterine-placental interface" by Dominguez, et al. investigates the function of PPARγ in invasive trophoblast and by implication, at least indirectly, in the remodeling of uterine spiral arterioles that often is altered in diseases of human pregnancy. This topic is of significant interest to EMBO readers. The experiments are mostly well designed and executed and the results support a role or roles for PPARγ in invasive trophoblast. However, some methods design and results deserve greater attention, and some aspects of the presentation could be made clearer.

The shRNA effects reported are most concerning because some controls for specificity are lacking. The results show effects in cells treated with shPPARG but not in cells treated with shCTRL However, these effects are not shown to be specific for PPARγ. Perhaps shRNA targeting any transcript, or other sequence-related transcripts, have the same effect. How were the shPPARG and shCONTROL sequences determined (and does shCONTROL target any known transcript?), and how were they validated as appropriate for sequence specificity. This is not a trivial concern, especially because PPARβ/δ and to some extent PPARα are also expressed by trophoblast. Potential solutions include: testing phenotype rescue by re-expressing PPARγ from a construct refractory to the shPPARG used here; confirming the effects observed using shPPARG targeting different regions of PPARγ; reporting the effects of shPPARG on expression of PPARβ/δ and PPARα.

Although subtle, it would also be preferable to use the term "shRNA" (singular) rather than "snRNAs" (pleural). Use is inconsistent and the latter unmistakenly implies that more than one shRNA sequence was used (as suggested be done in this review).

The Prl7b1-Cre rat animal model is potentially an effective experimental tool for testing the hypotheses presented. However, the expression specificity and recombination efficiency other than in invasive trophoblast have not been reported previously and are not mentioned here. For that reason, it is not possible to assign the effects reported solely to PPARγ in invasive trophoblast. Effects at other sites cannot be excluded, including the possibility of effects secondary to partial embryo PPARγ deficiency if Prl7b1-Cre shows unexpected embryonic expression. Tpbpα-Cre mice show an expression pattern in invasive trophoblast that at least superficially mimics that of Prl7b1-Cre in rats but also shows some expression in embryos. Experiments in Sox2-Cre mice suggest that embryo PPARγ is not involved in the placental phenotype (embryonic lethality) of PPARγ-deficiency. However, the same has not been shown in rats. Rats also more faithfully model some aspects of human placentation. For these reasons, and on general principles, the specificity of the Prl7b1-Cre rat should be addressed and the implications for interpretation of the experiments presented here discussed.

Designation of placental zones is not clear throughout the manuscript. In some cases (all PPARγd/d), an additional unannotated region exists between JZ and UPI (Fig. 8A right and others) and in other cases (all PPARγF/F) not (Fig. 8A left and others). This region may not be solely due to artifactual separation because it contains tissue (Fig. 8A right, 8B right, 8G right and others). Zone designation should be clarified and the potential consequences for interpretation of the results considered. Also, in some panels the JZ seems unexpectedly wide (compare Fig. 8 vs. Fig. C). Specifically designating the LZ/JZ boarder in each figure panel might clarify things.

Images of tissue overall are well done but some are too small to support the interpretations claimed: for example, Fig. 6 showing "co-localization". It is not possible to evaluate whether PPARγ is expressed in the same cells or in other cells in the same region as Krt8 and Prl7b1. In such cases, adding representative higher power insets in a "blank" region of the image would be much clearer without requiring redesign of the entire figure. Also, Cyan/Magenta is an effective colorblind-aware palette, but Yellow instead of Grey as a third color would better reveal nuclei, especially in cells not expressing PPARγ or other nuclear signals. The H&E in Fig. 7C shows very little that is consistent with hematoxylin staining.

The images of Western blots are overexposed precluding visual interpretation. For example, as presented Fig S2 A does not

support or refute the conclusion that PPARγ protein abundance is altered by shPPARG. This is especially problematic because quantification by chemiluminescence is described in methods but not presented in the manuscript. Appropriately exposed images and quantification should be presented.

Referee #2:

In the manuscript "PPARG directs trophoblast cell fate and establishment of the uterine-placental interface", Dominguez et al. report PPARG as a regulator of the invasive trophoblast differentiation in human and rat. The authors first show that PPARG is expressed in the cell column and extravillous trophoblast (EVT) cells of the human placenta. Using 2D human trophoblast stem cell (hTSC) cultures and 3D trophoblast organoids, they demonstrate that shRNA-mediated depletion of PPARG prevents EVT differentiation and is accompanied by broad changes in gene expression, including the downregulation of established EVT markers. To identify direct PPARG targets, the authors integrate PPARG ChIP-seq with RNA-seq upon PPARG depletion, identifying a shared subset of misregulated genes, including established EVT regulators. To assess the role of Pparg in vivo, the authors generated Paprg conditional KO rat placentas using Prl7B-Cre, achieving Pparg deletion specifically in the invasive lineage. The resulting Pparg KO placentas were enlarged and functionally less efficient, and their junctional zones contained prominent cyst-like structures. Additionally, the invasive lineage exhibited impaired invasion, accompanied by reduced expression of invasive markers and abnormal retention of natural killer cells.

Major comments:

- Part of the manuscript related to human trophoblast lacks novelty, as the essential function of PPARG in EVT lineage has recently been reported by Guo et al. in Nucleic Acids Res. (PMID: 40705926). Importantly, the study by Guo et al. was more comprehensive as it covered additional mechanistic and functional aspects. While independent validation is valuable, the present manuscript largely confirms already published observations.
- The 1.5-fold change threshold in the RNA-seq analysis is relatively lax. Stronger criteria or additional statistical justification would strengthen the analysis.
- The bioinformatic analysis of the RNA-seq and ChIP-seq data is superficial. For instance, the authors don't sufficiently discuss the substantial number of PPARG-regulated genes unrelated to EVT differentiation.
- Lack of protein-level validation for EVT markers. Aside from HLA-G, the authors don't demonstrate altered expression of other EVT markers on the protein level.
- TSC-derived trophoblast organoids are a well-established model and don't, in my opinion, require a whole figure for characterization.
- The authors don't address whether PPARG depletion affects STB differentiation.
- Only a single PPARG shRNA was tested, and no rescue experiments were performed.
- It would be beneficial to add WT and PPARG-KD RNA-seq tracks to Fig. 5G.
- The characterization of the Pparg conditional KO phenotype is superficial. The authors should perform global gene expression analysis in the Pparg-KO cells.
- It remains unknown which genes are directly regulated by Pparg in the invasive rat trophoblast. The authors should perform ChIP-seq on sorted placental cells. Alternatively, they could establish rat Pparg-KO TSC differentiation as a model system.
- The Discussion is focused on the trophoblast and placenta. Placing the findings in the broader context, e.g., cancer, and discussing aspects of the underlying molecular mechanism may be beneficial.

Minor comments:

- Several sentences would profit from rephrasing (e.g., line 102-104, line 106-108, line 154-158, line 161-162).
- line 105: "state" instead of "sate".
- The quality of figures needs improvement. For instance, graphs in figures 4A, 5A, and 5B lack proper labelling; portions of figures 4A, 4B, 4D, 5E, and 5F are difficult to read due to small fonts or misaligned labels.
- Fig.S4D: two graphs are labelled "PAGE4"
- Figure 7B: the JZ images in Ppargf/f and Ppargd/d appear to depict the same region with only minimal shifting; this raises concerns about potential image duplication and should be clarified.

In summary, the manuscript confirms the essential role of PPARG in human EVT differentiation and provides new insights into PPARG function in invasive rat trophoblast. While the rat data are of interest, the human findings lack novelty, and several key analyses, particularly those concerning data depth, mechanistic interpretation, and validation, are insufficient in their current form.

We would like to express our sincere gratitude to reviewers for their careful evaluations and constructive feedback. We have carefully considered their suggestions and have comprehensively addressed key concerns raised, as directed by the editor.

Below are responses to specific reviewers concerns and comments:

Referee #1:

1. *The shRNA effects reported are most concerning because some controls for specificity are lacking. The results show effects in cells treated with shPPARG but not in cells treated with shCTRL However, these effects are not shown to be specific for PPARγ. Perhaps shRNA targeting any transcript, or other sequence-related transcripts, have the same effect. How were the shPPARG and shCONTROL sequences determined (and does shCONTROL target any known transcript?), and how were they validated as appropriate for sequence specificity. This is not a trivial concern, especially because PPARβ/δ and to some extent PPARα are also expressed by trophoblast. Potential solutions include: testing phenotype rescue by re-expressing PPARγ from a construct refractory to the shPPARG used here; confirming the effects observed using shPPARG targeting different regions of PPARγ; reporting the effects of shPPARG on expression of PPARβ/δ and PPARα.*

Demonstrating sequence specificity is critical, particularly given the expression of other PPAR isoforms in trophoblast cells. We have clarified the design and validation of the shRNAs in the Methods section. Specifically, the shCONTROL construct corresponds to a non-targeting sequence that does not align with any known mammalian gene. Second, we have incorporated new experimental results using a second shRNA for PPARG (shPPARG2). These additional experiments demonstrate that both shRNAs (shPPARG1 and shPPARG2) selectively decrease PPARG expression at transcript and protein levels without affecting the transcript levels of PPARA or PPARD. These results are now included in the revised manuscript (see text below, Fig. 2A, and Fig. EV2D). Together, the clarified methods and the newly included experimental data strengthen the evidence that the effects observed are specific to PPARG disruption rather than nonspecific shRNA-mediated effects.

See lines 110-115:

… PPARG transcript and protein were significantly decreased in EVT cells stably expressing either of two different PPARG shRNAs when compared to expression of a control shRNA (Fig. 2A). The specificity of PPARG-targeting shRNAs was assessed by monitoring the expression of other members of the PPAR family. Expression of *PPARA* and *PPARD* transcripts were not affected following treatment with PPARG shRNAs (Fig. EV1B)…

See lines 381-383:

…An shRNA control (**shCTRL**, Plasmid 1864, Addgene), which does not recognize any known mammalian gene, was similarly subcloned into the pLKO.1 vector and used as a control…

2. *Although subtle, it would also be preferable to use the term "shRNA" (singular) rather than "snRNAs" (pleural). Use is inconsistent and the latter unmistakenly implies that more than one shRNA sequence was used (as suggested be done in this review).*

As indicated above, we have incorporated a second shRNA to PPARG in the analysis. We have corrected any misidentification of our use of a single shRNA versus multiple shRNAs.

3. *The Prl7b1-Cre rat animal model is potentially an effective experimental tool for testing the hypotheses presented. However, the expression specificity and recombination efficiency other than in invasive trophoblast have not been reported previously and are not mentioned here. For that reason, it is not possible to assign the effects reported solely to PPARγ in invasive trophoblast. Effects at other sites cannot be excluded, including the possibility of effects secondary to partial embryo PPARγ deficiency if Prl7b1-Cre shows unexpected embryonic expression. Tpbpα-Cre mice show an expression pattern in invasive trophoblast that at least superficially mimics that of Prl7b1-Cre in rats but also shows some expression in embryos. Experiments in Sox2-Cre mice suggest that embryo PPARγ is not involved in the placental phenotype (embryonic lethality) of PPARγ-deficiency. However, the same has not been shown in rats. Rats also more faithfully model some aspects of human placentation. For these reasons, and on general principles, the specificity of the Prl7b1-Cre rat should be addressed and the implications for interpretation of the experiments presented here discussed.*

Importantly, the expression, specificity, and recombination efficiency of the Prl7b1-Cre rat have been previously presented (Iqbal et al., Development, 2024, PMID: 38112206). In that study, Cre activity was validated using ZSGreen reporter and evidence presented indicating that Prl7b1-Cre was uniquely active in the invasive trophoblast cell lineage within the uterine-placental interface. These data provide direct experimental evidence supporting the cell lineage specificity of the Prl7b1-Cre model in rats.

In addition, the same Prl7b1-Cre rat model was successfully used to drive lineage-specific genetic manipulation in invasive trophoblast cells (Dominguez et al. JCI Insight, 2024, PMID: 39625795). In that study, Tfap2c conditional targeting using the Prl7b1-Cre rat line resulted in phenotypes confined to the invasive trophoblast cell compartment, further supporting the functional specificity and recombination efficiency of the Prl7b1-Cre in the invasive trophoblast cell lineage.

We agree with the reviewer that unexpected embryonic expression could complicate interpretation. However, based on the previously reported characterization of the Prl7b1-Cre, there is no evidence of embryonic Cre activity in this rat model (Iqbal et al., Development, 2024, PMID: 38112206).

We have revised the manuscript to explicitly reference the prior validation of the Prl7b1-Cre rat model, summarized its demonstrated lineage specificity and recombination efficiency, and discussed the value of the Prl7b1-Cre rat model for use in dissecting regulatory mechanisms controlling the invasive trophoblast cell lineage.

See lines 215-220:
…For these experiments we used a rat model possessing Cre recombinase inserted into the *Prl7b1* locus. As noted above, *Prl7b1* is robustly and specifically expressed within invasive trophoblast cells of the rat placentation site (Wiemers et al. 2003; Scott et al. 2022; Iqbal et al. 2024). The *Prl7b1-Cre* specifically acts within the invasive trophoblast cell lineage without anomalous activities in non-invasive trophoblast cells or other extraembryonic and embryonic tissues (Dominguez et al. 2024; Iqbal et al. 2024)…

4. *Designation of placental zones is not clear throughout the manuscript. In some cases (all PPARγd/d), an additional unannotated region exists between JZ and UPI (Fig. 8A right and others) and in other cases (all PPARγF/F) not (Fig. 8A left and others). This region may not be*

solely due to artifactual separation because it contains tissue (Fig. 8A right, 8B right, 8G right and others). Zone designation should be clarified and the potential consequences for interpretation of the results considered. Also, in some panels the JZ seems unexpectedly wide (compare Fig. 8 vs. Fig. C). Specifically designating the LZ/JZ boarder in each figure panel might clarify things.

Some of the confusion may have arisen from our failure to identify the location of the decidual compartment. We have re-examined all tissue sections and identified decidua as "D". We have also commented on changes to the decidua accompanying disruption of the *Pparg* gene.

Lines 230-231:
…An expanded decidual compartment was evident within *Pparg*$^{d/d}$ placentation sites (Fig. 7C)…

To further improve clarity, we have delineated the junctional zone versus labyrinth zone boundary in each figure panel containing rat placentation sites.

Regarding potential effects on the size of the junctional zone, we have included quantitative measurements of junctional zone areas (**Fig. EV5**). These measurements confirm that the junctional zone is expanded in the PPARG$^{d/d}$ placentas, consistent with their histological appearance.

5. *Images of tissue overall are well done but some are too small to support the interpretations claimed: for example, Fig. 6 showing "co-localization". It is not possible to evaluate whether PPARγ is expressed in the same cells or in other cells in the same region as Krt8 and Prl7b1. In such cases, adding representative higher power insets in a "blank" region of the image would be much clearer without requiring redesign of the entire figure. Also, Cyan/Magenta is an effective colorblind-aware palette, but Yellow instead of Grey as a third color would better reveal nuclei, especially in cells not expressing PPARγ or other nuclear signals. The H&E in Fig. 7C shows very little that is consistent with hematoxylin staining.*

We have added a new panel containing higher-magnification images of the uterine-placental interface (**Fig. 6C**). The insets allow clear visualization of invasive trophoblast cells positive for *Prl7b1* transcript (cyan) and PPARG transcript (magenta) that co-localize (white). DAPI staining was not included in this new image to facilitate observation of co-localization.

We have repeated and optimized the H&E staining and replaced the panel with an improved image showing clear nuclear hematoxylin signal and appropriate cytoplasmic eosin contrast.

6. *The images of Western blots are overexposed precluding visual interpretation. For example, as presented Fig S2 A does not support or refute the conclusion that PPARγ protein abundance is altered by shPPARG. This is especially problematic because quantification by chemiluminescence is described in methods but not presented in the manuscript. Appropriately exposed images and quantification should be presented.*

We have repeated the western blot experiments. The revised manuscript now includes appropriately exposed images that allow clear visual assessment of PPARG protein abundance. We also assessed PPARG band intensities normalized to GAPDH (Fig. 2A and Fig. EV2A) and present the relevant statistical analysis. We have updated the Methods section to clarify the quantification procedure and ensured that all corresponding densitometric analyses are now included in the manuscript.

See lines 530-532:

… The integrated densities of proteins separated by western blotting were quantified using NIH ImageJ. PPARG protein intensities were normalized to GAPDH protein intensities. All quantifications were performed on at least three independent biological replicates.

Referee #2:

Major comments:

1. *Part of the manuscript related to human trophoblast lacks novelty, as the essential function of PPARG in EVT lineage has recently been reported by Guo et al. in Nucleic Acids Res. (PMID: 40705926). Importantly, the study by Guo et al. was more comprehensive as it covered additional mechanistic and functional aspects. While independent validation is valuable, the present manuscript largely confirms already published observations.*

We acknowledge the overlap of some of the experiments using human trophoblast stem cells. However, the manuscript does more than provide an independent validation of the Guo et al. report. We also demonstrate that the actions of PPARG are not restricted to the X,X CT27 human trophoblast cell line and also demonstrate PPARG involvement in X,Y CT29 cells. Most importantly, and distinct from the Guo report, we provide an in vivo test of the conserved involvement of PPARG in regulation of the invasive trophoblast cell lineage.

2. *The 1.5-fold change threshold in the RNA-seq analysis is relatively lax. Stronger criteria or additional statistical justification would strengthen the analysis.*

As indicated in our report, our threshold was more than simply a 1.5-fold change. We also used appropriate statistical corrections, including P values of <0.05. The statistical analysis for the RNA-seq datasets is provided in detail in the Methods section of the report.

3. *The bioinformatic analysis of the RNA-seq and ChIP-seq data is superficial. For instance, the authors don't sufficiently discuss the substantial number of PPARG-regulated genes unrelated to EVT differentiation*.

We have provided pathway analysis of the PPARG regulated genes and have gone into greater detail regarding the impact of PPARG targets on the biology of trophoblast cells.

See lines 169-172:

…In addition, GO analysis identified PPARG target genes connected to response to hypoxia, lipid transport, and WNT signaling, which are also likely important in the transition from the stem state to mature differentiated EVT cells (Fig. 5E)…

4. *Lack of protein-level validation for EVT markers. Aside from HLA-G, the authors don't demonstrate altered expression of other EVT markers on the protein level.*

We provide morphological, gene expression, HLA-G protein expression, and cell migration as indices of EVT cell behavior. Collectively, this information provides a rigorous assessment of EVT cell differentiation.

5. *TSC-derived trophoblast organoids are a well-established model and don't, in my opinion, require a whole figure for characterization.*

Our intention in including this extended figure was not to redefine the model, but to document the specific features and differentiation capacity of the organoids generated and used in this study, thereby ensuring experimental transparency and reproducibility. This is especially important since it represents the first time our laboratory has incorporated trophoblast organoids into our research.

6. *The authors don't address whether PPARG depletion affects STB differentiation.*

A potential role for PPARG in the regulation of STB differentiation is an interesting question but is well beyond the scope of this study, which focused on the regulation of invasive trophoblast cell lineages using human trophoblast stem cells and the rat.

7. *Only a single PPARG shRNA was tested, and no rescue experiments were performed.*

We have added a second PPARG shRNA to our analysis. The efficacy of both shRNAs on CT27 and CT29 trophoblast stem cell lines were similar. Data generated with the two shRNAs are included in the revised manuscript (**Fig. 2A–C, Fig. EV 2A–C**).

See lines 110-112:
…PPARG transcript and protein were significantly decreased in EVT cells stably expressing either of two different PPARG shRNAs when compared to expression of a control shRNA (Fig. 2A)…

The reviewer is correct we did not perform rescue experiments. Please note that the essence of our experimental findings showing a role for PPARG in the differentiation of human trophoblast stem cells to EVT cells has been experimentally replicated (PMID: 40705926).

8. *It would be beneficial to add WT and PPARG-KD RNA-seq tracks to Fig. 5G.*

As requested, we have added both shCTRL and shPPARG1 RNA-seq tracks to Fig. 5G**.**

9. *The characterization of the Pparg conditional KO phenotype is superficial. The authors should perform global gene expression analysis in the Pparg-KO cells.*

We do not agree with this suggestion. We have performed a thorough analysis of the PPARG conditional mutant phenotype and conclusively demonstrated that PPARG regulates the invasive trophoblast cell lineage of the rat. Invasive trophoblast cells do not exist within the uterine-placental interface of PPARG conditional mutants. Examining the role of PPARG in the origin of invasive trophoblast cells within the junctional zone is highly meritorious but well beyond the aim of this report.

10. *It remains unknown which genes are directly regulated by Pparg in the invasive rat trophoblast. The authors should perform ChIP-seq on sorted placental cells. Alternatively, they could establish rat Pparg-KO TSC differentiation as a model system.*

We agree that identifying direct PPARG target genes in invasive trophoblast cells is valuable. In the past, we have isolated invasive trophoblast cells by enzymatic dissociation and flow cytometry. This approach was problematic in that the gene expression profile of invasive trophoblast cells changed dramatically during the procedure. We have had better success with single cell RNA-sequencing and single nucleus ATAC-sequencing, which were previously reported (Scott et al. 2022, PMID: 36191208; Vu et al. 2023, PMID: 37417811). We have included a subset of these analyses connecting PPARG to gene targets in invasive trophoblast cells of the rat (**Appendix Table 1**).

Rat trophoblast stem cells are a great model for examining the regulation of some aspects of trophoblast cell differentiation (Asanoma et al. Dev Biol, 2011, PMID: 21215265); however, culture conditions have not been optimized for directing differentiation to the invasive trophoblast cell lineage. Consequently, at this juncture, such an in vitro experiment, as described, would have limited utility.

11. *The Discussion is focused on the trophoblast and placenta. Placing the findings in the broader context, e.g., cancer, and discussing aspects of the underlying molecular mechanism may be beneficial.*

We do not yet possess the mechanistic insights regarding the actions of PPARG on invasive trophoblast cell development to effectively engage in a broader discussion on the molecular mechanism of PPARG action in other physiological or pathological states. This is an important research direction for the future.

<u>Minor comments</u>:

1. *Several sentences would profit from rephrasing (e.g., line 102-104, line 106-108, line 154-158, line 161-162).*

All indicated sentences (lines 99–100, 102–105, 155–156, and 160–161) have been restructured in the revised manuscript to improve clarity.

2. *line 105: "state" instead of "sate".*

The misspelled word has been corrected.

3. *The quality of figures needs improvement. For instance, graphs in figures 4A, 5A, and 5B lack proper labelling; portions of figures 4A, 4B, 4D, 5E, and 5F are difficult to read due to small fonts or misaligned labels.*

In the revised manuscript, all figures have been carefully updated to improve clarity and readability.

4. *Fig.S4D: two graphs are labelled "PAGE4"*

Labeling for Fig. EV4D has been corrected so that each graph now accurately reflects the correct gene.

5. *Figure 7B: the JZ images in Ppargf/f and Ppargd/d appear to depict the same region with only minimal shifting; this raises concerns about potential image duplication and should be clarified.*

We have acquired new images for the corresponding junctional zones (JZ) for both *Pparg*$^{f/f}$ and *Pparg*$^{d/d}$ specimens. These new images have been included in the revised figure, ensuring that each panel accurately represents independent regions and resolves any concerns about image duplication.

Dear Esteban,

Thank you for the submission of your manuscript to EMBO reports. As you know, we have received a mixed set of referee reports. While referee #1 supported publication in EMBO reports, referee #2 raised concerns regarding the advance provided and noted a number of missing analyses. You have provided a point-by-point response to these concerns, which I have further discussed with referee #1, who considered your arguments valid and some experiments outside the scope of the current study. I should further note that the identification of PPARG target genes in rats was not required for publication in EMBO reports. An additional consideration is that we were well aware of the study published by Guo et al, when we sent your manuscript to peer review but we also valued the in vivo analysis which in our opinion extended the earlier study. I copy the referee reports and the comments from referee #1 below my signature.

Given the feedback and support by referee #1 and the considerations noted above, I have decided to proceed with publication of your manuscript, under the condition that the remaining concerns raised are acknowledged in the manuscript and adequately discussed.

From the editorial side there are also a number of things that we need before we can proceed with the official acceptance of your study.

- Please place the Disclosure and competing interests statement' after the Acknowledgments.

- Please correct the following name discrepancy: Ayelen Moreno-Irusta in the manuscript vs. Ayelen Moreno in the submission system.

- "Methods and Protocols" should be "Methods".

- Nomenclature of EV figures: it should be Figure EV1, etc. instead of Figure Expanded View 1, etc.

- Please provide a callout for Fig. EV4.

- Datasets: the nomenclature needs to be corrected to Dataset EV1 - Dataset EV9 in all places; the legends need to be removed from the Appendix file and each should be provided as a separate sheet/tab in each Excel file or added to the .txt files.

- Appendix: please provide it as a PDF file. The title page should only have the title and table of content with page number. Please move all methods from the Appendix to the main methods section. The correct nomenclature should be Appendix Figure S1, etc. Appendix Table S1, etc.; the callouts in the manuscript and Reagents table also need to be corrected.

- Regarding the Author Contributions, we use CRediT to specify the contributions of each author in the journal submission system. CRediT replaces the author contribution section, which therefore needs to be removed from the manuscript text. You can use the free text box in our system if you wish to provide more detailed descriptions. See also guide to authors https://www.embopress.org/page/journal/14693178/authorguide#authorshipguidelines.

- Author Checklist: please select responses to the pull down menus in column D (Yes, Not Applicable).

- Author Checklist: "Studies involving human participants". You chose "not applicable" but have used paraffin-embedded first-trimester placental tissue specimens, as correctly acknowledged and described in the methods section. I think you should therefore refer to the information in the methdos section as answer to these questions (line 92, line 93)? Please also provide the approval number in addition to the approval statement in the methods section.

- Author Checklist, line 59, applies to the housing and husbandry conditions used for the Holtzman Sprague-Dawley rats.

- Should this statement in the methods, line 374-375 be supported by a reference? "Human TS cell lines were maintained in the stem cell state and differentiated into EVT cells as previously reported"

- The information on funding in the manuscript and in the online submission system must be congruent. The latter is being transferred to PubMed. Therefore, please add the following fund(s) in the system: Program of the Joint Usage/Research Center for Developmental Medicine and High Depth Omics, IMEG, Kumamoto University

- Scott et al, 2022 and Vu et al, 2023 could be data citations. If I understand correctly you have re-analysed datasets that were reported in these studies. If you want to make use of this format, you would have to add a data citation in addition to the current reference. The data citation has the same authors, but instead of referring to the journal article, you add the URL that resolves to the dataset deposited at a public database. This citation needs to be tagged with [DATASET] in the reference list and with "dataref:" in the text [(Scott et al, 2022; dataref: Scott et al, 2022)].

- Our data editors kindly ask you to attend to the following points in the figure legends:
- Please note that the exact p values are not provided in the legends of figures 1C, 2A, C; 3C, 4C, 6D, 7D, 8C, D, F, H; EV1 A, D; EV2 A, C; EV3 B, D, G, I; EV4 D, EV5
- Please indicate the statistical test used for data analysis in the legends of figures 5E, F
- Please note that information related to n is missing in the legends of figures 3C, 4C
- Please note that the dashed outline is not defined in the legend of figure 2C, 6A, B, E; 8A, B, E, G; EV3 E, J; EV5. This needs to be rectified.
- Please note that the white arrows are not defined in the legend of figure EV3 C, H. This needs to be rectified.
- Please note that the black arrows are not defined in the legend of figure EV2 B. This needs to be rectified.

- As a standard procedure we edit title and abstracts to make them more accessible to our general readership. Please find my suggestion below my signature and feel free to modify it further as you see best fit.

- Finally, EMBO Reports papers are accompanied online by
A) a short (1-2 sentences) summary of the findings and their significance,
B) 2-3 bullet points highlighting key results and
C) a schematic summary figure that provides a sketch of the major findings (not a data image).
Please provide the summary figure as a separate file in PNG or JPG format at a size of 550x300 pixels (width x height). Please note that the size is rather small and that text needs to be readable at the final size. Please send us this information along with the revised manuscript.

We look forward to seeing a final version of your manuscript as soon as possible.

With kind regards,

Martina Rembold, PhD
Senior Editor
EMBO reports

=================

Referee #1:

The authors have addressed all issues raised in the initial review. This manuscript is suitable for publication without further revision.

Referee #2:

In the revised manuscript "PPARG directs trophoblast cell fate and establishment of the uterine-placental interface," the authors have implemented minor, primarily cosmetic revisions, including text rephrasing (e.g., lines 99-100, 102-105, 155-156, and 160-161) and adjustments to figures (e.g., Fig.4, Fig.5, and EV4D). However, the substantive concerns raised in the initial review remain largely unaddressed. Except for including a second PPARG shRNA in their analysis and partial validation in an additional hTSC line (CT29, XY), the authors did not carry out the key experiments that were explicitly recommended (e.g., addressing the role of PPARG in STB differentiation, additional bioinformatic analysis, better characterization of the Pparg-KO rat TSCs, and identification of genes regulated by Pparg in the rat invasive trophoblast).
Furthermore, the manuscript fails to adequately acknowledge (except for a brief mention in the Discussion section) a recently published study reporting highly similar findings by Guo et al. (PMID: 40705926), which is essential for contextualizing the novelty and contribution of the present work.
Overall, the revisions represent only incremental improvements over the original submission and do not sufficiently address concerns previously raised. Substantial additional experimental and analytical work is required to support the manuscript's conclusions.

================

Feedback from referee #1 on your point-by-point response:

The complementary review asserts primarily that:

"... the authors have implemented minor, primarily cosmetic revisions ... Except for including a second PPARG shRNA in their analysis and partial validation in an additional hTSC line (CT29, XY) , the authors did not carry out the key experiments that were explicitly recommended ..."

and

"the manuscript fails to adequately acknowledge (except for a brief mention in the Discussion section) a recently published study reporting highly similar findings by Guo et al. (PMID: 40705926),"

In my view both the original and revised manuscripts include experiments that substantially extend the observations recently reported by Guo, et al. and the new data in the revision is responsive to the initial reviews and are not fairly characterized as merely "cosmetic". Also, the unexpected essential roles of PPARγ in trophoblast differentiation and placental development, including roles in both trophoblast stem cell maintenance and differentiation, were initially reported 18 and 27 years ago, respectively. The "novelty" and "priority" of corresponding assertions by Guo, et al are relative and should be understood and presented in this context.

The in vivo studies in rats in the manuscript under consideration are significant beyond mere confirmation because, needless to say, they demonstrate effects in whole animals including accounting for the variability that is inherent in the in vivo context. This is a major contribution that I believe is not adequately reflected in the negative review. In contrast, Guo, et al present the results of ex vivo analysis of single nuclei from only a single human TSC line established by others after disruption of the first trimester human gestational context with possible in vivo and ex vivo artifacts introduced prior to analysis. Mechanistic insights from their more moleculary specific analyses are useful but, since the majority of the mechanistic "conclusions" did not involve interventional testing of specific hypotheses, those primarily provide directions for future studies, and the "real-world" significance of any of the observations remains to be tested in vivo.

In addition, the original data presented in Guo, et al was obtained, as far as I can tell, using a single human TSC cell line, XX CT27. This line is included, along with a second human TSC line, XY CT29, in the current data set of the revised manuscript under consideration. In addition to substantially demonstrating replicability, including both male and female TSC lines begins to address sex as a biological variable, albeit at a rudimentary level.

I also feel that addressing the role PPARγ in invasive trophoblast is sufficient. Extending the analysis to STB, as requested, would represent an entirely new direction, with substantially different experimental protocols reflecting the functional biological differences of invasive and syncytial trophoblast, that would distract from, not enhance, the primary contribution of these studies. This would not be a matter of simply applying the same analyses to a different tissue and cell type. I think this would require a different, not a revised, manuscript.

=================

Suggested abstract:

The expansion and differentiation of trophoblast stem (TS) cells are critical for defining fundamental properties of the placenta. Specialized trophoblast cells called extravillous trophoblast (EVT) cells in human and invasive trophoblast cells in rat, exit the placenta,enter and transform the uterus. This includes a restructuring of uterine spiral arteries, essential for the transfer of nutrients to the fetus Mechanisms governing invasive trophoblast cell differentiation remain poorly understood. Here, we investigate peroxisome proliferator-activated receptor gamma (PPARG) as a potential regulator of EVT/invasive trophoblast cell development. In first trimester human placentas, PPARG is expressed in the EVT cell column and increases in amount as human TS cells differentiate into EVT cells. PPARG disruption impairs EVT cell differentiation and alters the expression of genes controling EVT lineage development. Rat invasive trophoblast cells similarly express PPARG. Conditional inactivation of PPARG within rat invasive trophoblast cells establishes PPARG as an essential cell-autonomous regulator of invasive trophoblast cells in vivo. In conclusion, PPARG is a conserved regulator of placentation and is essential for directing trophoblast cell-guided uterine transformation.

Given the feedback and support by referee #1 and the considerations noted above, I have decided to proceed with publication of your manuscript, under the condition that the remaining concerns raised are acknowledged in the manuscript and adequately discussed.

We appreciate the support from referee #1 and the efforts of the editor in facilitating the publication of our research.

From the editorial side there are also a number of things that we need before we can proceed with the official acceptance of your study.

We have addressed each of the issues from the editorial side (see below).

- Please place the Disclosure and competing interests statement' after the Acknowledgments.

We have inserted the 'Disclosure and Competing Interests Statement' as requested.

- Please correct the following name discrepancy: Ayelen Moreno-Irusta in the manuscript vs. Ayelen Moreno in the submission system.

We have corrected the name discrepancy in the submission system as requested.

- "Methods and Protocols" should be "Methods".

We have changed "Methods and Protocols" to "Methods" as requested.

- Nomenclature of EV figures: it should be Figure EV1, etc. instead of Figure Expanded View 1, etc.

We have changed the nomenclature for the EV figures as requested.

- Please provide a callout for Fig. EV4.

We have provided a callout for Fig. EV4 as requested.

- Datasets: the nomenclature needs to be corrected to Dataset EV1 - Dataset EV9 in all places; the legends need to be removed from the Appendix file and each should be provided as a separate sheet/tab in each Excel file or added to the .txt files.

We have changed the nomenclature for the Datasets and moved the legends to a separate sheet in each Excel file.

- Appendix: please provide it as a PDF file. The title page should only have the title and table of content with page number. Please move all methods from the Appendix to the main methods section. The correct nomenclature should be Appendix Figure S1, etc.

Appendix Table S1, etc.; the callouts in the manuscript and Reagents table also need to be corrected.

The Appendix has been modified as requested.

- Regarding the Author Contributions, we use CRediT to specify the contributions of each author in the journal submission system. CRediT replaces the author contribution section, which therefore needs to be removed from the manuscript text. You can use the free text box in our system if you wish to provide more detailed descriptions. See also guide to authors https://www.embopress.org/page/journal/14693178/authorguide#authorshipguidelines.

We have removed the Author Contributions section and used CRediT to specify the contributions of each author.

- Author Checklist: please select responses to the pull down menus in column D (Yes, Not Applicable).

Completed as requested.

- Author Checklist: "Studies involving human participants". You chose "not applicable" but have used paraffin-embedded first-trimester placental tissue specimens, as correctly acknowledged and described in the methods section. I think you should therefore refer to the information in the methdos section as answer to these questions (line 92, line 93)? Please also provide the approval number in addition to the approval statement in the methods section.

Modified as requested.

- Author Checklist, line 59, applies to the housing and husbandry conditions used for the Holtzman Sprague-Dawley rats.

Line 59 was corrected in the check list file, and regarding information is referred in Methods.

- Should this statement in the methods, line 374-375 be supported by a reference? "Human TS cell lines were maintained in the stem cell state and differentiated into EVT cells as previously reported"

We have added a reference as requested.

- The information on funding in the manuscript and in the online submission system must be congruent. The latter is being transferred to PubMed. Therefore, please add the following fund(s) in the system: Program of the Joint Usage/Research Center for

Developmental Medicine and High Depth Omics, IMEG, Kumamoto University

Funding information in the two locations are now in agreement as requested.

- Scott et al, 2022 and Vu et al, 2023 could be data citations. If I understand correctly you have re-analysed datasets that were reported in these studies. If you want to make use of this format, you would have to add a data citation in addition to the current reference. The data citation has the same authors, but instead of referring to the journal article, you add the URL that resolves to the dataset deposited at a public database. This citation needs to be tagged with [DATASET] in the reference list and with "dataref:" in the text [(Scott et al, 2022; dataref: Scott et al, 2022)].

Data set reference was modified as recommended.

- Our data editors kindly ask you to attend to the following points in the figure legends:

- Please note that the exact p values are not provided in the legends of figures 1C, 2A, C; 3C, 4C, 6D, 7D, 8C, D, F, H; EV1 A, D; EV2 A, C; EV3 B, D, G, I; EV4 D, EV5
- Please indicate the statistical test used for data analysis in the legends of figures 5E, F
- Please note that information related to n is missing in the legends of figures 3C, 4C
- Please note that the dashed outline is not defined in the legend of figure 2C, 6A, B, E; 8A, B, E, G; EV3 E, J; EV5. This needs to be rectified.
- Please note that the white arrows are not defined in the legend of figure EV3 C, H. This needs to be rectified.
- Please note that the black arrows are not defined in the legend of figure EV2 B. This needs to be rectified.

Each of the issues with the figure legends has been addressed as requested.

- As a standard procedure we edit title and abstracts to make them more accessible to our general readership. Please find my suggestion below my signature and feel free to modify it further as you see best fit.

Thank you for your suggested modifications to the abstract. We have made some minor additional edits to the abstract.

- Finally, EMBO Reports papers are accompanied online by
A) a short (1-2 sentences) summary of the findings and their significance,
B) 2-3 bullet points highlighting key results and
C) a schematic summary figure that provides a sketch of the major findings (not a data image). Please provide the summary figure as a separate file in PNG or JPG format at a size of 550x300 pixels (width x height). Please note that the size is rather small and that text needs to be readable at the final size. Please send us this information along with the revised manuscript.

We have included a short summary of the findings and their significance, bullet points highlighting the key results, and a schematic figure of the major findings as requested.

================

Feedback from referee #1 on your point-by-point response:

The "novelty" and "priority" of corresponding assertions by Guo, et al are relative and should be understood and presented in this context.

We have indicated that Guo and co-workers recently published research demonstrating the involvement of PPARG in EVT cell differentiation.

See lines 332-333:

…Similarly, using human TS cells, Guo and co-workers recently demonstrated the involvement of PPARG in EVT cell differentiation (Guo *et al*, 2025)…

Dr. Esteban Dominguez
University of Kansas Medical Center
Department of Pathology & Laboratory Medicine
3901 Rainbow Blvd
3050
Kansas City, KS 66160
United States

Dear Esteban,

Thank you for implementing the final changes in the manuscript text and synopsis. I am now very pleased to accept your manuscript for publication in the next available issue of EMBO reports. Thank you for your contribution to our journal.

Your manuscript will be processed for publication by EMBO Press. It will be copy edited and you will receive page proofs prior to publication. Please note that you will be contacted by Springer Nature Author Services to complete licensing and payment information.

You may qualify for financial assistance for your publication charges - either via a Springer Nature fully open access agreement or an EMBO initiative. Check your eligibility: https://link.springer.com/journal/44319/how-to-publish-with-us

Should you be planning a Press Release on your article, please notify the production team via this form in order to coordinate publication and release dates: https://forms.cloud.microsoft/e/eH4A8JVP6g

If you have any questions, please do not hesitate to contact the Editorial Office. Thank you for your contribution to EMBO Reports.

Kind regards,

Martina

Martina Rembold, PhD
Senior Editor
EMBO reports

>>> Please note that it is EMBO Reports policy for the transcript of the editorial process (containing referee reports and your response letter) to be published as an online supplement to each paper. If you do NOT want this, you will need to inform the Editorial Office via email immediately. More information is available here: https://link.springer.com/partners/embo-press/editorial-policies#Peer%20review

EMBO Press Author Checklist

Corresponding Author Name: Esteban M. Dominguez, Michael J. Soares	
Journal Submitted to: EMBO reports	
Manuscript Number: 2025-63189	

USEFUL LINKS FOR COMPLETING THIS FORM
The EMBO Journal - Author Guidelines
EMBO Reports - Author Guidelines
Molecular Systems Biology - Author Guidelines
EMBO Molecular Medicine - Author Guidelines

Reporting Checklist for Life Science Articles (updated January
This checklist is adapted from Materials Design Analysis Reporting (MDAR) Checklist for Authors. MDAR establishes a minimum set of requirements in transparent reporting in the life sciences (see Statement of Task: 10.31222/osf.io/9sm4x). Please follow the journal's guidelines in preparing your
Please note that a copy of this checklist will be published alongside your article.

Abridged guidelines for figures
1. Data
The data shown in figures should satisfy the following conditions:
- the data were obtained and processed according to the field's best practice and are presented to reflect the results of the experiments in an accurate and unbiased manner.
- ideally, figure panels should include only measurements that are directly comparable to each other and obtained with the same assay.
- plots include clearly labeled error bars for independent experiments and sample sizes. Unless justified, error bars should not be shown for technical
- if n<5, the individual data points from each experiment should be plotted. Any statistical test employed should be justified.
- Source Data should be included to report the data underlying figures according to the guidelines set out in the authorship guidelines on Data

2. Captions
Each figure caption should contain the following information, for each panel where they are relevant:
- a specification of the experimental system investigated (eg cell line, species name).
- the assay(s) and method(s) used to carry out the reported observations and measurements.
- an explicit mention of the biological and chemical entity(ies) that are being measured.
- an explicit mention of the biological and chemical entity(ies) that are altered/varied/perturbed in a controlled manner.
- the exact sample size (n) for each experimental group/condition, given as a number, not a range;
- a description of the sample collection allowing the reader to understand whether the samples represent technical or biological replicates (including how many animals, litters, cultures, etc.).
- a statement of how many times the experiment shown was independently replicated in the laboratory.
- definitions of statistical methods and measures:
  - common tests, such as t-test (please specify whether paired vs. unpaired), simple χ2 tests, Wilcoxon and Mann-Whitney tests, can be unambiguously identified by name only, but more complex techniques should be described in the methods section;
  - are tests one-sided or two-sided?
  - are there adjustments for multiple comparisons?
  - exact statistical test results, e.g., P values = x but not P values < x;
  - definition of 'center values' as median or average;
  - definition of error bars as s.d. or s.e.m.

Please complete ALL of the questions below.
Select "Not Applicable" only when the requested information is not relevant for your study.

Materials

Newly Created Materials	Information included in the manuscript?	In which section is the information available? (Reagents and Tools Table, Materials and Methods, Figures, Data Availability Section)
New materials and reagents need to be available; do any restrictions apply?	Not Applicable	

Antibodies	Information included in the manuscript?	In which section is the information available? (Reagents and Tools Table, Materials and Methods, Figures, Data Availability Section)
For **antibodies** provide the following information:<br>- Commercial antibodies: RRID (if possible) or supplier name, catalogue number and or/clone number<br>- Non-commercial: RRID or citation	Yes	Materials and Methods/Reagents and Tools Table

DNA and RNA sequences	Information included in the manuscript?	In which section is the information available? (Reagents and Tools Table, Materials and Methods, Figures, Data Availability Section)
**Short novel DNA or RNA including primers, probes**: provide the sequences.	Yes	Appendix table S4; S5 and S6

Cell materials	Information included in the manuscript?	In which section is the information available? (Reagents and Tools Table, Materials and Methods, Figures, Data Availability Section)
**Cell lines:** Provide species information, strain. Provide accession number in repository **OR** supplier name, catalog number, clone number, and/**OR** RRID.	Yes	Materials and Methods/Reagents and Tools Table
**Primary cultures:** Provide species, strain, sex of origin, genetic modification status.	Not Applicable	
Report if the cell lines were recently **authenticated** (e.g., by STR profiling) and tested for mycoplasma contamination.	Not Applicable	

Experimental animals	Information included in the manuscript?	In which section is the information available? (Reagents and Tools Table, Materials and Methods, Figures, Data Availability Section)
**Laboratory animals or Model organisms:** Provide species, strain, sex, age, genetic modification status. Provide accession number in repository **OR** supplier name, catalog number, clone number, **OR** RRID.	Yes	Materials and Methods/Reagents and Tools Table
**Animal observed in or captured from the field:** Provide species, sex, and age where possible.	Not Applicable	
Please detail **housing and husbandry conditions**.	Yes	Materials and Methods

Plants and microbes	Information included in the manuscript?	In which section is the information available? (Reagents and Tools Table, Materials and Methods, Figures, Data Availability Section)
**Plants:** provide species and strain, ecotype and cultivar where relevant, unique accession number if available, and source (including location for collected wild specimens).	Not Applicable	
**Microbes:** provide species and strain, unique accession number if available, and source.	Not Applicable	

Human research participants	Information included in the manuscript?	In which section is the information available? (Reagents and Tools Table, Materials and Methods, Figures, Data Availability Section)
If collected and within the bounds of privacy constraints report on age, sex and gender or ethnicity for all study participants.	Not Applicable	

Core facilities	Information included in the manuscript?	In which section is the information available? (Reagents and Tools Table, Materials and Methods, Figures, Data Availability Section)
If your work benefited from core facilities, was their service mentioned in the acknowledgments section?	Not Applicable	

Design

Study protocol	Information included in the manuscript?	In which section is the information available? (Reagents and Tools Table, Materials and Methods, Figures, Data Availability Section)
If study protocol has been **pre-registered, provide DOI in the manuscript**. For clinical trials, provide the trial registration number **OR** cite DOI.	Not Applicable	
Report the **clinical trial registration number** (at ClinicalTrials.gov or equivalent), where applicable.	Not Applicable	

Laboratory protocol	Information included in the manuscript?	In which section is the information available? (Reagents and Tools Table, Materials and Methods, Figures, Data Availability Section)
Provide DOI OR other citation details if **external detailed step-by-step protocols** are available.	Not Applicable	

Experimental study design and statistics	Information included in the manuscript?	In which section is the information available? (Reagents and Tools Table, Materials and Methods, Figures, Data Availability Section)
Include a statement about **sample size** estimate even if no statistical methods were used.	Yes	Materials and Methods
Were any steps taken to minimize the effects of subjective bias when allocating animals/samples to treatment (e.g. **randomization procedure**)? If yes, have they been described?	Yes	Materials and Methods
Include a statement about **blinding** even if no blinding was done.	Yes	Materials and Methods
Describe **inclusion/exclusion criteria** if samples or animals were excluded from the analysis. Were the criteria pre-established?<br><br>If sample or data points were omitted from analysis, report if this was due to attrition or intentional exclusion and provide justification.	Yes	Materials and Methods
For every figure, are **statistical tests** justified as appropriate? Do the data meet the assumptions of the tests (e.g., normal distribution)? Describe any methods used to assess it. Is there an estimate of variation within each group of data? Is the variance similar between the groups that are being statistically compared?	Yes	Materials and Methods

Sample definition and in-laboratory replication	Information included in the manuscript?	In which section is the information available? (Reagents and Tools Table, Materials and Methods, Figures, Data Availability Section)
In the figure legends: state number of times the experiment was **replicated** in laboratory.	Yes	Figure legends
In the figure legends: define whether data describe **technical or biological replicates**.	Yes	Figure legends

Ethics

Ethics	Information included in the manuscript?	In which section is the information available? (Reagents and Tools Table, Materials and Methods, Figures, Data Availability Section)
Studies involving **human participants**: State details of **authority granting ethics approval** (IRB or equivalent committee(s), provide reference number for approval.	Yes	Materials and Methods
Studies involving **human participants**: Include a statement confirming that **informed consent** was obtained from all subjects and that the experiments conformed to the principles set out in the WMA Declaration of Helsinki and the Department of Health and Human Services Belmont Report.	Yes	Materials and Methods
Studies involving **human participants:** For publication of **patient photos**, include a statement confirming that consent to publish was obtained.	Not Applicable	
Studies involving experimental **animals**: State details of **authority granting ethics approval** (IRB or equivalent committee(s), provide reference number for approval. Include a statement of compliance with ethical regulations.	Yes	Materials and Methods
Studies involving **specimen and field samples:** State if relevant **permits** obtained, provide details of authority approving study; if none were required, explain why.	Not Applicable	

Dual Use Research of Concern (DURC)	Information included in the manuscript?	In which section is the information available? (Reagents and Tools Table, Materials and Methods, Figures, Data Availability Section)
Could your study fall under dual use research restrictions? Please check biosecurity documents and list of **select agents and toxins** (CDC): https://www.selectagents.gov/sat/list.htm	Not Applicable	
If you used a select agent, is the security level of the lab appropriate and reported in the manuscript?	Not Applicable	
If a study is subject to dual use research of concern regulations, is the name of the **authority granting approval and reference number** for the regulatory approval provided in the manuscript?	Not Applicable	

Reporting

The MDAR framework recommends adoption of discipline-specific guidelines, established and endorsed through community initiatives. Journals have their own policy about requiring specific guidelines and recommendations to complement MDAR.

Adherence to community standards	Information included in the manuscript?	In which section is the information available? (Reagents and Tools Table, Materials and Methods, Figures, Data Availability Section)
State if relevant guidelines or checklists (e.g., **ICMJE, MIBBI, ARRIVE, PRISMA**) have been followed or provided.	Not Applicable	
For **tumor marker prognostic studies,** we recommend that you follow the **REMARK** reporting guidelines (see link list at top right). See author guidelines, under 'Reporting Guidelines'. Please confirm you have followed these guidelines.	Not Applicable	
For **phase II and III randomized controlled trials**, please refer to the **CONSORT** flow diagram (see link list at top right) and submit the CONSORT checklist (see link list at top right) with your submission. See author guidelines, under 'Reporting Guidelines'. Please confirm you have submitted this list.	Not Applicable	

Data Availability

Data availability	Information included in the manuscript?	In which section is the information available? (Reagents and Tools Table, Materials and Methods, Figures, Data Availability Section)
Have **primary datasets** been deposited according to the journal's guidelines (see 'Data Deposition' section) and the respective accession numbers provided in the Data Availability Section?	Yes	Material and Methods/Data Availabillity Section
Were **human clinical and genomic datasets** deposited in a public access-controlled repository in accordance to ethical obligations to the patients and to the applicable consent agreement?	Not Applicable	
Are **computational models** that are central and integral to a study available without restrictions in a machine-readable form? Were the relevant accession numbers or links provided?	Not Applicable	
If publicly available data were reused, provide the respective **data citations in the reference list**.	Not Applicable	
